# Biostimulant-Mediated Enhancement of Growth, Yield, and Abiotic Stress Tolerance in Wheat and Barley: Mechanisms, Research Gaps, and Future Perspectives

**DOI:** 10.3390/plants15213382

**Published:** 2026-11-01

**Authors:** Pyae Pyae Win, Yong In Kuk

**Affiliations:** Department of Bio-Oriental Medicine Resources, Sunchon National University, Suncheon 57922, Republic of Korea; winpyae581@gmail.com

**Keywords:** salicylic acid, melatonin, silicon, reactive oxygen species, photosynthetic performance, osmotic adjustment, antioxidant defense, transcriptional regulation

## Abstract

Wheat (*Triticum aestivum* L.) and barley (*Hordeum vulgare* L.) are crucial cereal crops worldwide, facing increasing challenges from abiotic stresses such as heat, cold, drought, salinity, and waterlogging. Biostimulants present a promising strategy to enhance stress tolerance by influencing interconnected physiological, biochemical, and molecular mechanisms. This review synthesizes evidence on biostimulant-mediated stress tolerance in these cereals using five functional categories: phytohormones and signaling molecules; osmoprotectants and antioxidants; silicon, mineral nutrients, and nanoparticles; humic substances, seaweed, and plant extracts; and beneficial microorganisms. Across stress conditions, biostimulants generally improved photosynthetic performance, redox homeostasis, osmotic and ionic balance, and membrane stability. Molecular evidence further implicates antioxidant defense, hormone signaling, ion transport, aquaporin, and cold-responsive pathways, although mechanistic validation remains limited. Salicylic acid, melatonin, and silicon were among the most supported agents, whereas evidence for waterlogging and microbial biostimulants was comparatively scarce. Treatment efficacy varied with genotype, developmental stage, dose, and stress intensity and duration. Overall, biostimulants offer promising opportunities to improve stress tolerance and climate resilience in wheat and barley; however, multi-location field trials and omics-based investigations are needed to validate their effectiveness and elucidate underlying mechanisms for sustainable, stress-resilient crop production.

## 1. Introduction

Abiotic stresses, such as drought, salinity, and extreme temperatures, are major constraints to global agricultural productivity and sustainability. These stresses are intensified by climate change, which accelerates extreme weather events and alters precipitation and temperature patterns worldwide [1]. Consequently, agricultural production in the 21st century faces unprecedented challenges in sustaining crop productivity under increasingly stressful conditions [2]. Compounding this issue, the global population continues to rise, requiring more than a 70% increase in food production to meet the demands of an additional 2.3 billion people by 2050 [3]. Wheat is one of the earliest domesticated crops, and barley is the fourth most widely cultivated cereal; together, they provide more than 20% of the dietary intake for nearly half of the global population [4,5]. Bread wheat and cultivated barley originated in the Fertile Crescent and form the basis of the agricultural revolution that began in the region approximately 10,000 years ago. However, various environmental stresses, such as salinity, drought, heat, freezing, heavy metals, and pathogen infections, greatly compromise their productivity and are a major threat to the growth and yield of wheat and barley under field conditions [6,7]. Most crops are salt-sensitive, and the average grain yield of significant glycophytic crops decreases by 50–80% under moderate salinity [8]. Globally, abiotic stresses such as high temperature (20%), low temperature (7%), salinity (10%), drought (9%), and other stresses (4%) cause an average yield loss of 50% in agricultural crops [9].

Abiotic stress causes significant damage to tissues, cells, and molecular structures, leading to changes in growth patterns and declines in both photosynthetic performance and reproductive output [10,11]. Plants exploit complex defense mechanisms regulated by diverse signaling molecules, transcription factors, and stress-responsive genes. Each stressor induces distinct and overlapping responses that can be synergistic or antagonistic [12]. A common feature of these responses is the excessive accumulation of reactive oxygen species (ROS), including singlet oxygen, superoxide anions (O_2_•^−^), hydrogen peroxide (H_2_O_2_), and hydroxyl radicals (·OH), which damage proteins, lipids, carbohydrates, and nucleic acids, ultimately leading to cellular dysfunction and death if unchecked [13,14,15]. Despite their toxic effects, ROS are critical signaling molecules for the preservation of intracellular redox homeostasis and activation of antioxidant defense pathways [16]. Plants therefore rely on enzymatic antioxidants, including superoxide dismutase (SOD), peroxidase (POD), and catalase (CAT), together with nonenzymatic compounds to maintain the balance between ROS generation and scavenging [12,17,18,19].

The effect of abiotic stress depends not only on the stress type but also on its intensity, duration, frequency, and timing relative to crop development. Mild or short episodes may trigger acclimation and stress memory, as shown for cold priming in wheat [20], whereas severe, prolonged, or recurrent episodes can exceed the capacity of antioxidant and repair systems and cause irreversible damage [12]. Stress events may also interact across time: damage caused by an early event, such as a late-spring cold spell, may reduce tolerance to subsequent stresses, such as terminal heat or drought. Moreover, the effects of simultaneous stresses are often non-additive and cannot be predicted by simply summing their individual effects [12,21]. Sensitive stages such as anthesis and grain filling are particularly vulnerable, as shown for heat in wheat [22], so the timing of exposure can strongly affect yield. Consequently, biostimulant efficacy should be evaluated in the context of stress intensity, frequency, cumulative exposure, crop developmental stage, and application timing.

Plant biostimulants (PBs) have been identified as potentially viable, environment-friendly tools for improving plant defense under abiotic stress conditions [23]. Biostimulants are bioactive compounds or microorganisms that, irrespective of their nutrient content, enhance plant growth and stress tolerance by influencing physiological functions such as photosynthesis, water-use efficiency, and nutrient uptake [24,25]. Based on their origin, biostimulants are generally classified into major groups, including seaweed extracts, plant extracts, protein hydrolysates, humic substances, inorganic compounds, and microorganisms [26]. These groups act through overlapping mechanisms: modulating metabolic and enzymatic activity, enhancing antioxidant defense, promoting osmolyte and nutrient accumulation, and sustaining cellular homeostasis, which together improve agronomic performance under stress, although their efficacy depends on biostimulant type, formulation, and plant species [27,28]. Beyond their agronomic benefits, biostimulants contribute to sustainable agriculture by promoting resource-use efficiency and reducing the carbon footprint of crop production. This has driven the rapid growth of the global biostimulant market, valued at approximately USD 4 billion by 2025 [29,30], and positioned biostimulants as a focal point of agricultural research aimed at improving crop performance in stress-prone environments [31,32].

Under natural field conditions, crops are frequently exposed to multiple and interacting stresses that can severely constrain growth and productivity. Biostimulants can contribute to stress adaptation by improving nutrient utilization, photosynthetic performance, antioxidant capacity, and other physiological processes [33,34,35]. For example, free amino acid formulations can influence nitrogen and carbon metabolism, while micronutrients (including zinc, molybdenum, manganese, copper, and boron) support root development and photosynthetic activity [24,36]. Seaweed and plant-derived extracts have been widely investigated for their ability to enhance growth and tolerance to diverse abiotic stresses [33]. Overall, biostimulants promote plant development, photosynthesis, and stress resistance, and support sustainable farming by upregulating antioxidant systems while preserving ecosystem integrity [37].

Although biostimulants have been extensively studied for their capacity to assist crop plants in stress management, comprehensive and critical comparisons of their efficacy in wheat and barley under major stresses remain insufficient. Therefore, this review aims to critically synthesize current evidence on the application of both microbial and non-microbial biostimulants for improving growth, physiological performance, and yield of wheat and barley under drought, salinity, waterlogging, and high- and low-temperature stresses. Biostimulants are grouped into a common classification framework so that responses can be compared across stresses and species. The review highlights the integration of physiological, biochemical, signaling, and molecular responses, with a dedicated section on transcriptional regulation. It assesses the impact of biostimulant type, genotype, developmental stage, application method, concentration, and stress severity. Furthermore, it treats evidence from controlled environments as the mechanistic foundation for field validation. The review concludes by identifying knowledge gaps and research opportunities for optimizing biostimulant application and promoting their practical integration into sustainable and climate-resilient wheat and barley production.

## 2. Scope and Classification Framework

This review synthesizes peer-reviewed research on wheat and barley, sourced from Web of Science, Scopus, PubMed, and Google Scholar, using combinations of the terms “biostimulant,” “wheat,” “barley,” and specific abiotic stress terms from 1998 to 2026. Studies were included if they involved the application of a particular biostimulant to wheat or barley under specified abiotic stress conditions, with reported outcomes on growth, physiological, biochemical, molecular, or yield responses. Given the substantial variation in treatments, doses, genotypes, and traits measured across studies, the evidence is presented narratively, with detailed information provided in the accompanying tables. When physiological, biochemical, and molecular evidence converged, the conclusions regarding mechanisms were considered more robust; otherwise, mechanisms are described as proposed or associated responses.

For consistency across the stress-specific sections, biostimulants are grouped into five functional categories: (A) phytohormones and signaling molecules (salicylic acid, jasmonic acid, abscisic acid, gibberellins, brassinosteroids, strigolactones, auxin and ethylene modulators, melatonin, nitric oxide, and hydrogen sulfide); (B) osmoprotectants and antioxidant compounds (glycine betaine, proline, ascorbic acid, glutathione, polyamines, and sugars); (C) silicon, mineral nutrients, and nanoparticles (silicon, zinc, selenium, molybdenum, potassium, and nitrogen, as well as zinc-, iron-, titanium-, silver-, selenium-, and silicon-based nanomaterials); (D) humic and fulvic substances, seaweed extracts, and plant extracts; and (E) beneficial microorganisms (arbuscular mycorrhizal fungi and plant growth-promoting fungi and bacteria). This classification was adopted to organize the review and does not replace established distinctions between microbial and non-microbial biostimulants. Crop–biostimulant combinations for which no eligible evidence was identified are indicated as “None reported” in the tables. Molecular and transcriptional mechanisms are discussed in Section 8. Responses to stress and biostimulants are influenced by specific contexts. Wheat genotypes differ in heat tolerance and yield stability [22,38,39,40], and winter-type cereals generally exhibit greater cold acclimation than spring types [41,42]. Accordingly, these factors are considered throughtout the stress-specific sections, with genotype × environment × biostimulant interactions recognized as an important source of variation in the magnitude and consistency of reported responses.

## 3. Biostimulant-Mediated Tolerance to High-Temperature Stress in Wheat and Barley

### 3.1. Effects of High Temperature and the Role of Genotype

Global warming is one of the most important factors leading to a decrease in agronomic productivity worldwide, and high-temperature stress significantly threatens cereal crop production [43]. Wheat, a temperate crop that grows optimally at 23–25 °C, is responsive to temperature, particularly terminal heat stress during the reproductive and grain-filling stages [38]. More than 50% of the wheat-growing areas worldwide are predicted to face suboptimal temperature conditions, imposing significant reductions in yield [39]. In both wheat and barley, high-temperature stress reduces seedling emergence, cell division and elongation, photosynthetic efficiency, water status, membrane stability, grain-filling duration, and biomass accumulation, ultimately reducing grain yield [22,40,44]. Moreover, high temperatures cause excessive generation of ROS, resulting in oxidative damage, membrane destabilization, and impairment of cellular metabolism [45,46,47].

The severity of heat-induced effects depends strongly on the genotype and environment. Spring wheat cultivars selected for tropical and subtropical environments may maintain growth and yield under high temperatures through traits such as stable photosynthesis, membrane integrity, and efficient grain filling; therefore, their responses should not be assumed to be those of cultivars adapted to a temperate environment [39,40]. Substantial differences in yield stability among commercial cultivars exposed to terminal heat stress have also been documented [38]. Heat-stress responses are further influenced by the thermal regime, including day and night temperatures, exposure duration, and concurrent stresses such as drought or high irradiance, as well as by the developmental stage at which stress occurs. Across the studies reviewed, experimental temperatures ranged from moderate (30–35 °C) to extreme (42–45 °C), and most experiments used seedlings of a single cultivar (Table 1), limiting the generalizability of the findings to reproductive development and field conditions. Biostimulant efficacy may depend on the genotype’s intrinsic heat tolerance and the severity of stress exposure; however, genotype × environment × biostimulant interaction remains insufficiently investigated.

### 3.2. Wheat: Biostimulant Responses by Category

#### 3.2.1. Phytohormones and Signaling Molecules

In wheat, presoaking or foliar application of SA improved high-temperature tolerance under 36–40 °C conditions, leading to increased grain yield through the accumulation of chlorophyll pigments, soluble proteins, proline, and sugars at both the seedling and anthesis stages [48]. Foliar application of SA (0.1 mmol L^−1^) at the heading and anthesis stages increased wheat performance under 15–30 °C conditions by promoting photosynthetic characteristics, affecting enzyme activity and dry matter distribution, and reducing yield loss [49]. The application of SA and abscisic acid (ABA) enhanced antioxidant enzyme activity and increased ascorbate and α-tocopherol levels, reducing lipid peroxidation and improving thermotolerance in wheat [50]. SA also protected the photosynthetic apparatus and maintained PSII efficiency and Rubisco under high-temperature stress [51,52]. Combined application of nitric oxide (NO, 50 μM) and SA (0.5 mM) further improved sulfur assimilation, antioxidant activity, and photosynthetic performance while reducing oxidative damage in wheat at 40 °C [53]. Melatonin (MT) application at 100 μM increased wheat tolerance to 42 °C by reducing ROS accumulation, enhancing antioxidant enzyme activity, activating the ascorbate–glutathione (AsA–GSH) cycle, increasing proline accumulation, and regulating stress-responsive genes [54]. MT has also contributed to methyl jasmonate (MeJA)-induced high-temperature tolerance through improved sulfur assimilation, antioxidant activity, and photosynthetic performance [55].

#### 3.2.2. Silicon, Mineral Nutrients, and Nanoparticles

Among the biostimulants, silicon (Si) has received increasing attention because of its multifunctional role in alleviating both biotic and abiotic stresses [56,57]. In wheat, foliar application of Si (4 mM) at the heading stage improved photosynthetic pigments, antioxidant enzyme activities, osmoprotectants, and grain yield under terminal high-temperature stress [57]. Seed treatment with Si (1.5 mM) and Si nanoparticles (1.66 mM) improved wheat tolerance to 45 °C by reducing cellular damage, enhancing photosynthesis, and stabilizing membranes, with SiNPs being more effective because of aquaporin gene regulation and improved water retention [58]. Combined Si and 24-epibrassinolide application further enhanced wheat growth, photosynthesis, antioxidant activity, and osmoprotectant accumulation, thereby alleviating the adverse effects of high-temperature conditions [59]. Nanoparticle-based nutrients have also shown potential in wheat. Foliar application of ZnNPs (10 ppm) and FeNPs (0.25 ppm) significantly increased wheat survival and yield at 38 °C by improving antioxidant enzyme activities, inducing new isozyme bands, and reducing lipid peroxidation in the Gimmeza7 cultivar [60]. Zinc oxide (ZnO) and TiO_2_ nanoparticles at appropriate concentrations enhanced antioxidant activities, phenolic compounds, and membrane stability while reducing H_2_O_2_ and malondialdehyde (MDA) accumulation [61], and morinda-derived silver nanoparticles (AgNPs) improved root and shoot growth and biomass [62]. However, nanoparticle responses are strongly concentration- and genotype-dependent, and excessive concentrations may become detrimental [63].

#### 3.2.3. Humic Substances and Seaweed and Plant Extracts

Moringa leaf extract (MLE) and sorghum water extract (SWE) improved the growth and stress-associated biochemical responses of wheat under heat conditions [64]. The effectiveness of MLE is attributed to its richness in zeatin, ascorbic acid (AsA), and other growth-promoting compounds, which enhance plant metabolism and stress tolerance [65].

### 3.3. Barley: Biostimulant Responses by Category

#### 3.3.1. Phytohormones and Signaling Molecules

In barley, foliar application of SA (2 mM) improved tolerance to 42 °C by improving growth, photosynthesis, antioxidant activity, and proline accumulation, while reducing MDA and oxidative stress [43]; earlier studies similarly showed that SA increased photosynthetic activity and reduced membrane damage and lipid peroxidation [66]. In barley, plant growth regulators (PGRs), particularly gibberellic acid (GA_3_) and its combinations, significantly enhanced mitotic activity while reducing cytogenetic abnormalities in root meristems under high-temperature stress [67]. However, responses to PGRs are dose- and time-dependent. Tütünoğlu et al. [68] observed that increasing GA concentrations had cytotoxic and genotoxic effects, whereas Mansour and Kamel [69] reported that the increase in chromosomal aberrations was statistically insignificant. Similarly, exogenous triacontanol (TRIA), 24-epibrassinolide (EBR), and homobrassinolide (HBR) adversely affected chromosomal behavior in barley meristematic cells under optimal conditions [70,71]. Although the response of PGRs to stress factors during cell division is not fully understood, priming is known to enhance resistance to stress by promoting cell division, DNA replication [72], and antioxidative defense [73].

#### 3.3.2. Osmoprotectants and Antioxidant Compounds

Glycine betaine (GB) and proline have been tested mainly in barley. GB improved heat tolerance by maintaining water status, photosynthesis, seedling vigor, and membrane stability; seed treatment with GB (20 mM) enhanced heat tolerance, whereas foliar GB and proline help to maintain PSII photochemical efficiency under high-temperature stress [44,74]. Foliar application of GB and proline enhances the heat resistance of photosystem II in barley exposed to drought and high-temperature stress (45 °C), maintaining photochemical efficiency and protecting the oxygen-evolving complex (OEC) [74].

#### 3.3.3. Silicon, Mineral Nutrients, and Nanoparticles

In barley, Si regulates osmoprotectants, antioxidant enzymes, and secondary metabolites [75]. Seed priming with Si and hydrogen sulfide enhanced barley tolerance to combined drought and high temperature (34 °C) by reducing ROS, MDA, and electrolyte leakage (EL), while increasing antioxidant enzyme activity and osmolyte accumulation [76]. Foliar application of Si (1.5 mM) significantly mitigated high-temperature-induced damage in barley by improving growth, increasing photosynthetic pigments, and strengthening antioxidant defenses [77].

### 3.4. Critical Synthesis of Wheat and Barley Responses to High-Temperature Stress

**Figure 1:**
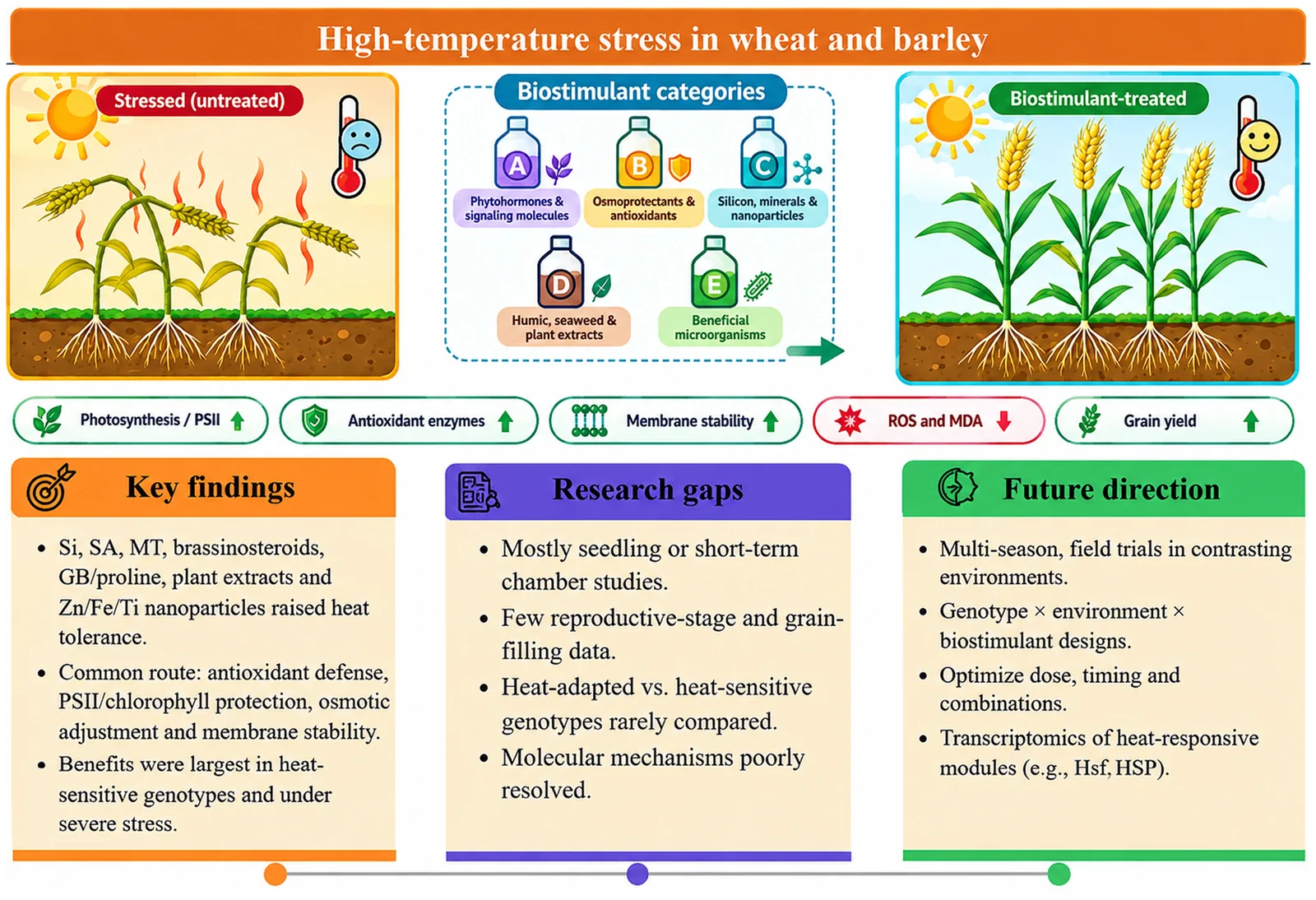
Biostimulant-mediated tolerance to high-temperature stress in wheat and barley. The illustration contrasts untreated plants under heat stress with plants treated with biostimulants of categories A–E and summarizes key findings, research gaps, and future research directions.

Figure 1 and Table 1 summarize the evidence that biostimulants, including salicylic acid (SA), silicon (Si), melatonin (MT), brassinosteroids, osmoprotectants such as proline and glycine betaine, plant extracts, and mineral- or nanoparticle-based nutrients, enhance high-temperature tolerance in wheat and barley. They act through the coordinated regulation of antioxidant defense, photosynthetic activity, osmotic adjustment, membrane stability, chlorophyll and photosystem II maintenance, and stress-related hormonal signaling, thereby mitigating heat-induced oxidative damage and promoting growth and yield. SA and Si improved heat tolerance in both crops, mainly through antioxidant defense and protection of photosynthesis. The evidence is far broader for wheat, which also covers MT, MeJA, ABA, NO, nanoparticles, and plant extracts, whereas the barley studies concentrate on osmoprotectants (GB and proline), which have not been tested in wheat. Yield was assessed only in wheat, and humic substances and beneficial microorganisms have not been tested in either crop. Responses vary considerably with biostimulant type and concentration, application method and timing, genotype, developmental stage, and stress intensity. Most existing evidence is derived from controlled environments, early growth stages, or short-term heat exposure; these studies have been essential for identifying active compounds, doses, and mechanisms and now provide the basis for reproductive-stage and field evaluation. Future research should therefore pursue multi-season field validation, comparisons of heat-adapted and heat-sensitive genotypes, optimized application protocols, combined biostimulant strategies, and a clearer characterization of the underlying molecular mechanisms.

**Table 1:** Effects of biostimulant on high-temperature stress tolerance in wheat and barley: application method, growth, yield, and mechanistic insights.

Crop	Stress Level	Biostimulants	Application Methods/ Concentrations	Growth /Yield Effect	Mechanisms	Research Gaps	References
Wheat	37 ± 2 °C	Silicon (Si)	Foliar (2 and 4 mM Si)	Yield	Enhanced photosynthetic pigments, antioxidant enzyme activities, and osmoprotectant levels	Field-scale validation missing	[57]
Wheat	45 °C	Silicon (Si) and silicon nanoparticles (SiNPs)	Seed treatment (1.5 mM Si and 1.66 mM SiNPs)	None reported	Reduced cellular damage, and improved photosynthesis and water status	Short-term; lacks yield data	[58]
Wheat	35/28 or 40/35 °C	24-epibrassinolide (EBL) and silicon (Si)	Foliar (0.8 mM Si, 0.01 μM EBL)	Growth	Enhanced photosynthetic enzyme activities and osmolyte accumulation	Combined effect mechanisms unclear	[59]
Wheat	42 °C	Melatonin (MT)	Foliar (100 μM)	None reported	Activated AsA–GSH cycle, increased proline accumulation, and regulated stress-responsive gene	Reproductive-stage evaluation absent; lack of yield data	[54]
Wheat	40 °C	Melatonin (MT), methyl jasmonate (MeJA)	Foliar (100 μM MT, 10 μM MeJA)	None reported	Enhanced sulfur assimilation, antioxidant enzyme, and photosynthesis	Field and yield validation missing	[55]
Wheat	45 °C (2 h), and 25 °C (5 days)	Salicylic acid (SA) and abscisic acid (ABA)	Spraying technique or in vitro liquid culturing technique (100 μM)	None reported	Enhanced antioxidants, ascorbate, and α-tocopherol content; reduced lipid peroxidation	No long-term validation; lack of yield data	[50]
Wheat	36–40 °C	Salicylic acid (SA)	Presoaking/ foliar (10^−4^ M)	Grain yield	Increased chlorophyll, soluble proteins, and sugar	Field confirmation missing	[48]
Wheat	15–30 °C	Salicylic acid (SA)	Foliar (0.1 mmol·L^−1^)	Grain filling	Improved photosynthetic traits and enzyme activity	Limited to vegetative stage	[49]
Wheat	40 °C, (6 h/day for 15 days)	Salicylic acid (SA), nitric oxide (NO; as sodium nitroprusside, SNP), sulfur (S)	Foliar (0.5 mM SA, 50 μM NO, 2.0 mM SO_4_^2−^)	Growth	Enhanced sulfur assimilation, photosynthesis and antioxidant enzyme activity; reduced oxidative damage	Interactions between nutrients not explored	[53]
Wheat	15–35 °C	Moringa leaf extract (MLE), sorghum water extract (SWE), salicylic acid (SA)	Foliar (3% MLE, 0.075% SWE, 10% SA)	Growth and yield	Improved stress-related biochemical traits	Lack of field data	[64]
Wheat	35–40 °C	Silver nanoparticles (AgNPs), moringa leaf extract (MLE)	Foliar (25, 50, and 75, 100 mg L^−1^ AgNPs, 15% (*w*/*v*) MLE)	Root and shoot growth, leaf traits, and biomass	None reported	Limited mechanistic insight	[62]
Wheat	38 °C	Zinc oxide nanoparticles s (80 nm), iron oxide nanoparticles (50 nm)	Foliar (0, 0.25, 0.50, 0.75, 1.00 and 10 ppm)	Yield	Enhanced antioxidant enzyme; reduced lipid peroxidation	Long-term field consistency unclear	[60]
Wheat	25 °C and 32 °C	Zinc oxide (ZnO) and titanium dioxide (TiO_2_)	Seed treatment (1.5 ppm, 10 ppm)	Seedling performance	Improved phenolic and flavanol content; reduced H_2_O_2_ and MDA levels	Limited replication; short duration	[61]
Barley	42 °C	Salicylic acid (SA)	Foliar (2 and 5 mM)	Growth	Improved photosynthesis, antioxidant activity, and proline accumulation; reduced oxidative stress and MDA levels	Short-term; field scaling required	[43]
Barley	40 ± 1/32 ± 1 °C (day/night)	Glycine betaine (GB)	Seed soaking (10, 20, 30, 40 and 50 mM)	Seedling vigor	Enhanced photosynthetic and water retention, and reduced membrane damage	Early-growth-stage focus; yield-related data lacking	[44]
Barley	45 °C + Drought	Glycine betaine (GB), proline (Pro)	Foliar (10 mM)	None reported	Enhanced osmolyte balance and photosynthetic stability	Interaction with drought unquantified; lack of yield data	[74]
Barley	Drought+34 °C	Silicon (Si) and hydrogen sulfide (H_2_S)	Seed priming (0.2, 0.4, 0.6 mM Si and 0.1, 0.3, 0.5 mM H_2_S)	Plant biomass	Reduced oxidative damage, enhanced osmolyte accumulation	Combined treatment validation missing	[76]
Barley	25/20 ± 2 °C, 35/30 ± 2 °C (day/night)	Silicon (Si)	Foliar (1.5 mM Si)	Growth	Strengthened antioxidant defense; reduced oxidative damage	Field-scale data absent	[77]
Barley	30 °C	Gibberellic acid (GA_3_), kinetin (KIN), benzyladenine (BA), 24-epibrassinolide (EBR), triacontanol (TRIA), polyamines (Spm, Spd, Put, Cad)	Seed pretreatment (1–50 μM KIN, 1–50 μM BR, 0.01–1 μM EBR, 0.1–10 μM TRIA, 10–500 μM Polyamines)	None reported	Enhanced mitotic activity and reduced cytogenetic aberrations	Early-growth-stage focus; mechanistic molecular data absent	[67]

## 4. Biostimulant-Mediated Tolerance to Low-Temperature Stress in Wheat and Barley

### 4.1. Effects of Low Temperature and the Role of Genotype

Low-temperature stress is one of the most important environmental factors that restrict the growth, development, and yield of wheat and barley [78]. Chilling and freezing temperatures hamper seed germination, photosynthesis, membrane stability, and subsequent metabolic activity, resulting in reduced establishment and crop yield [79,80]. They may also dehydrate cells and form intracellular ice crystals, causing significant cellular injury [19]. Low-temperature exposure activates the overproduction of ROS, such as superoxide radicals and hydrogen peroxide, which leads to oxidative damage, lipid peroxidation, and an imbalance of cellular homeostasis by attacking membrane lipids, proteins, and nucleic acids [81,82,83]. Plants mobilize numerous defense systems against chilling stress, including osmotic adjustment, antioxidant systems, and stress-responsive signaling pathways [84]. The impact of low temperature must be interpreted in the context of genetic adaptation and prior exposure. Winter-type and temperate-adapted wheat and barley cultivars acquire freezing tolerance through cold acclimation, which involves CBF transcription factors and cold-regulated genes [41,42]. In contrast, spring genotypes bred for tropical and subtropical environments may lack effective cold acclimation and may therefore be more vulnerable to acute cold events such as late frosts. Studies comparing barley cultivars [85,86] illustrate that biostimulant effects, such as those of SA, can differ among genotypes. Prior exposure also matters: cold priming and drought priming can increase subsequent cold tolerance, with SA and ABA involved [20,87,88]. The potential of biostimulants to mitigate low-temperature stress should therefore be evaluated in relation to genetic background, previous cold exposure, developmental stage, and the severity and duration of the stress; benefits shown in seedling experiments with chilling-sensitive material cannot be generalized to acclimated winter cereals in the field.

### 4.2. Wheat: Biostimulant Responses by Category

#### 4.2.1. Phytohormones and Signaling Molecules

Among the biostimulants, MT functions as an important signaling molecule that regulates diverse physiological processes in plants [89], including seed germination [90], plant growth [91], and grain filling [92]. In wheat, foliar application of MT during recovery increases cold priming-induced tolerance to subsequent low temperatures [87]. MT protects wheat seedlings against low-temperature stress by increasing the activities of antioxidant enzymes, maintaining redox balance, reducing ROS accumulation and membrane/DNA damage, boosting osmoprotectant levels, and safeguarding growth and photosynthetic pigments [93]. Similarly, presoaking wheat seeds with 500 μM MT and parental treatment (5 mM) at 12 ± 0.5 °C remarkably increased seed germination by enhancing antioxidant activity, starch metabolism, and chloroplast protection [94]. In waxy maize, seeds presoaked with MT had a higher starch degradation rate under low-temperature stress, which guaranteed the supply of energy and maintained turgor pressure for tissue growth during germination [95]. Overall, MT regulates seed germination by enhancing antioxidant defense and ROS scavenging systems, thereby mitigating stress-induced delays in germination across various plant species [96,97]. In winter wheat, foliar SA application enhances freezing resistance by influencing apoplastic proteins involved in ice nucleation and antifreeze activity [98]. SA pretreatment can activate cellular antioxidant enzymes during chilling stress, thereby increasing resistance to chilling-induced injury [16,99]. Exogenous SA application enhances photosynthetic efficiency, antioxidant activity, and osmoprotectant accumulation, leading to improved growth, biomass, and grain yield under late-spring low-temperature stress [100]. In other species, such as eggplant and tomato, SA also regulates antioxidant-related genes, including catalase (CAT), glutathione reductase (GR), and phenylalanine ammonia-lyase (PAL), while promoting soluble sugar and proline accumulation [101,102]. Importantly, cold priming can increase endogenous SA levels, and SA signaling contributes to cold priming-induced freezing tolerance [20]. Foliar SA application reduces freezing damage and enhances yield-related traits by improving ice nucleation and photosynthesis [98,103]. In addition, NO, applied through sodium nitroprusside (SNP), increased SOD, POD, and CAT activity while reducing H_2_O_2_, O_2_^−^, and MDA in wheat under chilling conditions [104], although excessive NO accumulation can be phytotoxic, inhibiting leaf and root growth and inducing DNA damage [105].

#### 4.2.2. Silicon, Mineral Nutrients, and Nanoparticles

Micronutrients such as Mo and Se, together with Si, contribute significantly to low-temperature tolerance in wheat. Mo application increases freezing tolerance by inducing antioxidant defense systems, regulating genes associated with stress response, and increasing ROS scavenging capacity [106]. Seed soaking in Mo enhanced wheat frost tolerance at 4 °C by upregulating stress-responsive genes and stimulating antioxidant activity [107]. Se applications at 0.5 and 1.0 mg kg^−1^ increased biomass and chlorophyll levels, while higher Se concentrations reduced MDA and superoxide accumulation by strengthening antioxidant capacity [108]. Si is another important component of wheat freezing tolerance, although it is not considered an essential micronutrient for higher plants [109,110]. Si supplementation mitigates cellular damage induced by cold stress through an improved antioxidant defense system, water storage, stabilization of membranes and chlorophyll content, and photosynthetic efficiency [111,112]. Application of 0.1 and 1.0 mM Si improved wheat seedlings at −5 °C by reducing oxidative damage and maintaining leaf water content, particularly in susceptible varieties [111].

### 4.3. Barley: Biostimulant Responses by Category

#### 4.3.1. Phytohormones and Signaling Molecules

In barley, seed soaking with 1 μM MT increased the cold tolerance of hulless barley by reducing growth inhibition and MDA accumulation and restoring the expression rhythms of *HvCCA1* and *HvTOC1* [113]. MT-mediated enhancement of drought priming-induced cold tolerance is also associated with increased ABA, antioxidant activity, and photosynthetic maintenance [88]. However, high concentrations of MT might inhibit plant growth, suggesting that the physiological response to exogenous MT is dose- and development-dependent [114]. In barley, SA improves cold tolerance by upregulating antioxidant defense systems and the transduction of stress signaling pathways [115]; foliar application of SA (0.1 mM) improved SOD, CAT, and POD activities, increased ice nucleation activity, and modified apoplastic protein profiles in both cold-sensitive and cold-tolerant cultivars [85]. SA pretreatment also reduced ROS-related damage and modified H_2_O_2_ accumulation in both types of cultivars [86]. However, enzyme responses vary with cultivar and dose: SA decreased CAT activity in winter wheat under cold conditions, whereas 0.1 mM SA stimulated CAT activity in a tolerant cultivar [116].

#### 4.3.2. Silicon, Mineral Nutrients, and Nanoparticles

In barley, Si application enhances plant growth, membrane stability, and antioxidant defense under chilling and freezing temperatures [117]. Si treatment at 56 mg L^−1^ as Na_2_SiO_3_ enhanced freezing tolerance and improved leaf apoplast composition [118], and Si application has been associated with enhanced stomatal opening, possibly because Si increases proton-pumping activity in both the plasma membranes and tonoplasts [119]. Phosphorus supply also matters at low temperatures: Itoh [120] demonstrated that low temperatures reduced barley growth and soil solution phosphorus (P) concentrations, whereas P application increased dry matter accumulation and P uptake by 60% at low temperatures compared with 17% at high temperatures, mainly because of enhanced diffusion around roots and greater root length. In contrast, nanomaterials that are not biostimulants, such as polystyrene nanoplastics, impaired photosynthesis, carbohydrate metabolism, and antioxidant enzyme activity in barley at 2 °C [121] and altered energy and amino acid metabolism in wheat [122], underscoring that nanoparticle effects are material- and dose-dependent.

#### 4.3.3. Humic Substances and Seaweed and Plant Extracts

Algal extracts from *Sargassum angustifolium* enhanced photosynthetic traits and stress-related metabolites in barley, although biomass improvement was limited [123].

#### 4.3.4. Beneficial Microorganisms

Arbuscular mycorrhizal fungi (AMF) exhibit symbiotic associations with the roots of most terrestrial plant species [124]. Beyond improving nutrient uptake, AMF play a crucial role in enhancing plant tolerance to various abiotic stresses [125,126]. As a mycorrhizal crop, barley benefits from AMF symbiotic associations under both optimal and stress conditions. Hajiboland et al. [127] reported that AMF colonization of 40% by *Glomus versiforme* and 80% by *Rhizophagus irregularis* enhanced growth, survival, and photosynthesis in barley exposed to 5/3 °C, with the magnitude of response depending on the fungal species and cultivar. Moreover, AMF have been shown to increase leaf chlorophyll concentration, photosynthetic efficiency, and antioxidant enzyme activity under low-temperature stress [128,129].

### 4.4. Critical Synthesis of Wheat and Barley Responses to Low-Temperature Stress

**Figure 2:**
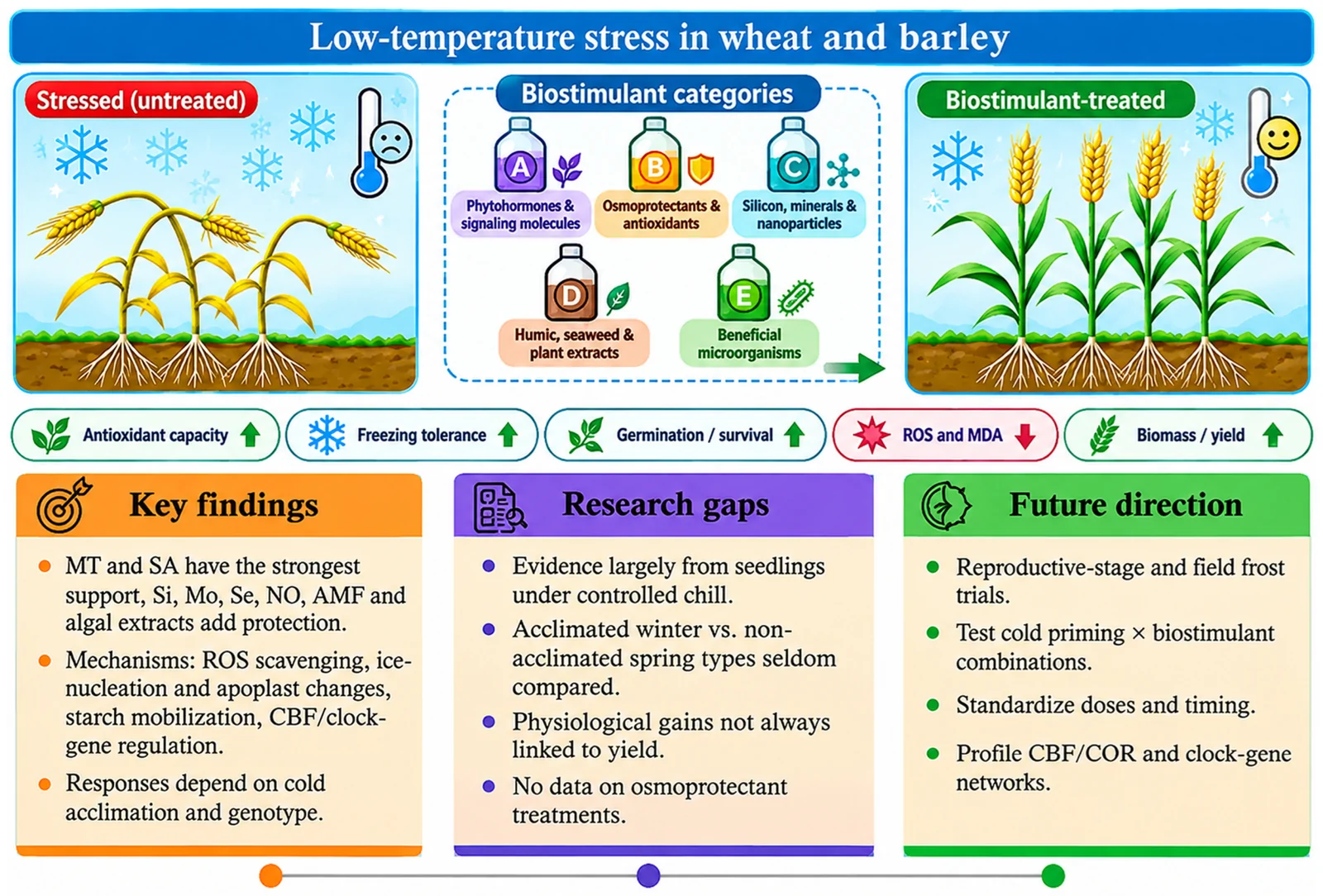
Biostimulant-mediated tolerance to low-temperature stress in wheat and barley. The illustration contrasts untreated plants under cold stress with plants treated with biostimulants of categories A–E and summarizes key findings, research gaps, and future research directions.

Figure 2 and Table 2 summarize the evidence that biostimulants can enhance low-temperature tolerance in wheat and barley through interconnected mechanisms involving ROS detoxification, antioxidant activation, osmotic adjustment, photosynthetic protection, membrane stabilization, and hormonal and stress-memory regulation. MT is involved in antioxidant defense, photosynthetic protection, and stress-memory responses through circadian clock-related genes, whereas SA contributes to antioxidant capacity, apoplastic defense, and freezing tolerance. MT and SA improved cold tolerance in both crops through stronger antioxidant defense. Wheat studies emphasize germination, starch metabolism, and photosynthesis, whereas barley studies emphasize apoplastic and circadian-clock responses. NO, Mo, and Se have been tested only in wheat, and ABA, phosphorus, mycorrhizal fungi, and algal extracts only in barley. Yield effects were rarely assessed, and osmoprotectants, humic substances, and nanoparticles have not been tested in either crop. Available evidence is largely derived from seedling-stage experiments under controlled environments, and responses vary with genotype, cold-acclimation status, developmental stage, treatment concentration, and stress severity; physiological improvements do not always translate into yield benefits. These controlled studies provide testable hypotheses for reproductive-stage and multi-season field validation, comparisons of acclimated and non-acclimated genotypes, standardized application strategies, and mechanistic studies to determine the consistency and agronomic relevance of biostimulant-mediated low-temperature tolerance.

## 5. Biostimulant-Mediated Tolerance to Drought in Wheat and Barley

### 5.1. Effects of Drought and the Role of Genotype

Drought stress is among the most serious environmental limitations to world crop productivity, particularly in wheat-growing regions that are susceptible to water scarcity and climate change [130]. Drought is characterized by its high frequency, long duration, and widespread impact, causing substantial agricultural and economic losses worldwide [131]. In both wheat and barley, severe water deficit negatively affects growth through a decrease in chlorophyll content, photosynthetic efficiency, nutrient uptake, and root development, along with an increase in ROS accumulation resulting in oxidative damage [132,133,134]. At the cellular level, drought induces the production of ROS such as singlet oxygen (^1^O_2_), superoxide (O_2_•^−^), H_2_O_2_, and ·OH [135], which injure DNA, proteins, lipids, and pigments, resulting in oxidative stress, membrane injury, growth inhibition, or cell death [13]. Barley yield losses can reach 50% [136], and barley is very sensitive to severe drought in rain-fed fields, where plant death and complete yield loss can occur [137]. Plant growth regulators (PGRs) have been shown to be effective and eco-friendly strategies for alleviating drought-induced injury [138]. Drought effects are strongly context-dependent. The timing (vegetative vs. terminal), duration, and severity of water deficit, soil water-holding capacity, and concurrent heat determine the damage, and tolerance varies considerably among genotypes. Spring wheat cultivars adapted to tropical and subtropical, water-limited environments, as well as drought-adapted barley genotypes, may maintain growth and yield under water deficit and should not be treated as uniformly susceptible. Genotypic differences were evident in the studies reviewed, for example, the wheat ecotypes Baharband and Telvar [130], cultivar Faisalabad-08 [139], and barley genotypes Giza 123 [140], BARI barley 5 and 7 [141], Rehan [142], and Goharan and Nosrat [143]. The efficiency of Si in improving drought resilience also varied with genotype, stomatal density, and drought intensity and duration [144]. Biostimulants improved performance in both tolerant and susceptible cultivars [145], but the magnitude of the benefit differed among genotypes; the interaction between genotype, environment, and biostimulant therefore needs to be tested explicitly, particularly under field-relevant water regimes.

### 5.2. Wheat: Biostimulant Responses by Category

#### 5.2.1. Phytohormones and Signaling Molecules

MT is one of the most extensively investigated biostimulants for drought tolerance. MT improves seed germination, root growth, water retention, and photosynthetic performance and alleviates oxidative damage by ROS and reactive nitrogen species [146,147]. Plant hormones such as JA and SA also play crucial roles in enhancing drought resilience [148]. Gibberellic acid (GA_3_) is an important growth regulator that relieves stress by acting on antioxidant enzymes to scavenge ROS [149] and encourages cell division and seed germination [150]. ABA priming has been shown to enhance drought tolerance in wheat, maize, and barley by improving water retention, photosynthetic performance, and antioxidant defense [151]. MT improves the drought tolerance of wheat by increasing jasmonic acid, upregulating genes related to stress and metabolism, and reducing photosynthetic and cellular damage [152]. In addition, MT treatment increases the drought tolerance of wheat seedlings by reducing oxidative damage, increasing photosynthesis and water retention, and upregulating antioxidant enzymes and GSH- and AsA-accumulating genes [146]. It also stimulates glutathione (GSH) accumulation and reduces H_2_O_2_ content in wheat and other plants [153,154]. Seed coating with 100–200 μM MT improved germination, antioxidant activity, growth, and yield, while decreasing ROS accumulation under drought stress [155], and MT application at 100 μM improved root morphology, shoot growth, root lifespan, and survival, together with enhanced expression of MT biosynthesis-related genes [156].

In wheat, the application of 100 μM JA and 10 mM SA significantly alleviated drought stress by improving germination, shoot length, water potential, and accumulation of proline and soluble sugars, with JA proving to be more effective [157]. SA pretreatment also reduced lipid peroxidation and improved drought-related growth responses [158]. Foliar application of SA (0.5 mM) improved drought resistance in wheat through improved photosynthetic efficiency, membrane stability, and antioxidant defenses, while Baharband and Telvar were identified as the most drought-resistant ecotypes [130]. Combined application of SA (0.5 mM) and sodium hydrosulfide (NaHS, 0.3 mM) improved RWC, Fv/Fm, and antioxidant defense and reduced H_2_O_2_, MDA, and electrolyte leakage [159]. Hydrogen sulfide (H_2_S) is an important signaling molecule that interacts positively with nitric oxide and SA in regulating defense mechanisms and improving drought tolerance [160], and the synergistic or additive effect of SA and H_2_S would be a promising and sustainable strategy to enhance crop resilience to water deficit [161]. In wheat, GA3 at 200 ppm improved growth, yield, and antioxidant activity under normal and drought conditions, while 200 ppm ABA reduced growth but promoted drought adaptation [162]. ABA is an important regulator of plant development and stress responses, especially under water deficit [163]. Strigolactones also contribute: the synthetic analog GR24 enhanced photosynthesis, water-use efficiency, antioxidant activity, and yield of winter wheat under dehydration, depending on concentration and application method [164], and the effects of strigolactones and their interaction with ABA are tissue-dependent (root vs. shoot) and species-dependent (monocots vs. dicots), indicating complex hormonal crosstalk [165].

#### 5.2.2. Osmoprotectants and Antioxidant Compounds

Combined application of 0.5 mM SA and 10 mM proline increased drought tolerance by enhancing growth, photosynthesis, antioxidant defenses, and grain yield and by decreasing oxidative stress and membrane damage [166].

#### 5.2.3. Silicon, Mineral Nutrients, and Nanoparticles

Zn is an essential micronutrient involved in numerous physiological processes in plants, including protein and carbohydrate synthesis [167]. Among the various NPs, nano-silicon (SiNP) promotes plant growth and photosynthesis under adverse environmental conditions [146]. SiNP application enhances growth, yield, and water status by improving leaf ultrastructure, activating protective and defense mechanisms, and facilitating nutrient uptake, often surpassing the effectiveness of conventional fertilizers [168,169]. The exogenous application of SA and Zn enhanced wheat growth and antioxidant enzyme activity, thereby mitigating drought-induced reductions in physiological and yield-related traits [170,171,172]. The combined application of Fe and Zn nanoparticles significantly enhances wheat growth, photosynthetic efficiency, antioxidant defenses, and yield, while reducing oxidative damage under drought conditions [173]. Nanoparticles can penetrate plant cells through roots and leaves and influence photosynthetic activity, thereby promoting crop growth and biomass production [174], and may improve nutrient uptake and provide protection against abiotic and biotic stresses [175]. ZnONPs promote chlorophyll biosynthesis, whereas FeNPs enhance stomatal conductance, leading to improved gas exchange and CO_2_ assimilation [176,177]. Application of ZnO and FeO nanoparticles alleviated drought-induced damage to wheat growth by enhancing water retention and osmolyte accumulation, with Faisalabad-08 exhibiting the greatest drought resistance [139]; ZnO and Fe_2_O_3_ nanoparticles improved water-use efficiency, osmotic adjustment, antioxidant enzyme activities, and stabilization of photosynthetic pigments in cereal and horticultural plants [178]. ZnONPs can also alleviate mineral deficiency and enhance growth, yield, and quality of wheat under drought stress [179]. On the other hand, high concentrations of ZnONPs cause toxicity and negatively affect growth, chlorophyll content, stomatal conductance, biomass, and yield [180]. Exogenous Si (0.1 mM) alleviates PEG-induced drought stress in wheat by enhancing growth, modulating antioxidant and phytohormone levels, and improving nutrient uptake [181]. SiNPs as seed priming improved growth, yield, and spike and grain parameters, which were associated with enhanced chlorophyll content and antioxidant activity and reduced oxidative stress [182]. Si treatment improves antioxidant defenses by increasing the activities of APX and CAT, thus avoiding cellular damage caused by H_2_O_2_ [183], and Si pretreatment significantly alleviated early-stage drought stress in wheat through improvement of chlorophyll content, water status, antioxidant activity, and photosynthetic efficiency [145].

### 5.3. Barley: Biostimulant Responses by Category

#### 5.3.1. Phytohormones and Signaling Molecules

In barley, exogenous MT improves photosynthesis and hormone balance and stabilizes membranes by alleviating drought-induced oxidative damage [184]. In tobacco, MT alleviates drought-induced leaf senescence by modulating phytohormone networks and the expression of stress-responsive genes, especially upregulation of IAA accumulation combined with downregulated ABA accumulation [185]. An antagonistic effect of ABA and MT on tissue osmotic stress regulation has been reported in various plant species [186]. MT also enhances stress resistance by activating antioxidant enzymes and improving photosynthetic electron transport efficiency [187], promotes lateral root development and improves water uptake, while rhizosphere MT can modify root carbohydrate metabolism and microbial activity [188,189]. In barley, exogenous SA enhances drought tolerance by increasing stomatal conductance, maintaining the efficiency of PSII, and upregulating antioxidant enzyme activity to suppress oxidative injury [190]. SA mitigated drought stress in four barley genotypes by promoting growth, osmolyte accumulation, and ion homeostasis, with Giza 123 showing the highest tolerance [140]; SA application (0.5 mM) also improved water status, osmotic adjustment, and antioxidant defense, with BARI barley 5 outperforming BARI barley 7 [141]. Foliar application of SA, particularly at the end of the tillering stage, effectively mitigated drought stress in barley, with the Rehan variety showing the best performance under drought conditions [142].

GA has been efficiently used to improve seedling growth, germination rate, and yield [191]; the combined effect of SA (1.5 mM) and GA (110 mg L^−1^) enhanced growth, yield components, and grain protein content under optimal irrigation, and all applied regulators improved barley performance under drought stress [192]. ABA is involved in drought adaptation by modulating stomatal closure, gene expression, and plant physiology [193]. In barley, ABA pretreatment improves drought tolerance by inducing early stomatal closure, maintaining photosynthesis and chlorophyll content, and activating stress-responsive gene expression and alternative splicing, ultimately enhancing yield under drought [193]. ABA-induced stomatal closure reduces water loss through transpiration [194], and foliar application of ABA to 30-day-old barley increased chlorophyll content, photosynthesis efficiency, and antioxidant enzyme activity during drought, being more effective than H_2_O_2_ and putrescine [195].

#### 5.3.2. Silicon, Mineral Nutrients, and Nanoparticles

In barley, Si plays a crucial role in enhancing mechanical strength and inducing resistance to abiotic stresses, including salinity, drought, heavy metal toxicity, and temperature extremes [196]. Si seed priming at 2 mM, particularly in the Nimrooz barley cultivar, significantly improved seedling growth, root anatomy, hydraulic conductivity, and nutrient uptake under drought stress induced by 0.8 MPa PEG [197]. The combined application of nano-Si and nano-zeolite with soil conditioners significantly increased growth, yield, nutrient content, antioxidant activity, and economic profitability under drought stress compared with single applications [198]. Potassium dihydrogen phosphate (KH_2_PO_4_) application also enhances photosynthetic efficiency, resulting in improved carbohydrate accumulation under water-deficit stress [199].

#### 5.3.3. Humic Substances and Seaweed and Plant Extracts

In barley, foliar application of humic acid (HA), MT, and KH_2_PO_4_ enhanced growth, antioxidant capacity, and yield under both control and drought conditions, with HA exhibiting the most pronounced effect, particularly in the Jau-17 variety [200]. HA significantly increased chlorophyll a, chlorophyll b, carotenoid, and total chlorophyll content under drought stress compared with untreated controls [201], and enhanced the activity of antioxidant enzymes in both barley varieties [202]. External application of osmoregulators also alleviated drought-induced damage by improving growth, nutrient levels, antioxidant defense, and yield, with actosol showing the greatest efficacy, followed by KH_2_PO_4_, Amino more^®^, and compound fertilizer [201]; the reduction in ROS accumulation observed with actosol may be attributed to enhancement of both enzymatic and nonenzymatic antioxidants [203].

#### 5.3.4. Beneficial Microorganisms

Beneficial microorganisms, particularly *Trichoderma* spp., *Azospirillum* spp., and *Piriformospora indica*, have greatly contributed to enhancing drought tolerance in barley. *Trichoderma* spp. increase antioxidant defenses and can mitigate drought-induced oxidative stress [204,205]; Zhang et al. [206] demonstrated that *Trichoderma* regulated the transcriptional levels of ROS-detoxifying enzyme genes under stress conditions. Seed biopriming with *Trichoderma harzianum* isolates, particularly the gamma-ray mutant (TM), significantly enhanced drought tolerance in barley by improving growth, antioxidant defenses, osmotic regulation, IAA content, and key respiratory enzyme activities, with the cultivar Goharan showing greater resilience than Nosrat [143]. Seed biopriming with beneficial fungi is a recent and non-harmful approach used to enhance seed vigor and plant tolerance against abiotic stress [207]. *Trichoderma* spp. promote plant growth and protection through improved nutrient absorption, organic acid production and chelation by siderophores, antimicrobial metabolite formation, and stress-response phytohormone production [208], and they have been reported to improve drought tolerance in several staple crops, including rice [209], wheat [210], maize [211], and barley [212]. *Azospirillum* spp. alleviate the negative effects of abiotic stresses [213,214]. The simultaneous inoculation of *Piriformospora indica* and *Azospirillum* spp. under drought stress (50% field capacity) improved antioxidant enzymes, decreased oxidative stress, and increased chlorophyll content [215] by modulating ROS and reducing cellular damage [216]. Plant growth-promoting bacteria induce the accumulation of hydrogen peroxide as a signal and increase plant tolerance by upregulating the expression of antioxidant enzyme genes, including *APX*, *SOD*, and *CAT* [217].

### 5.4. Critical Synthesis of Wheat and Barley Responses to Drought Stress

**Figure 3:**
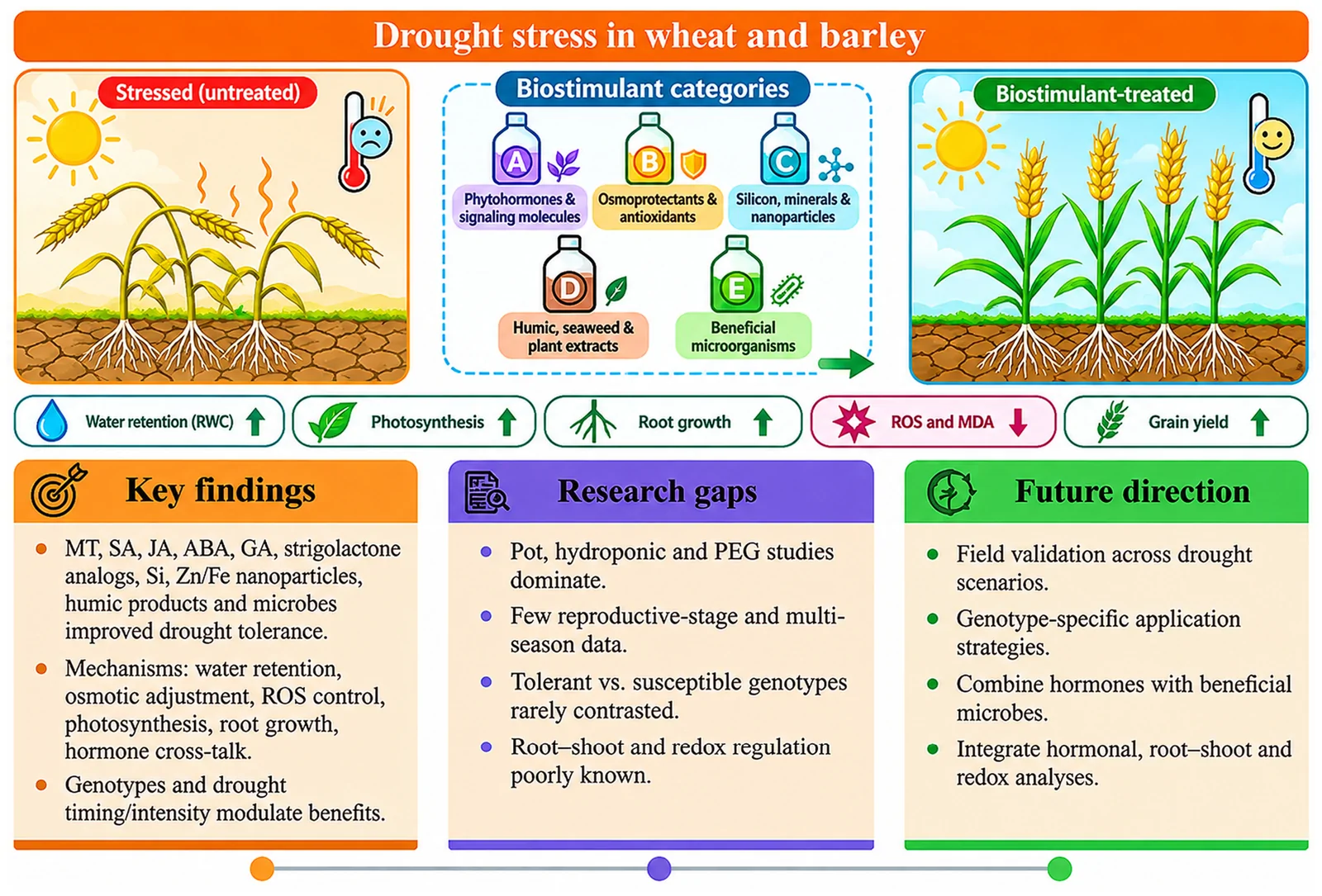
Biostimulant-mediated tolerance to drought stress in wheat and barley. The illustration contrasts untreated plants under water deficit with plants treated with biostimulants of categories A–E and summarizes key findings, research gaps, and future research directions.

Figure 3 and Table 3 summarize the evidence that biostimulants can enhance drought tolerance in wheat and barley through the coordinated regulation of water relations, osmotic adjustment, membrane stability, antioxidant defense, photosynthesis, nutrient acquisition, and hormonal signaling, which supports hormone balance, water-use efficiency, root development, and biomass accumulation. Strigolactones may further contribute to long-term drought adaptation through interactions with ABA signaling. SA and MT are the best-supported biostimulants under drought in both crops, improving water status, photosynthesis, and antioxidant defense, and Si is effective in both. The wheat studies additionally cover JA, ABA, H_2_S, strigolactone analogs, and Zn- and Fe-based nanoparticles, whereas humic products and beneficial microorganisms have been tested only in barley. Yield was reported more often for wheat than for barley. The magnitude of these responses depends on the biostimulant type, concentration, genotype, application strategy, and drought severity. Because most evidence comes from pot, hydroponic, and PEG-based experiments, which allow water deficit to be controlled precisely, the next step is field validation across contrasting water regimes and genotypes, with optimized, genotype-specific application strategies and integrated studies of hormonal, root–shoot, and redox regulation.

## 6. Biostimulant-Mediated Tolerance to Salt Stress in Wheat and Barley

### 6.1. Effects of Salinity and the Role of Genotype

Salinity is a major abiotic stress factor that reduces the productivity of agricultural crops. Approximately 20% of the arable land worldwide is affected by salinity due to anthropogenic activities and climate change [218], and nearly 1.4 billion hectares of land are salt-affected [219], with the affected area increasing by approximately 10% annually [220]. Salinity imposes osmotic stress, ionic imbalance, ion toxicity, and nutritional imbalance, leading to disruption of physiological and metabolic processes and excessive accumulation of ROS; these effects ultimately reduce growth and yield [221,222,223,224]. Salinity-induced nutrient imbalances alone cause approximately a 20% reduction in crop yield [225]. Barley has comparatively greater salt tolerance than many cereals, including wheat, and is a model species for understanding salinity tolerance mechanisms at physiological and molecular levels [226,227], although substantial genotypic variation exists. Salt-tolerant cultivars maintain greater biomass under saline conditions [228,229], and the studies reviewed show genotype-dependent responses, such as Galaxy-2013 in wheat [230], synthetic hexaploid versus bread wheat [231,232], and Kalaycı in barley [233]. Tolerance in cereals is generally attributed to osmotic tolerance, Na^+^ exclusion, and tissue tolerance of accumulated Na^+^ [234].

### 6.2. Wheat: Biostimulant Responses by Category

#### 6.2.1. Phytohormones and Signaling Molecules

SA, a natural phenolic compound, is a growth regulator that plays an important role in regulating physiological processes such as photosynthesis, transpiration, nutrient uptake, chlorophyll biosynthesis, and protein synthesis [235]. In wheat, exogenous SA application enhances antioxidant enzyme activities and the K^+^/Na^+^ ratio while improving plant growth under salinity stress [236]. The exogenous application of SA and its synthetic derivative acetyl-SA improved several morphological traits in wheat shoots, including plant height, tiller number, flag leaf area, spikes per plant, and grains per spike [237,238]. Pretreatment with SA alleviated the inhibitory effects of NaCl-induced salt stress by improving seed germination and root and seedling growth and by limiting oxidative damage through enhanced ROS scavenging [239]. SA also improved K^+^/Na^+^ balance and photosynthetic pigments [240], while foliar application improved growth, yield, chlorophyll, RWC, gas exchange, and antioxidant activity under saline conditions [241,242,243]. SA seed priming improved salt tolerance in wheat seedlings, with the Galaxy-2013 variety showing superior performance [230]. SA induces pre-adaptive responses to salt stress by maintaining membrane integrity and protecting photosynthetic pigments [244]. Exogenous SA application significantly improved salt tolerance in two wheat genotypes by enhancing relative water content, gas exchange, photosynthetic pigments, and yield, while decreasing Na^+^ concentration and the Na^+^/K^+^ ratio [242].

MT application improves seed germination, antioxidant defense, chlorophyll retention, and energy metabolism in wheat under saline conditions [245]. Foliar MT enhances salt tolerance in wheat seedlings by reducing oxidative stress, activating antioxidant systems, and upregulating photoprotection and photosynthetic efficiency [246]. In cotton, MT also regulates endogenous phytohormones, such as ABA and gibberellic acid (GA_3_), thereby improving stress tolerance and enhancing seedling vigor [247]. Exogenous MT (200 μM) increased growth and chlorophyll content of wheat plants under 200 mM NaCl [248]. Similarly, co-applied foliar treatments of 70 μM MT and 75 mg L^−1^ SA improved antioxidant and glyoxalase defense systems, decreased oxidative damage, and enhanced salt tolerance at 6.0–12.0 dS m^−1^ [249], and led to improved nutrient uptake, reduced Na^+^ accumulation, increased root H^+^-pump activity, and strengthened antioxidant defense mechanisms [250]. The increase in H^+^-ATPase activity observed under SA and MT treatments may result from their direct antioxidant action in scavenging ROS or from their indirect role in activating antioxidant enzymes [247,251], and the interactions between MT and SA mainly involve ROS regulation and hormonal balance [252,253].

#### 6.2.2. Osmoprotectants and Antioxidant Compounds

In wheat, AsA contributes to salinity tolerance: seed priming or foliar spray of a combination of AA and SA enhanced photosynthetic pigments, antioxidant activity, and productivity while alleviating oxidative damage under saline conditions [254]. Sequential application of glutathione (1 mM) and MLE (3%) through seed priming and foliar spray enhanced osmotic adjustment, ionic and hormonal balance, photosynthetic performance, and grain yield [255], and exogenous glucose alleviated salt stress during germination and seedling growth by enhancing ion homeostasis, increasing antioxidant enzyme activity, and reducing oxidative damage [256].

#### 6.2.3. Silicon, Mineral Nutrients, and Nanoparticles

Nanotechnology has emerged as a significant advancement in modern agriculture owing to its wide-ranging applications in sustaining agricultural ecosystems [257]. Titanium dioxide nanoparticles (TiO_2_NPs) can induce significant effects on the biochemical and morphophysiological parameters of plants [258]. Selenium (Se) also contributes to alleviating salinity stress by regulating osmotic balance and antioxidant metabolism [259,260]. The plant response to Se largely depends on its concentration [261], as higher levels can be toxic [262]. Se enhances water retention, photosynthesis, and nutrient balance and promotes antioxidant defense systems, diminishing the toxicity of Na^+^ and oxidative damage to membranes [263]. In wheat, foliar application of 40 mg L^−1^ TiO_2_NPs significantly increased plant height, biomass, root and shoot length, leaf number, chlorophyll content, RWC, and membrane stability index (MSI), whereas higher doses (60–80 mg L^−1^) inhibited these traits under salt stress [264]. Similarly, foliar application of TiO_2_NPs (20–40 mg L^−1^) positively affected wheat germination, physiological and biochemical traits, and yield under 100 and 150 mM salinity, while higher concentrations were detrimental [265]. Zn is an essential micronutrient that activates numerous plant enzymes related to sugar metabolism, auxin biosynthesis, protein synthesis, and pollen development [266]. Although modern wheat cultivars have been developed for high grain yields, they often contain low Zn content owing to poor Zn bioavailability in approximately 50% of wheat-growing fields worldwide [267]. Adequate Zn application alleviates salt stress in wheat by reducing sodium accumulation and enhancing potassium uptake, thereby improving iron homeostasis and shoot biomass [268], and foliar application of nano-Zn (up to 50 mg L^−1^) induced osmolyte accumulation, nutrient uptake, and antioxidant enzyme activities [269].

Foliar application of 0.1% green-synthesized SeNPs improved wheat growth, production, and chlorophyll content and decreased oxidative stress (MDA and H_2_O_2_) under salinity [270], and SeNPs (100 mg L^−1^) synthesized by *Bacillus cereus* improved the germination and vegetative growth of wheat under salt stress compared with the untreated control [271]. SeNPs also facilitate the regulation of ion homeostasis and chlorophyll stability through efficient ROS scavenging [272]. Nanomaterials such as ZnO [273] and iron oxide nanoparticles [274] have also been used to promote wheat seed germination, and priming seeds with silver nanoparticles (AgNPs) promoted growth by increasing soluble sugar and proline accumulation and enhancing antioxidant enzyme activities under saline conditions [275]. Moderate N fertilization (86–210 kg N ha^−1^) can alleviate salinity-induced stress in wheat seedlings by improving emergence, growth, and antioxidant activity under low to moderate salinity; however, excessive N fertilizer may be less effective or even harmful under high salinity [276,277]. The detailed mechanisms of salt tolerance conferred by SeNPs in wheat still require further investigation.

#### 6.2.4. Humic Substances and Seaweed and Plant Extracts

Plant-derived extracts show similar beneficial effects under salinity, including moringa leaf extract, maize grain extract, and licorice root extract (the latter tested in common bean) [278,279]. Seed priming with a combination of sorghum water extract (SWE) and benzyl aminopurine significantly boosted wheat salt tolerance by improving growth, chlorophyll, osmolytes, and K^+^ content while reducing Na^+^ accumulation [280], and SWE seed priming was as effective in promoting wheat growth as the commercial growth regulator BAP under optimal conditions [281]. Seaweed extract, particularly from *Ulva rigida*, also improved photosynthetic efficiency and antioxidant enzyme activity under saline conditions [282]. The beneficial effects of plant extracts are largely due to bioactive compounds such as soluble sugars, proteins, and polyphenols, which stimulate the antioxidative defense system [283]. Humic acid (HA) plays a crucial role in enhancing wheat tolerance to salt stress by improving physiological functions, promoting osmoprotectant accumulation, and regulating stress-responsive gene expression, as shown for *P5CS* in perennial ryegrass [284,285], through improved water relations, stomatal conductance, and enhanced antioxidant enzymes such as CAT, POD, and SOD to mitigate ROS [286,287]. HA also protects the photosynthetic apparatus, leading to increased chlorophyll content and photosynthetic rates in salt-sensitive rice [288]. In wheat, HA application significantly improved salt tolerance by improving photosynthesis, antioxidant defense, and the regulation of stress-related gene expression, with synthetic hexaploid genotypes showing greater responsiveness than bread wheat [231,232]. Soil-applied HA increases nitrogen uptake, whereas foliar-applied HA improves the absorption of phosphorus, potassium, magnesium, sodium, copper, and zinc [289]. Combined application of potassium and HA effectively alleviated salinity stress by improving growth, nutrient uptake, osmotic adjustment, and antioxidant enzyme activities in salt-tolerant wheat genotypes [290].

#### 6.2.5. Beneficial Microorganisms

Beneficial microorganisms, including arbuscular mycorrhizal (AM) fungi, also play a vital role in wheat salt tolerance. AM fungi form symbiotic associations with approximately two-thirds of terrestrial plants, including wheat [291]. Giambalvo et al. [292] showed that AM inoculation with *Rhizophagus irregularis* and *Funneliformis mosseae* mitigated salt stress (13 dS m^−1^) in durum wheat, provided that sufficient N (80 mg N pot^−1^) was supplied, enhancing leaf traits, N uptake, and water-use efficiency; AM colonization promotes N uptake and assimilation under salt stress, reducing Na^+^ toxicity and maintaining chlorophyll content.

### 6.3. Barley: Biostimulant Responses by Category

#### 6.3.1. Phytohormones and Signaling Molecules

In barley, foliar application of SA mitigated salt stress by enhancing antioxidant enzyme activity and potassium accumulation, resulting in improved growth and yield under elevated NaCl concentrations [293]; recent studies have highlighted its role in regulating responses to salinity and nutritional disorders by modifying metabolism and antioxidant defense systems [294,295,296]. Seed priming with SA improved germination, increased seedling vigor, and promoted nutrient uptake under saline conditions by reducing oxidative damage and enhancing mineral absorption [297]. SA also regulates phytohormone balance by elevating proline and indole-3-acetic acid (IAA) levels while decreasing ABA, ethylene, and jasmonic acid concentrations, thereby enhancing water retention and stress acclimatization [298]. Foliar application of SA, particularly at 1 mg L^−1^, significantly improved growth, chlorophyll content, and yield traits of barley under saline conditions [299], and SA improved antioxidant activity and phytohormone regulation, with Kalaycı being more tolerant [233]. In barley, exogenous JA enhanced *SNAC1* and *ASMT1* expression, increased MT levels, and strengthened antioxidant defenses under salinity, demonstrating the regulatory role of JA in MT biosynthesis [300]; MT application also induced phenolic acid biosynthesis, increased antioxidant activity, and alleviated oxidative stress in barley seedlings exposed to NaCl [301].

#### 6.3.2. Osmoprotectants and Antioxidant Compounds

In barley, AsA, a major nonenzymatic antioxidant, plays a critical role in protecting plants against salinity by controlling ROS accumulation and preventing oxidative damage [302]. Presoaking barley seeds with 1 mM AsA before exposure to NaCl favored seedling growth and shoot biomass [303], and foliar application of AsA (30–60 mM) effectively alleviated the negative effects of salinity (50–150 mM NaCl) by enhancing growth, photosynthesis, ion uptake, and antioxidant defenses [304]. Combined application of AsA and zinc enhanced salt tolerance, antioxidant capacity, ion homeostasis, yield performance, and grain biofortification [305], and also enhanced soluble protein and free amino acid accumulation [306,307]. Similarly, foliar application of AsA and putrescine (Put) alleviated salinity-induced stress in barley by improving growth, yield, anatomical structures, and physiological performance [308]; exogenous Put improves biometric characteristics and the biochemical composition of various plant species under salt stress [309,310].

#### 6.3.3. Silicon, Mineral Nutrients, and Nanoparticles

Si application has been widely recognized to enhance salinity tolerance in barley. It increases antioxidant enzyme activity and glutathione levels while reducing lipid peroxidation and membrane damage in roots [311], strengthens enzymatic and nonenzymatic antioxidant defenses [312], and improves the water state, photosynthetic ability, and growth of *Hordeum marinum* [313]. Foliar application of Si (0.1–0.2%) enhanced photosynthesis, chlorophyll content, and gas exchange, while reducing DNA methylation under 200 mM NaCl stress in barley plants [314]. Seed priming with CaCl_2_, KNO_3_, and KCl significantly improved barley germination, nutrient uptake (N, P, and Fe), and seedling growth, with CaCl_2_ being more effective in reducing oxidative damage [315]. Zeolite used as a soil amendment under saline irrigation increased potassium, magnesium, and calcium levels in the topsoil and improved barley growth [316].

#### 6.3.4. Humic Substances and Seaweed and Plant Extracts

In barley, SWE increased shoot biomass, improved ion homeostasis, enhanced antioxidant activity, and reduced oxidative damage in salt-sensitive genotypes [317] and enhanced K^+^ uptake and overall plant performance under abiotic stress [318]. Algal extracts, including *Cystoseira mediterranea*, significantly increased germination and growth in barley under saline conditions by improving membrane stability and antioxidant defense mechanisms [319], and algal-derived polyphenols, proteins, polysaccharides, and compatible solutes assist in stress tolerance and antioxidant regulation [320]. In barley, HA and fulvic acid have effective biostimulatory effects: HA pretreatment partially restored seed germination, seedling growth, and leaf morphology under saline conditions [321], and foliar application of HA reduced Na^+^ uptake, had a positive influence on protective metabolites, and decreased oxidative distress in roots and shoots exposed to 100 mM NaCl [322]. Potassium fulvic acid alleviated the combined effects of alkaline salt and freeze–thaw stress by increasing soluble proteins, stabilizing antioxidant enzymes, reducing MDA levels, and enhancing photosynthetic performance [323], and the combination of humic or fulvic acid with reduced phosphorus fertilizer rates significantly improved barley growth, nutrient uptake, and yield under saline conditions [324].

### 6.4. Critical Synthesis of Wheat and Barley Responses to Salt Stress

**Figure 4:**
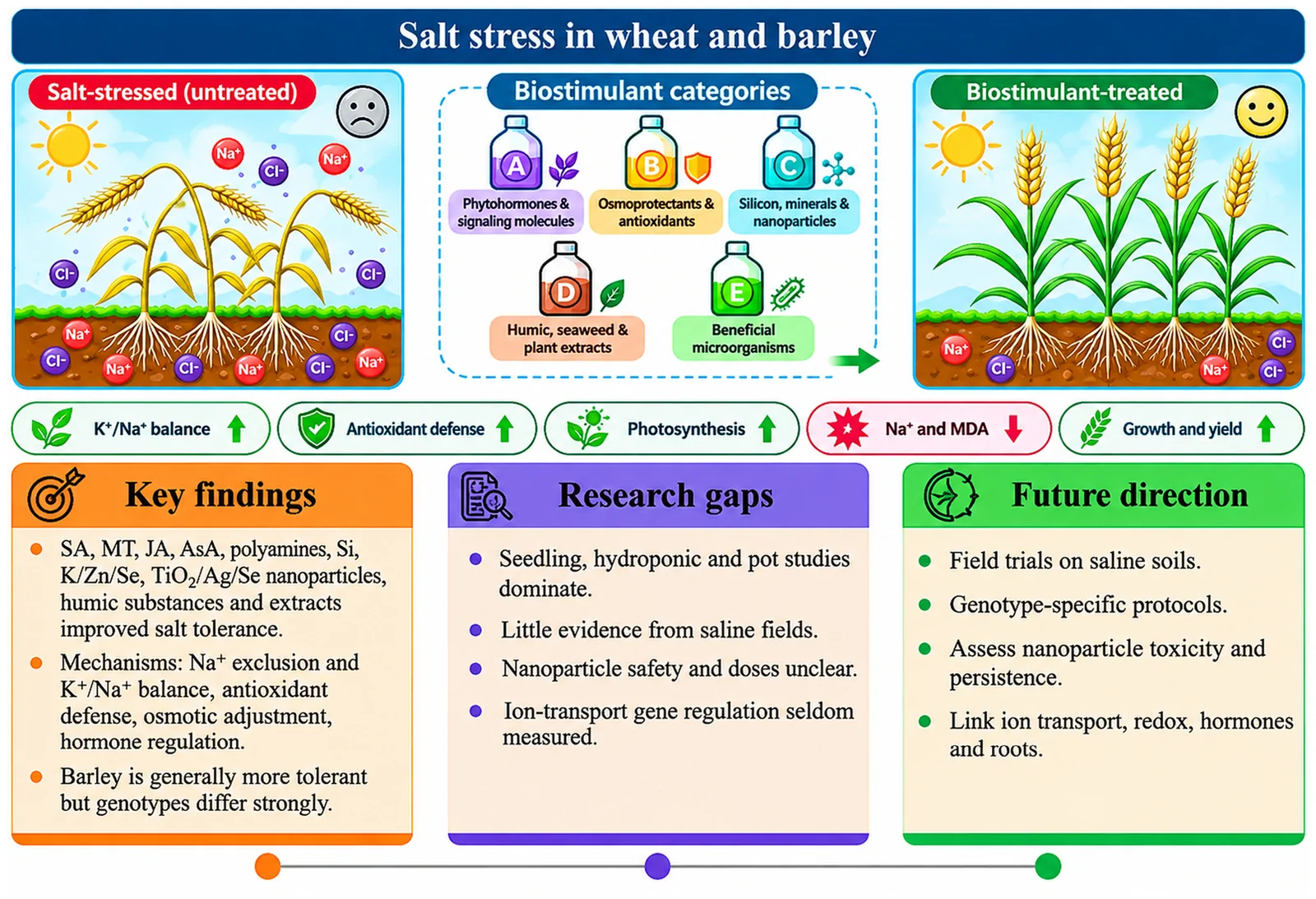
Biostimulant-mediated tolerance to salt stress in wheat and barley. The illustration contrasts untreated plants under salinity with plants treated with biostimulants of categories A–E and summarizes key findings, research gaps, and future research directions.

Figure 4 and Table 4 summarize the evidence that biostimulants can improve salinity tolerance in wheat and barley through the coordinated regulation of ionic homeostasis, antioxidant defense, osmotic adjustment, photosynthesis, membrane stability, and nutrient acquisition. SA and MT act synergistically on hormone regulation (ABA, IAA, and GA) and antioxidant defense, Si reduces Na^+^ uptake and increases water retention, plant-derived extracts may further promote nutrient uptake and stress-responsive metabolism, and MT and JA regulate the expression of their own biosynthesis genes under stress.

SA is the best-supported biostimulant under salinity in both crops, acting mainly through K^+^/Na^+^ balance, osmolyte accumulation, and antioxidant defense, and humic substances, plant and algal extracts, and AsA-type antioxidants are effective in both. MT, nanoparticles (TiO_2_, Zn, Se, and Ag), nitrogen management, and mycorrhizal inoculation have been studied mainly or only in wheat, whereas Si and JA have been studied only in barley. Responses vary with biostimulant type, concentration, genotype, application method, and stress severity. Evidence is largely from seedling, hydroponic, and pot studies that allow salinity to be controlled accurately; these studies provide the basis for field-scale and reproductive-stage evaluation on saline soils, with optimized and genotype-specific application strategies and integrated analysis of ion transport, antioxidant regulation, hormonal signaling, and root responses.

## 7. Biostimulant-Mediated Tolerance to Waterlogging Stress in Wheat and Barley

### 7.1. Effects of Waterlogging and the Role of Genotype

Waterlogging is a significant abiotic stress factor that restricts agricultural production globally, especially in areas with high rainfall and poorly drained soils [325,326]. Extended waterlogging can reduce the yields of major crops, such as wheat and cotton, by approximately 25% and 60%, respectively [327], and barley yield by approximately 20–25% under prolonged waterlogged conditions [328,329]. In upland cereals, waterlogging triggers the formation of adventitious roots and aerenchyma tissue as adaptive responses to oxygen-deficient conditions [330,331]. Under hypoxic conditions, oxygen deprivation severely restricts ATP production and carbohydrate metabolism, resulting in substantial reductions in plant growth and survival [332,333]. The root system is the first organ affected [334]: soil saturation restricts gas exchange and oxygen availability in the root zone, resulting in hypoxia, impaired root function, reduced nutrient uptake, decreased dry matter accumulation, and lower root-to-shoot ratios [334,335,336]. Waterlogging also reduces photosynthesis and carbohydrate accumulation and promotes ROS and MDA accumulation, leading to oxidative damage [337,338]. Flooded plants are exposed to multiple stresses, including hypoxia, reduced carbon dioxide availability, ethylene accumulation, and elevated concentrations of toxic soil elements [339,340]. Under hypoxia, ROS are primarily generated through plasma membrane NADPH oxidase activity, mitochondrial dysfunction, and excessive accumulation of metal ions [341,342]; despite their damaging effects, ROS also function as signaling molecules that regulate acclimation to hypoxia [343,344]. Stress tolerance depends on genotype, soil type and drainage, temperature, duration, and developmental stage; however, most biostimulant studies have been conducted in pots using a single genotype, limiting the generalizability of the findings to field conditions.

### 7.2. Wheat: Biostimulant Responses by Category

#### 7.2.1. Phytohormones and Signaling Molecules

SA is an important plant growth regulator that participates in many physiological processes and stress responses [345,346]. Hydrogen sulfide (H_2_S) and nitric oxide (NO) play important role as signaling molecules in plant adaptation to stress. The exogenous application of H_2_S promotes plant growth by increasing chlorophyll content and enhancing antioxidant capacity, thereby reducing ROS accumulation [347,348]. In wheat under waterlogged conditions, SA encourages adventitious and surface root development independently of the ethylene response, whereas aerenchyma formation depends on ethylene and is associated with upregulation of *ICS* and *PAL* genes [349]. SA also interacts with auxin transport, while excessive SA may inhibit root elongation through altered PIN-mediated auxin transport and redistribution [237]. Moreover, pretreatment with 1-aminocyclopropane-1-carboxylic acid (ACC), an ethylene precursor, improved wheat growth, triggered aerenchyma formation, and increased ethanol fermentation enzyme activity in seminal roots under waterlogging stress [350]. Recently, MT has emerged as a potential bioregulator for improving plant abiotic stress tolerance [351]. In wheat, exogenous MT alleviates waterlogging-induced oxidative damage by upregulating antioxidant enzyme activity and enhancing photosynthetic performance, thereby improving dry matter accumulation, grain weight, and yield [352]; foliar application of MT (100 μM) scavenges ROS, mitigates oxidative stress, and reduces yield loss under waterlogged conditions [353]. However, prolonged waterlogging can still cause photoinhibition, indicating that MT cannot eliminate the effects of severe or extended oxygen deficiency [354]. Notably, foliar-applied MT in non-waterlogged plants was translocated to roots, increasing endogenous root MT levels and promoting shoot and root dry biomass accumulation [355]. MT promotes nitrogen metabolism by inducing enzymes related to nitrogen uptake and incorporation, which improves N, nitrate, and protein contents in stressed plants [356], although endogenous MT concentrations in leaves may not increase because of interactions among other signaling molecules involved in MT biosynthesis and metabolism [357].

Under waterlogging stress, exogenous application of NO and H_2_S reduced oxidative stress and nutrient imbalance in wheat, although NO provided stronger protective effects than H_2_S [358]. Foliar application of sodium nitroprusside (SNP) and sodium hydrosulfide (NaHS) significantly improved plant growth, grain yield, and chlorophyll content under waterlogged conditions, and the enhancement of chlorophyll content might be related to improved absorption of the mineral ions K^+^, Ca^2+^, and Mg^2+^, as reported for NO in tomato under cadmium stress [359]. The synergistic interaction between NO and H_2_S also promotes photosynthesis and yield under stress conditions [360,361].

#### 7.2.2. Silicon, Mineral Nutrients, and Nanoparticles

Nitrogen management is a critical strategy for mitigating waterlogging damage in crops. In wheat, controlled-release nitrogen fertilizer (CRF) increased grain yield and nitrogen-use efficiency by 17–27% compared with single- and split-applied urea at all irrigation regimes, particularly under waterlogging [362]. Post-waterlogging nitrogen fertilizer application increased wheat yield by approximately 20% [363], although nitrogen losses through leaching during waterlogging can reduce N-use efficiency [364]. Extended waterlogging can decrease wheat yield by approximately 1.5% per day of flooding; however, reparative nitrogen fertilization substantially alleviates the negative effects [365]. Post-anthesis nitrogen spraying improved photosynthesis, root activity, and grain yield in winter wheat exposed to waterlogging, with foliar sprays after flowering increasing grain number and weight and yields by up to 12% [366], and ridge planting with optimum NPK fertilization enhanced growth and yield parameters under waterlogged conditions [367].

#### 7.2.3. Humic Substances and Seaweed and Plant Extracts

Plant extract-derived biostimulants have shown promising effects in wheat. Lower concentrations (10–30%) of *Cuscuta reflexa* extract enhanced germination, physiological performance, antioxidative defense, and yield, whereas higher concentrations were toxic [368]. Similarly, sugar beet extract priming protected photosynthetic pigments and improved growth in water-stressed wheat [369].

### 7.3. Barley: Biostimulant Responses by Category

#### 7.3.1. Phytohormones and Signaling Molecules

Plant growth regulators have shown high potential for improving adaptation to hypoxia. Foliar application of naphthaleneacetic acid (1-NAA), an auxin analog, ameliorated waterlogging injury by promoting shoot and root growth, enhancing photosynthetic activity, and facilitating adventitious rooting [370]. Ethephon pretreatment promoted aerenchyma formation in barley roots, delayed leaf chlorosis, and increased root-tip survival under waterlogging, although it reduced root depth [371,372]. Despite the improved rooting response, improvements in shoot biomass accumulation remained limited, suggesting that further optimization of the treatment strategy is necessary. ACC is an ethylene precursor that is converted into ethylene by ACC oxidase [373]; ethephon and ACC treatments activate ethylene-mediated signaling that stimulates aerenchyma development and supports root survival under hypoxia, while their influence on shoot growth remains comparatively limited.

#### 7.3.2. Silicon, Mineral Nutrients, and Nanoparticles

In barley, the application of 90 kg ha^−1^ nitrogen during waterlogging significantly mitigated yield loss by sustaining spike development and limiting accelerated senescence [374], and nitrogen applied immediately after waterlogging improved nitrogen uptake by 33–40% and enhanced plant recovery [375]. Nitrogen fertilizer is often applied during or after waterlogging because it provides a rapid and adjustable means of improving crop nutrition [376], and foliar nutrient application can immediately supply nutrients to aboveground tissues when root uptake capacity is severely impaired by waterlogging, supporting biomass recovery through improved photosynthesis and adventitious rooting [377].

### 7.4. Critical Synthesis of Wheat and Barley Responses to Waterlogging Stress

**Figure 5:**
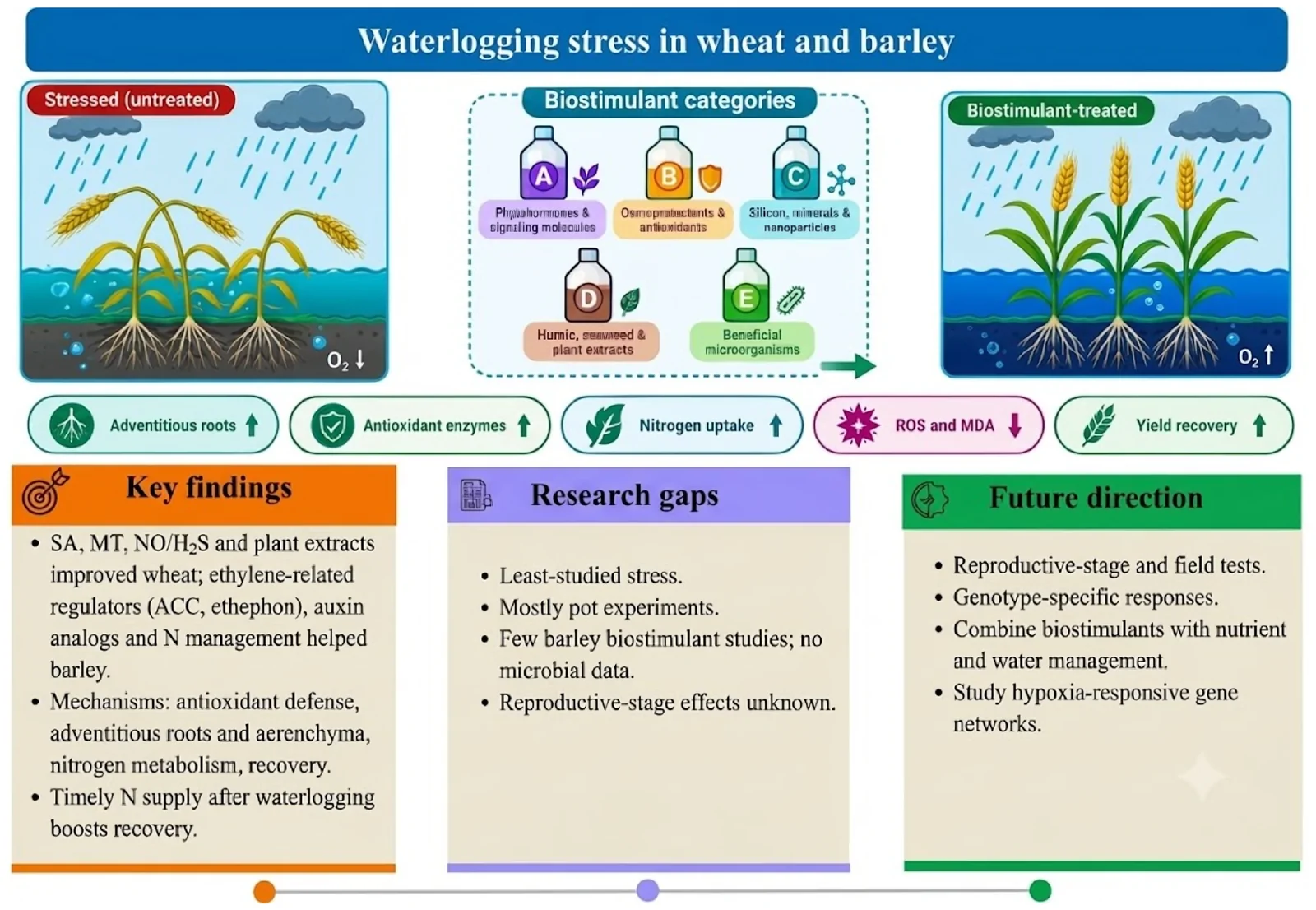
Biostimulant-mediated tolerance to waterlogging stress in wheat and barley. The illustration contrasts untreated plants under waterlogging with plants treated with biostimulants and nutrient or hormone-based treatments (categories A–E) and summarizes key findings, research gaps, and future research directions.

Figure 5 and Table 5 summarize the current evidence that biostimulants can enhance waterlogging tolerance in wheat and barley through complementary physiological, biochemical, and hormonal mechanisms, including improved antioxidant defense, photosynthetic performance, water relations, and hormonal balance, as well as adaptive root responses such as adventitious root and aerenchyma formation. Timely and foliar nitrogen support nutrient acquisition, tillering, and post-waterlogging recovery. Evidence under waterlogging is limited for both crops, and nitrogen management is the only measure tested in both, with benefits in each. The hormonal work in wheat centers on SA, MT, and NO/H_2_S signaling, whereas in barley it centers on auxin and ethylene regulation of root growth and aerenchyma. Other categories have not been tested, and yield data come mainly from wheat. Collectively, these responses help to reduce oxidative damage, maintain growth, and improve plant recovery under hypoxic conditions. The available evidence is relatively limited compared with other abiotic stresses and is derived from controlled experiments, which provide a sound basis for further work on reproductive-stage responses, field validation, genotype-specific effects, and optimized combinations of biostimulants with nutrient and water-management strategies.

## 8. Molecular and Transcriptional Mechanisms Underlying Biostimulant-Induced Tolerance

Most studies summarized above report physiological and biochemical endpoints; however, a growing number provide gene-expression evidence. This section consolidates these findings and distinguishes responses supported by direct gene-expression data from those inferred from physiological outcomes.

### 8.1. Redox and Antioxidant Gene Networks

Biostimulants can modulate genes involved in ROS production and scavenging. MT upregulated *APX*, *MDHAR*, and *DHAR*, together with higher GSH and AsA contents, in drought-stressed wheat [146] and regulated stress-responsive genes under heat [54], and Mo increased antioxidant enzyme activity and *COR15a* expression in frost-exposed wheat [106,107]. HA regulated stress-related genes in saline wheat, with stronger responses in synthetic hexaploid genotypes [231,232]. Among microbial products, *Trichoderma* regulated ROS-detoxifying enzyme genes in salt-stressed wheat [206], and plant growth-promoting rhizobacteria have been reported to upregulate *APX*, *SOD*, and *CAT* in other crops [217]. Heat shock factors are the central regulators of heat shock protein genes in wheat [378], yet biostimulant effects on the Hsf–HSP module have rarely been measured.

### 8.2. Hormone Biosynthesis, Signaling, and Crosstalk

Biostimulants frequently act through the hormonal network. MT increased jasmonic acid and MT biosynthesis gene expression in drought-stressed wheat [152,156] and altered the endogenous phytohormone profile, including IAA and ABA, of drought-stressed barley [184]. In saline barley, JA enhanced *SNAC1* and *ASMT1* expression and MT biosynthesis [300], and SA altered IAA, ABA, ethylene, and JA levels [298]. ABA priming activated stress-responsive genes and alternative splicing in drought-stressed barley [193], and under waterlogging, SA-induced aerenchyma formation was ethylene-dependent and was associated with *ICS* and *PAL* upregulation, whereas adventitious rooting was ethylene-independent [349], and the ethylene precursor ACC increased ethanol fermentation enzyme activity in seminal roots [350]. Crosstalk among SA, JA, ABA, auxin, ethylene, and MT is therefore a major component of the hormonal regulation underlying biostimulant-mediated stress responses.

### 8.3. Stress-Specific and Epigenetic Regulation, and Research Gaps

Cold acclimation in cereals is controlled mainly by CBF/DREB1 transcription factors and cold-regulated genes [41,42]; MT restored the rhythms of the clock genes *HvCCA1* and *HvTOC1* in cold-stressed hulless barley [113], and SA signaling contributed to cold priming-induced freezing tolerance in wheat [20], but direct effects of biostimulants on CBF/COR expression remain untested. Under salinity, Na^+^ exclusion and tissue tolerance, including *HKT1;5*-type transporters in wheat [234,379], underlie tolerance; biostimulants raised K^+^/Na^+^ ratios [236,250] and root H^+^-pump activity [250], but transporter gene expression was seldom analyzed. Si and SiNPs regulated aquaporin genes (*TaPIP1* and *TaNIP2*) in heat-stressed wheat [58]. Beyond transcription, foliar Si (0.1–0.2%) reduced cytosine methylation at CCGG sites in saline barley [314]. Thus, aquaporin, Hsf, CBF, ion-transporter, and epigenetic targets remain promising but underexplored areas for investigating the molecular basis of biostimulant-induced tolerance.

Most molecular studies rely on candidate-gene analyses at a single time point and in a single cultivar. Consequently, causal relationships between biostimulant application, gene regulation, and improved stress tolerance are rarely established. Time-resolved multi-omics analyses across contrasting genotypes, combined with functional validation using mutant, transgenic, or genome-edited lines, are needed to distinguish primary regulatory responses from secondary effects and genotype-specific differences in biostimulant efficacy.

## 9. Conclusions and Future Perspectives

**Figure 6:**
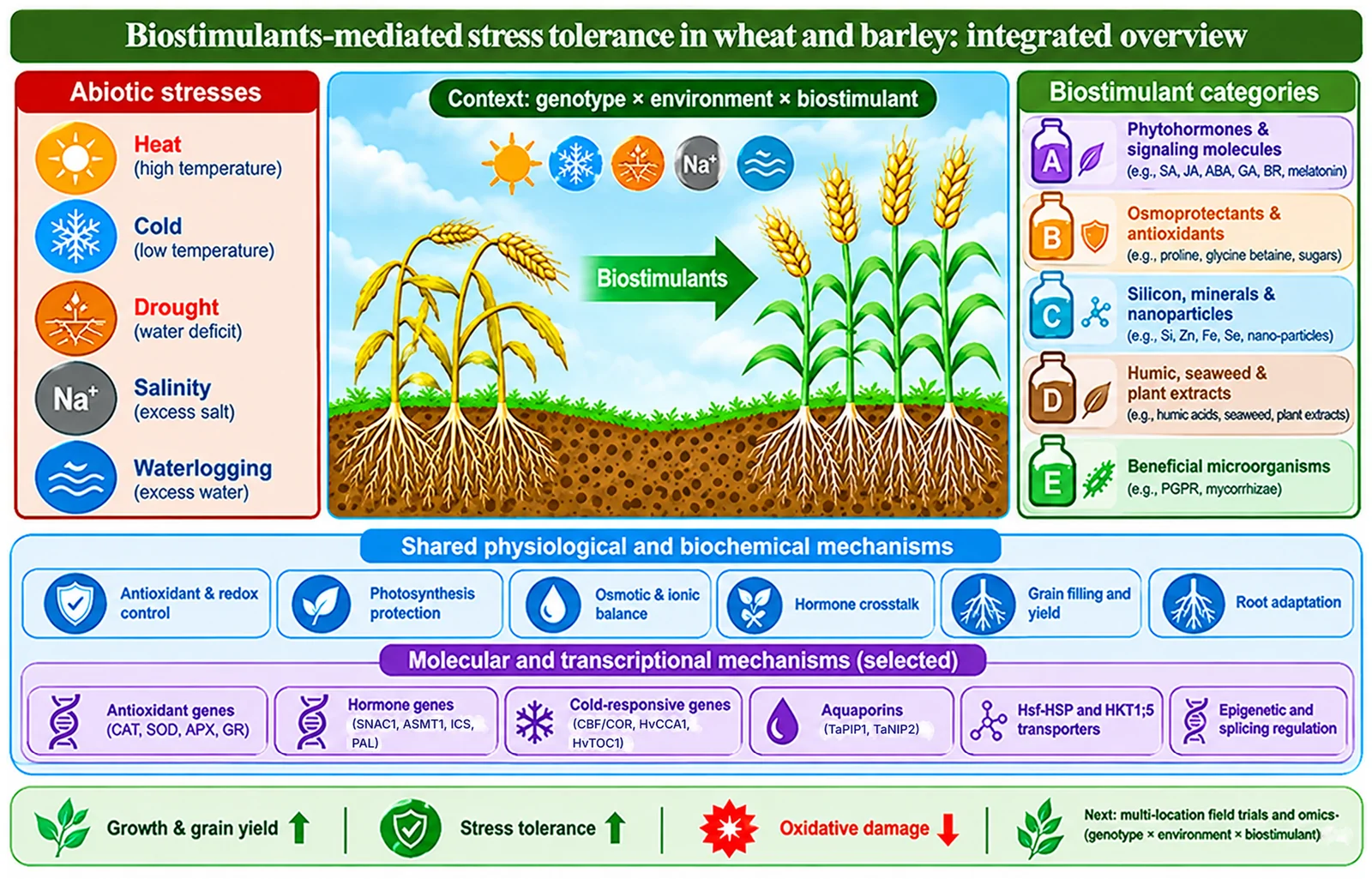
Integrated overview of biostimulant-mediated abiotic stress tolerance in wheat and barley. Five abiotic stresses (heat, cold, drought, salinity, and waterlogging) are alleviated by five categories of biostimulants (A–E) through shared physiological, biochemical, and transcriptional mechanisms, with the outcome modulated by genotype × environment × biostimulant (G × E × B) interactions. Future work should combine multi-location field trials with omics approaches.

Biostimulants enhance growth, yield, and tolerance to abiotic stresses, such as drought, salinity, waterlogging, and high and low temperatures, in wheat and barley. Enhancement of photosynthesis, nutrient uptake, and osmoregulatory and antioxidant defense systems are the primary contributors to these positive effects. Biostimulants also modulate hormonal homeostasis and regulate gene expression, such that plants undergo acclimation at the physiological and metabolic levels, maintaining productivity under environmental stress (Figure 6). Salicylic acid, melatonin, and silicon have the broadest support, whereas evidence for microbial products, osmoprotectants outside barley, and waterlogging is thinner. These findings support that both individual and multi-component biostimulants have the potential to serve as sustainable alternatives to commercial chemical fertilizers and contribute to resource-efficient cereal production systems under climate change. The magnitude of the response depends on genotype and adaptation background, developmental stage, dose, application method, and the intensity, frequency, and cumulative load of the stress.

Most evidence comes from growth chamber, greenhouse, and pot experiments. Such controlled experiments are the appropriate first step, as they allow the stress and environment to be controlled so that active compounds, dose–response relationships, and mechanisms can be established with confidence, and they supply the benchmark against which field performance can be interpreted. The limited number of field studies therefore represents an opportunity as much as a gap, because the controlled-environment findings now provide testable hypotheses for field validation. At the same time, results cannot yet be extrapolated directly to field-grown wheat and barley because of spatial variability in soil properties and changing environmental conditions, technical and economic constraints on large-scale use, variation in product composition, and the lack of standardization. The long-term effects of repeated applications on soil microbiomes, nutrient cycling, and yield stability across diverse agroecosystems also remain largely unexplored.

Future research should focus on: (i) multi-location, multi-season field trials designed to test genotype × environment × biostimulant interactions, including genotypes with contrasting adaptation (tropical and subtropical spring types vs. temperate winter types) and reproductive-stage endpoints; (ii) integration of transcriptomic, proteomic, metabolomic, and epigenomic approaches to define modes of action; (iii) optimization of application methods, dosages, and timing, and synergistic combinations of biostimulants; (iv) standardized formulations and targeted delivery, together with assessment of effects on soil microbiomes; and (v) evaluation of multiple-stress scenarios and economic feasibility. These integrated approaches have the potential to bridge the gap between experimental effectiveness and practical application in the field, thereby supporting the development of climate-smart, resilient cereal production.

## Figures and Tables

**Table 2:** Effects of biostimulant on low-temperature stress tolerance in wheat and barley: application method, growth, yield, and mechanistic insights.

Crop	Stress Level	Biostimulants	Application Methods/ Concentrations	Growth /Yield Effect	Mechanisms	Research Gaps	References
Wheat	5/2 °C	Melatonin (MT)	Foliar (1 mM MT)	None reported	Enhanced redox balance, RuBisCo expression, and osmoprotectants	Lacks field validation	[93]
Wheat	12.0 ± 0.5 °C	Melatonin (MT)	Presoaked (500 μM, 24 h) parental treatment (5 mM MT)	Germination rate	Enhanced antioxidant activity, starch metabolism, and chloroplast protection	Early-stage study; field replication lacking	[94]
Wheat	15/10 °C (15 days), 10/5 °C (30 days) and 5/3 °C (45 days)	Salicylic acid (SA)	Foliar (0.01, 0.1 and 1 mM)	None reported	Increased ice nucleation activity	No yield assessment	[98]
Wheat	5.3–2.4 °C	Salicylic acid (SA)	Foliar (100 μM SA + 0.1% ethanol + 0.05% tween 20)	Grain yield	Enhanced photosynthesis, antioxidant activity, and osmotic adjustment	Short-term experiment	[100]
Wheat	4 °C and 20 °C 8 h	Molybdenum (Mo)	Seed soaking (1% molybdate (NH_4_) 6Mo_7_O_24_·4H_2_O) (0.008 M)	None reported	Upregulating stress-responsive genes, antioxidant enzymes	Limited molecular depth; no field testing	[107]
Wheat	5/2 °C for 3 days	Nitric oxide (NO)	Foliar (0.1 and 1 mM)	None reported	Increased antioxidant enzymes; reduced ROS accumulation	Field data missing	[104]
Wheat	4 °C with a 12/12-h	Selenium (Se)	Soil (0.5, 1, 2, and 3 mg Se kg^−1^ soil)	Biomass	Enhanced chlorophyll and antioxidant capacity; reduced oxidative damage and free radicals	Short-term study; yield response missing	[108]
Wheat	−5 °C	Silicon (Si)	Hydroponic nutrient solution (0.1 and 1.0 mM)	Growth	Improved antioxidant defense, reduced oxidative damage, and maintained leaf water content	Hydroponic only; lacks yield data	[111]
Barley	7/5 °C	Salicylic acid (SA)	Foliar (0.1 mM)	None reported	Enhanced antioxidant enzyme activity, ice nucleation, and apoplastic protein	Physiological focus; yield not measured	[85]
Barley	7/5 °C	Salicylic acid (SA)	Foliar (0.1 mM)	None reported	Reduced oxidative damage; modulated H_2_O_2_ levels, and enhanced antioxidant enzyme activities	Limited replication; no yield data	[86]
Barley	25 °C, 15 °C, 5 °C	Melatonin (MT)	Seed soaking (1 μM)	Growth	Restored circadian rhythms and reduced oxidative stress	Single genotype; short-term study	[113]
Barley	2 ± 0.5 °C and 4/2 °C day/night 30% of pot holding capacity	Melatonin (MT), abscisic acid (ABA), fluridone	Foliar (1 mmol L^−1^ MT, 1.5 mmol L^−1^ ABA, 10 mmol L^−1^ fluridone)	None reported	Enhanced ABA accumulation and antioxidant regulation	Combined hormone effects unclear	[88]
Barley	Gradual decrease 5 °C per day from 25 °C to 5 °C	Silicon (Si)	Soil/substrate application (56 mg L^−1^)	Growth	Enhanced leaf apoplast composition, ROS protection	Needs agronomic validation	[118]
Barley	8 and 20 °C	Phosphorus	Soil (44, 131, 436, and 1310 mg P kg^−1^ soil)	Dry weight	Improved nutrient efficiency	High fertilizer rates; long-term impact unclear	[120]
Barley	2 °C days/ night for 24 h	Polystyrene nanoplastics (PSNPs)	Soil and substrate treatment (2 g L^−1^)	None reported	Reduced photosynthesis, carbohydrate metabolism, and antioxidant enzyme inhibition	Nanoplastic stress; relevance to biostimulants indirect	[121]
Barley	5/3 °C day/night	Mycorrhizal fungi (AMF)	*Glomus versiforme*: 40%, *Rhizophagus irregularis*: 80% root colonization	Growth and survival	Enhanced photosynthesis; species-dependent effects	Specific AMF–cultivar interaction untested	[127]
Barley	5/3 °C day/night	Algal extract	0.05% (*w*/*v*) water extract	None reported	Enhanced photosynthetic traits	Preliminary; no mechanistic detail	[123]

**Table 3:** Effects of biostimulant on drought-stress tolerance in wheat and barley: application method, growth, yield, and mechanistic insights.

Crop	Stress Level	Biostimulants	Application Methods/ Concentrations	Growth/ Yield Effect	Mechanisms	Research Gaps	References
Wheat	PEG-6000 (20%)	Melatonin (MT)	Hydroponic (50, 100, 300, 500, and 1000 μM)	None reported	Alleviated cell membrane, chlorophyll, and photosynthesis	Laboratory scale; no field validation	[152]
Wheat	40 and 60% field capacity	Melatonin (MT)	Soil (500 μM)	Seedling growth	Decreased oxidative damage, increased photosynthetic rate and antioxidant enzyme activity	Limited to seedling stage	[146]
Wheat	37.7 mm rainfall, no irrigation during growth period	Melatonin (MT)	Seed coating (0, 25, 50, 100, and 200 μM)	Germination, growth, and yield	Improved germination and antioxidant activity, and reduced ROS accumulation	Lack of field validation; single cultivar	[155]
Wheat	45–50% relative water content	Melatonin (MT)	Soil drench (100 μM)	Root morphology, shoot growth, root lifespan, and survival	Increased melatonin content and synthesis gene expression	Needs genotype comparisons	[156]
Wheat	35% field capacity	Salicylic acid (SA) and jasmonic acid (JA)	Seed soaking/priming (10 Mm SA and 100 μM JA)	Germination	Enhanced water potential, proline, and soluble sugar	Limited to seedling stage; lack of yield data	[157]
Wheat	PEG-6000 (15%)	Salicylic acid (SA)	Hydroponic pretreatment (0.1, 0.25, 0.5, 0.75, 1.0, 2.0, or 3.0 mM)	Growth, shoot and root dry weights	Reduced lipid peroxidation, and regulated key stress-responsive proteins	Hydroponic only; field application lacking	[158]
Wheat	30% field capacity	Salicylic acid (SA)	Foliar (0.5 mM)	None reported	Increased photosynthesis and antioxidant enzymatic activity	Controlled condition only; lack of yield data	[130]
Wheat	50, 100% field capacity	Salicylic acid (SA), sodium hydrosulfide (NaHS)	Foliar (0.5 mM SA, 0.3 mM NaHS)	None reported	Enhanced proline, RWC, Fv/Fm, chlorophyll, and antioxidants and reduced oxidative damage (H_2_O_2_, MDA, EL)	Limited to field trials	[159]
Wheat	40, 60 and 80% field capacity	Gibberellic acid (GA) and abscisic acid (ABA)	Foliar ABA and GA_3_ at two concentrations (100 ppm and 200 ppm each)	Enhanced growth and yield components	GA_3_ (200 ppm): increased antioxidant enzyme activities	Lacks field validation	[162]
Wheat	None reported	Salicylic acid (SA) and zinc (Zn)	Foliar (500 μM SA and 0.3% Zn)	Growth	Enhanced antioxidant enzyme activities	Lack of field data	[170]
Wheat	35, 55 and 8 0% field capacity	Nanoparticles (NPs), namely iron (Fe), zinc (Zn), and silicon (Si)	Foliar (30 mg L^−1^ for each NPs)	Growth and yield	Enhanced wheat growth, photosynthesis, and antioxidant defense and reduced oxidative damage	Nanotoxicity concerns	[173]
Wheat	20% PEG-6000 and 30–40% of field capacity)	Zinc and iron nanoparticles	Hydroponic, soil and foliar (20, 40, 60 ppm)	Growth and yield	Enhanced water status, FeONPs promoted osmolyte accumulation	Lack of field data	[139]
Wheat	25% water holding capacity (WHC)	Zinc (Zn)	Soil (0, 50, 100, and 150 ppm)	Growth, yield, and grain quality	None reported	Lacks stress physiology data	[179]
Wheat	30% WHC	Silicon nanoparticles (SiNPs)	Seed priming (0, 300, 600, and 900 mg L^−1^)	Growth, yield, and physiological performance	Improved chlorophyll content and antioxidant activity while reducing oxidative stress	Field applicability unknown	[182]
Wheat	38 ± 3% pot field capacity	Silicon (Si)	Presowing/ soil (4 mM)	Growth	Enhanced chlorophyll content, water status, antioxidant activity, and photosynthetic efficiency	Single genotype; no yield evaluation	[145]
Wheat	20% relative soil water content	Silicon (Si)	Soil amendment (1.8 mM Si)	Growth and yield	None reported	Field-scale trials needed	[144]
Wheat	40 ± 5% field capacity	GR24, a synthetic analog of strigolactones	Foliar/residual irrigation (5 and 10 μM)	Yield	Improved photosynthesis and antioxidant enzyme activities	Long-term effects unclear	[164]
Barley	40% of field capacity	Salicylic acid (SA)	Foliar (500 μM SA)	None reported	Photosynthesis, antioxidant defense, and stomatal conductance	Lacks molecular validation	[190]
Barley	Only one irrigation after germination	Salicylic acid (SA), proline	Foliar (10 mM proline and 0.5 mM SA)	Growth, physiological traits, and grain yield	Increased chlorophyll and RWC and decreased EL, MDA, H_2_O_2_, and superoxide (O_2_^·−^)	Field-scale data missing	[166]
Barley	Soil moisture (30, 50, 70 and 90%)	Salicylic acid (SA)	Foliar (0.5 mM SA)	Growth parameter	Enhanced osmolyte accumulation and ion content	Growth only; no yield evaluation	[140]
Barley	7.5% soil moisture content	Salicylic acid (SA)	Foliar (0.5, 1 and 1.5 mM)	None reported	Improved water status, osmotic adjustment, and antioxidant defense	Needs validation across cultivars	[141]
Barley	Severe, moderate stress	Salicylic acid (SA)	Foliar (1 mM)	Growth performance	None reported	Mechanistic pathways unstudied	[142]
Barley	Irrigation levels (80, 100, 120 mm)	Gibberellic acid (GA), salicylic acid (SA), benzyl adenine (BA_6_), superoxide dismutase (SOD)	Foliar (110 mg L^−1^ GA_3_, 1.5 mM SA, 60 mg L^−1^ BA_6_, 110 mg L^−1^ GA_3_ + 1.5 mM SA, and 5 mg L^−1^ SOD)	Growth, yield, and grain quality	None reported	Complex treatment mix; no molecular data	[192]
Barley	30, 70, and 100% field capacity	Melatonin (MT)	Foliar (70 μM)	Growth	Enhanced antioxidant enzymes, membrane stability and photosynthesis and reduced MDA, H_2_O_2_, and electrolyte leakage	Short-term study; no yield data	[184]
Barley	30% of pot water holding capacity	Melatonin (MT)	Soil (2 mmol L^−1^)	None reported	Enhanced root carbohydrate metabolism and antioxidant activity and reduced lipid and inorganic nutrient metabolism	Lacks growth and yield validation	[189]
Barley	50% available water	Nano-silicon, nano-zeolite	Foliar (75 ppm)	Growth, yield	Enhanced nutrient content, biochemical traits and antioxidant activity	Controlled condition only; no field trials	[198]
Barley	0.8 MPa PEG-induced drought stress	Silicon (Si)	Seed priming (1 and 2 mM)	Growth	Improved root anatomy, hydraulic conductivity, and nutrient uptake	Short-term study; no field trials	[197]
Barley	Withholding irrigation after treatment application	Humic acid (HA), potassium dihydrogen phosphate (KH_2_PO_4_) and melatonin (MT)	Foliar (400 mg L^−1^ HA, 20 mg L^−1^ KH_2_PO_4_) and 116 mg L^−1^ MT)	Growth	Enhanced antioxidants, total soluble proteins, total chlorophyll, and mineral contents and reduced MDA and H_2_O_2_	Short-term pot trial	[200]
Barley	One-time surface irrigation after germination	KH_2_PO_4_, Actosol^®^, Amino more^®^, compound fertilizer	Foliar (2–10 L ha^−1^)	Growth and yield	Enhanced antioxidant defenses and nutrient uptake	Multiple product interaction	[201]
Barley	PEG 6000 (5, 10%)	*Trichoderma* *harzianum*	Biopriming (1 × 10^8^ spores mL^−1^)	None reported	Improved growth, antioxidant defenses, osmotic regulation, and IAA content	Limited strain diversity	[143]
Barley	50, 70, and 100% field capacity	*Piriformospora indica*, *Azospirillum* spp.	Soil inoculation (*P. indica* (fungus) and *Azospirillum* spp. (bacteria))	None reported	Enhanced antioxidant enzyme activities, reduced oxidative damage, and improved chlorophyll and proline content	Lack of field replication; growth and yield data are missing	[215]

**Table 4:** Effects of biostimulant on salt stress tolerance in wheat and barley: application method, growth, yield, and mechanistic insights.

Crop	Stress Level	Bio Stimulants	Application Methods/ Concentrations	Growth/ Yield Effect	Mechanisms	Research Gaps	References
Wheat	0, 50, 100, or 200 mM NaCl	Salicylic acid (SA)	Seed treatment (0.5–1 mM)	Germination and plant growth	Enhanced antioxidant defense and reduced membrane damage	Focused on seedlings only	[239]
Wheat	8 ds m^−1^ NaCl	Salicylic acid (SA)	Seed treatment (10^−2^, 10^−4^, and 10^−6^ mol L^−1^ SA)	Growth	Increased K^+^/Na^+^ ratio, improved photosynthetic pigment and osmotic potential	No yield data	[240]
Wheat	500 mg Kg^−1^ of NaCl	Salicylic acid (SA)	Seed priming (100 mg g^−1^)	Seedling growth and stress tolerance	None reported	Lacks field validation or yield-related assessments	[230]
Wheat	80–120 mM NaCl	Salicylic acid (SA)	Foliar (0.5 mM)	Growth	Increased antioxidant enzyme activities and reduced H_2_O_2_	Short-term only	[241]
Wheat	12 dS m^−1^	Salicylic acid (SA)	Foliar (1.00 mM)	Growth, biochemical, and yield parameters	Enhanced chlorophyll content and relative water content	Lack of field data	[243]
Wheat	4–8 dS m^−1^ NaCl	Salicylic acid (SA)	Foliar (0.5, 1 and 1.5 mM)	Growth performance and yield	Enhanced RWC, gas exchange activities and photosynthetic pigments and reduced Na^+^/K^+^ ratio	Single cultivar; no field test	[242]
Wheat	6.2–6.5 ds m^−1^ NaCl	Ascorbic acid (AsA) and salicylic acid (SA)	Seed priming/foliar (0, 100, 200 ppm)	Growth and yield	Improved photosynthetic pigments and antioxidant levels, and reduced lipid peroxidation and oxidative stress	Interaction effects unquantified	[254]
Wheat	200 mM NaCl	Melatonin (MT)	Seed treatment (0 50, 100, 150, 200, 250, and 300 μM)	Germination	Enhanced antioxidant enzyme activity and regulating phytohormones, improving energy metabolism	Only seed treatments; lack of yield data	[245]
Wheat	300 mM NaCl	Melatonin (MT)	Foliar (100 μM)	None reported	Reduced oxidative damage and enhanced antioxidant activity, photosynthesis and photoprotection	Reproductive stage not tested; no field test	[246]
Wheat	200 mM NaCl	Melatonin (MT)	Hydroponic root zone application (200 μM MT)	Growth	Improved chlorophyll content	Hydroponics only; field application missing	[248]
Wheat	6–12 dS m^−1^ NaCl	Melatonin (MT), salicylic acid (SA)	Foliar (70 μM MT, 75 mg L^−1^ SA)	None reported	Reduced Na^+^ accumulation, enhanced root H^+^-pump and improved nutrient uptake and antioxidant defenses	Long-term effects unknown; lack of growth and yield data	[250]
Wheat	9.16 dS m^−1^ NaCl	Moringa leaf extract (MLE), glutathione (GSH)	Seed priming/foliar (3% MLE and 1 mM GSH)	Grain yield	Improved water status and ionic balance and enhanced photosynthesis and hormonal regulation	Short duration; lacks molecular data	[255]
Wheat	4–10 dS m^−1^ NaCl	Benzyl aminopurine (BAP), sorghum water extract (SWE)	Seed priming/ benzyl aminopurine (5 mg L^−1^ BAP, 5% (*v/v*) SWE)	Growth	Enhanced growth, chlorophyll, osmolytes, and K^+^ content while reducing Na^+^ accumulation	Short duration; lacks yield data	[280]
Wheat	34–68 mM NaCl	*Ulva rigida* water extract	Foliar (12.5, 25, 50, and 100%)	Growth	Improved photosynthesis and antioxidant enzyme activity	Non-uniform extract concentrations	[282]
Wheat	200 mM	Glucose	Seed treatments (0, 0.1, 0.5, and 50 mM)	Seed germination and seedling growth	Enhanced ion homeostasis, boosted antioxidant enzyme activity, and reduced oxidative damage	Only seed treatments; lack of yield data	[256]
Wheat	5, 10 and 15 dS m^−1^ NaCl	Humic acid (HA)	Soil (25 kg ha^−1^)	Growth and yield traits	Enhanced photosynthesis, antioxidant activity, and stress-related gene expression	Pod conditions only; lacks field variability	[231]
Wheat	15–60 mM NaCl	Humic acid (HA)	Foliar (0, 0.1, 0.2%) soil (0, 1 and 2 g kg^−1^)	None reported	Improved N uptake and increased P, K, Mg, Na, Cu, and Zn uptake	Lack of yield data	[289]
Wheat	150 mM NaCl	Humic acid (HA)	Soil (2 g kg^−1^)	Growth	Improved photosynthesis and regulated stress-related genes	Controlled conditions only	[232]
Wheat	100 mM NaCl	Humic acid (HA), potassium (K)	Soil (2 g kg^−1^ HA), (5, or 10 mM K)	Growth	Enhanced growth, antioxidant enzyme activities, and nutrient uptake	No field-scale trials	[290]
Wheat	100–150 mM	Titanium dioxide nanoparticles (TiO_2_NPs), *Buddleja asiatica* extract	Foliar (20–80 mg L^−1^ NPs, 20% (*v*/*v*) extract)	Plant height, biomass, leaf number, root and shoot	Improved RWC, MSI, chlorophyll a and b, and total chlorophyll content	No field-scale trials; nanotoxicity not assessed	[264]
Wheat	100–150 mM NaCl	Titanium dioxide nanoparticles (TiO_2_NPs)	Foliar (20, 40, 60, 80 mg L^−1^)	Germination, physiological, biochemical, and yield traits	None reported	Lacks molecular data	[265]
Wheat	0, 5 and 10 dS m^−1^ NaCl	Zinc (Zn)	Basal soil (Zn 0.25 and 5 mg kg^−1^)	Shoot biomass	Enhanced potassium uptake and K/Na balance	Lacks yield data	[268]
Wheat	6.3 dS m^−1^ NaCl	Zn (nanoparticles)	Foliar (0, 20, 50 and 80 mg L^−1^)	Growth	Improved nutrient uptake, osmolyte balance	Needs multi-genotype validation	[269]
Wheat	100 mM NaCl	Selenium (Se)	Soil (5 and 10 μM)	Growth and yield	Enhanced photosynthesis, antioxidant activity, ion balance	No field evaluation	[263]
Wheat	12 dS m^−1^ NaCl	Selenium nanoparticles (SeNPs)	Foliar (0.01, 0.05 and 0.1%)	Growth and yield	Reduced oxidative stress, increased chlorophyll	Safety and accumulation issues	[270]
Wheat	50, 100, and 150 mM NaCl	Selenium nanoparticles (SeNPs)	Seed treatment (50, 75, and 100 mg L^−1^)	Germination rate and growth parameters	None reported	Short-term study; no yield data	[271]
Wheat	150 mM NaCl	Silver nanoparticles (AgNPs)	Seed priming (0, 2, 5, and 10 mM)	Shoot fresh and dry weigh	Increased soluble sugars, proline, and antioxidant defense	No field evaluation	[275]
Wheat	1.4, 2.5, 4.2, and 7.6 dS m^−1^ NaCl	Nitrogen	Soil (86, and 210 kg N ha^−1^)	Growth	Enhanced antioxidant activity	Pod conditions only; field-level validation is needed	[276]
Wheat	13.0 dS m^−1^ NaCl	*Rhizophagus irregularis* and *Funneliformis mosseae, Nitrogen (N)*	Soil (1.5 g pot^−1^ + 80 mg N pot^−1^ AM fungi)	None reported	Enhanced leaf traits, N uptake, and water-use efficiency	Pot-based only; lack of yield data	[292]
Barley	0.67, 5, 10, and 15 dS m^−1^ NaCl	Cycocel (CCC), salicylic acid (SA) and jasmonic acid (JA)	Foliar (19 mM CCC, 1 mM SA, 0.5 mM JA)	Improved growth and yield	Enhanced K^+^ accumulation and antioxidant enzyme activity	Long-term effects on growth, yield and phytochemical content are unknown	[293]
Barley	100 mM or 200 mM NaCl	Salicylic acid (SA)	Seed priming (1250 μM)	Enhanced germination and seedling growth	Reduced oxidative damage and improved essential mineral uptake	Short-term study; no yield data	[297]
Barley	150–300 mM NaCl	Salicylic acid (SA)	Seed priming (0.5 mM)	None reported	Increased proline and IAA, while reducing ABA, ethylene, and CKs—indicating its role in hormonal regulation and osmotic balance	Short-term study; agronomic feasibility unclear	[298]
Barley	120 mM NaC	Salicylic acid (SA)	Foliar (0.5, and 1 mM)	Growth and yield traits	Improved photosynthetic pigments	Limited field testing	[299]
Barley	150 or 300 mM NaCl	Salicylic acid (SA)	Hoagland nutrient solution (0.5 mM)	None reported	Enhanced antioxidant enzyme activities and modulated endogenous phytohormone levels	Hoagland-based only; lack of yield data	[233]
Barley	0, 50, 100 and 150 mM	Ascorbic acid (AsA)	Foliar (30 and 60 mM)	Improved growth and physiological traits	Enhanced photosynthesis, ion uptake, and antioxidant defense mechanisms	None reported; no molecular-level evidence	[304]
Barley	200 mM NaCl	Ascorbic acid (AsA) and zinc (Zn)	Foliar (200 ppm AsA and 0.03% Zn)	Enhanced growth and yield	Enhanced antioxidant activity and ion balance	Long-term effects on growth and yield are unknown	[305]
Barley	9.3 and 14 dS m^−1^ NaCl	Ascorbic acid (AsA) and putrescine (put)	Foliar (100 and 300 ppm AsA) and (100 and 200 ppm Put)	Growth, yield, anatomical features, and physiological traits	None reported	Mechanisms limited; yield not reported	[308]
Barley	120 mmol L^−1^ NaCl	Silicon (Si)	Hydroponic nutrient solution (1.0 mmol L^−1^)	None reported	Enhanced antioxidant enzyme activities and glutathione levels while reducing lipid peroxidation and membrane damage	Short-term hydroponics	[311]
Barley	200 mM NaCl	Silicon (Si)	Hoagland nutrient solution (200 ppm)	Biomass accumulation	Enhanced enzymatic and nonenzymatic antioxidants in barley and reduced ROS and lipid peroxidation	Hoagland-based only; lack of field data	[312]
Barley	150 mM NaCl	Silicon (Si)	Hydroponic nutrient solution (0, 1, or 2 mM)	Growth	Improved growth, photosynthesis, and stress tolerance	Short-term hydroponics; lack of field data	[313]
Barley	200 mM NaCl	Silicon (Si)	Foliar (200 g L^−1^)	None reported	Improved photosynthesis, chlorophyll content, and gas exchange while reducing DNA methylation	Lack of yield data	[314]
Barley	0, 100, and 200 mM NaCl	CaCl_2_, KCl, and KNO_3_	Seed priming (5 mM CaCl_2_ for 20 h), (200 mM KCl for 40 h), and (270 mM KNO_3_ for 40 h)	Germination, nutrient uptake, and seedling growth	Reduced oxidative damage and enhanced salt tolerance	Short-term study; needs field validation	[315]
Barley	10 and 15 dS m^−1^	Seaweed extract (SWE), humic acid (HA), and potassium sulfate K_2_SO_4_	Seed treatment/ Foliar (4 Kg ha^−1^ SWE, 28 L ha^−1^ HA and 300 Kg ha^−1^ K_2_SO_4_)	Shoot biomass	Improved antioxidant activity and reduced oxidative damage	Short-term study; field-level yield and stress tolerance not assessed	[317]
Barley	350 mM NaCl	*Cystoseira mediterranea* extract	Seed treatment (20 and 50%)	Growth	Improved chlorophyll content and membrane stability and reduced oxidative damage	Short-term study; needs field validation	[319]
Barley	150 mM NaCl	Jasmonic acid (JA), diethyldithiocarbamic acid (DIECA)	Foliar/soil pretreatment (100 μM JA, DIECA concentration: 2 mM)	None reported	Enhanced *SNAC1* and *ASMT1* expression, boosted melatonin synthesis and ROS reduction	Molecular pathway partially resolved; lack of yield data	[300]
Barley	60 mM NaCl	Melatonin (MT)	Applied via culture solution (20 μM)	Growth and biomass of barley seedlings	Enhanced phenolic acid biosynthesis, boosted antioxidant defenses, and reduced NaCl-induced damage	Seedling stage only	[301]
Barley	0.25, 0.275, 0.30, 0.325 and 0.35 M NaCl	Humic acid (HA)	Seed pretreatment (HA 28 mg L^−1^)	Germination, seedling growth, leaf anatomy and anatomical traits	None reported	Mechanisms limited; yield not reported	[321]
Barley	100 mM NaCl	Humic acid (HA)	Foliar (6.0 mg L^−1^)	None reported	Reduced sodium uptake, enhanced protective metabolites, and alleviated oxidative stress	Yield traits untested	[322]
Barley	60 mmol L^−1^ NaHCO_3_	Potassium fulvic acid	Soil or root-zone application (0.5 g L^−1^)	None reported	Increased soluble proteins, stabilized antioxidant enzyme activity, reduced MDA levels, and improved photosynthetic performance	Needs field validation	[323]
Barley	saline–sodic clay soil	Humic acid (HA), fulvic acid (FA) and phosphorus fertilizer (P_2_O_5_)	Soil (4.75 kg ha^−1^ HA, 53.57 kg P_2_O_5_ ha^−1^), Foliar (4.75 L ha^−1^ FA)	Growth, yield, and nutrient uptake	None reported	Lacks stress physiology data	[324]

**Table 5:** Effects of biostimulants on waterlogging stress tolerance in wheat and barley: application method, growth, yield, and mechanistic insights.

Crop	Stress Level	Bio Stimulants	Application Methods/ Concentrations	Growth /Yield Effect	Mechanisms	Research Gaps	References
Wheat	2 cm above the soil surface	Salicylic acid (SA), aminoethoxyvinylglycine (AVG), 1-aminobenzotriazole (ABT)	Foliar (1 mM SA, 100 μM ABT and AVG)	None reported	Promoted axile and adventitious root formation	No field validation	[349]
Wheat	2 cm above the soil surface	Melatonin (MT)	Foliar (10 μM)	Dry matter accumulation, grain weight, and overall yield	Reduced oxidative damage and enhanced photosynthesis and antioxidant enzyme activity	Study limited to pot experiments: reproductive-stage effects unknown	[352]
Wheat	2 cm water layer above soil surface (3 weeks)	NaHS (H_2_S donor), SNP (NO donor)	Foliar (H_2_S donor, 1 mM), (NO donor, 250 μM)	None reported	Improved oxidative defense and nutrient regulation, with NO being more effective	Long-term and field-level validation missing	[358]
Wheat	Waterlogging	*Cuscuta reflexa* extract (CRE)	Seed priming (0, 10, 20, 30, 40, and 50%)	Germination, growth, and yield	Enhanced antioxidative defenses and water relations	Short-term study; long-term field data absent	[368]
Wheat	Waterlogging	Fertilizer	Soil basal (46% urea), (16% N, 48% P_2_O_5_) and (60% K_2_O)	Growth, yield, and yield components	None reported	Long-term field data absent, unclear mechanisms	[367]
Wheat	Waterlogging	Nitrogen	Soil (single- or split-applied urea and controlled-release fertilizer (CRF) at 90 kg N ha^−1^)	Grain yield and nitrogen-use efficiency	None reported	Long-term effects unknown; no mechanistic molecular validation	[362]
Wheat	Waterlogging	Nitrogen	Soil (50, 100, or 150 kg N ha^−1^)	Yield	None reported	Physiological mechanisms were limited	[365]
Wheat	0.5–1 cm water layer for 6 days	Nitrogen	Foliar (1% urea solution, 10 kg N ha^−1^)	Key physiological and yield traits	None reported	Mechanistic pathways unstudied	[366]
Barley	Waterlogging	Nitrogenous fertilizer	Foliar (20, 30, 90 kg·ha^−1^) soil fertilizer (45, 90 kg ha^−1^)	Tiller numbers, plant height, and canopy greenness, reduced yield loss (90 kg ha^−1^)	None reported	No molecular analysis; short-term experiment	[374]
Barley	Waterlogging maintained 2 weeks	1-NAA (1-naphthaleneacetic acid)	Foliar (5 μg 1-NAA per plant)	Shoot and root growth	Enhanced chlorophyll content, photosynthesis, nitrogen and potassium uptake, and auxin accumulation	Not evaluated under field conditions; no biochemical validation	[370]
Barley	Waterlogging	Ethephon	Hydroponic pretreatment (0, 0.1, 1, 37.5, 70, or 150 μM)	Root aerenchyma formation and increased root-tip survival	None reported	Mechanistic pathways unstudied; lacks growth/yield validation	[371]

## Data Availability

No new data were created or analyzed in this study. Data sharing is not applicable to this article.

## References

[1] Lau S.E., Lim L.W.T., Hamdan M.F., Chan C., Saidi N.B., Ong-Abdullah J., Tan B.C. (2025). Enhancing plant resilience to abiotic stress: The power of biostimulants. Phyton Int. J. Exp. Bot..

[2] Xiong W., Reynolds M., Xu Y. (2022). Climate change challenges plant breeding. Curr. Opin. Plant Biol..

[3] FAO (2009). How to Feed the World in 2050.

[4] Avni R., Nave M., Barad O., Baruch K., Twardziok S.O., Gundlach H., Hale I., Mascher M., Spannagl M., Wiebe K. (2017). Wild emmer genome architecture and diversity elucidate wheat evolution and domestication. Science.

[5] Pourkheirandish M., Hensel G., Kilian B., Senthil N., Chen G., Sameri M., Azhaguvel P., Sakuma S., Dhanagond S., Sharma R. (2015). Evolution of the grain dispersal system in barley. Cell.

[6] Aprile A., Sabella E., Francia E., Milc J., Ronga D., Pecchioni N., Ferrari E., Luvisi A., Vergine M., De Bellis L. (2019). Combined effect of cadmium and lead on durum wheat. Int. J. Mol. Sci..

[7] Xie W., Xiong W., Pan J., Ali T., Cui Q., Guan D., Meng J., Mueller N.D., Lin E., Davis S.J. (2018). Decreases in global beer supply due to extreme drought and heat. Nat. Plants.

[8] Panta S., Flowers T.J., Lane P., Doyle R., Haros G., Shabala S. (2014). Halophyte agriculture: Success stories. Environ. Exp. Bot..

[9] Thilert W. (2006). A unique product: The story of the imidacloprid stress shield. Pflanzenschutz-Nachr. Bayer..

[10] Pugnaire F.I., Morillo J.A., Peñuelas J., Reich P.B., Bardgett R.D., Gaxiola A., Van der Putten W.H. (2019). Climate change effects on plant–soil feedbacks and consequences for biodiversity and functioning of terrestrial ecosystems. Sci. Adv..

[11] O’Neill B.C., Oppenheimer M., Warren R., Hallegatte S., Kopp R.E., Pörtner H.O., Yohe G. (2017). IPCC reasons for concern regarding climate change risks. Nat. Clim. Change.

[12] Mittler R. (2006). Abiotic stress, the field environment and stress combination. Trends Plant Sci..

[13] Gill S.S., Tuteja N. (2010). Reactive oxygen species and antioxidant machinery in abiotic stress tolerance in crop plants. Plant Physiol. Biochem..

[14] Mutlu S., Atici Ö., Esim N., Mete E. (2011). Essential oils of catmint (*Nepeta meyeri*) induce oxidative stress in early seedlings of various weed species. Acta Physiol. Plant..

[15] Sachdev S., Ansari S.A., Ansari M.I., Fujita M., Hasanuzzaman M. (2021). Abiotic stress and reactive oxygen species: Generation, signaling, and defense mechanisms. Antioxidants.

[16] Kang G., Wang C., Sun G., Wang Z. (2003). Salicylic acid changes activities of H_2_O_2_-metabolizing enzymes and increases the chilling tolerance of banana seedlings. Environ. Exp. Bot..

[17] Hasanuzzaman M., Bhuyan M.H.M.B., Zulfiqar F., Raza A., Mohsin S.M., Mahmud J.A., Fujita M., Fotopoulos V. (2020). Reactive oxygen species and antioxidant defense in plants under abiotic stress: Revisiting the crucial role of a universal defense regulator. Antioxidants.

[18] Liu Y., Jiang H., Zhao Z., An L. (2010). Nitric oxide synthase-like activity-dependent nitric oxide production protects against chilling-induced oxidative damage in *Chorispora bungeana* suspension-cultured cells. Plant Physiol. Biochem..

[19] Mahajan S., Tuteja N. (2005). Cold, salinity and drought stresses: An overview. Arch. Biochem. Biophys..

[20] Wang W., Wang X., Zhang X., Wang Y., Huo Z., Huang M., Cai J., Zhou Q., Jiang D. (2021). Involvement of salicylic acid in cold priming-induced freezing tolerance in wheat plants. Plant Growth Regul..

[21] Zandalinas S.I., Mittler R. (2022). Plant responses to multifactorial stress combination. New Phytol..

[22] Farooq M., Bramley H., Palta J.A., Siddique K.H.M. (2011). Heat stress in wheat during reproductive and grain-filling phases. Crit. Rev. Plant Sci..

[23] Mandal S., Anand U., López-Bucio J., Kumar M., Lal M.K., Tiwari R.K., Dey A. (2023). Biostimulants and environmental stress mitigation in crops: A novel and emerging approach for agricultural sustainability under climate change. Environ. Res..

[24] Moreno-Hernández J.M., Benítez-García I., Mazorra-Manzano M.A., Ramírez-Suárez J.C., Sánchez E. (2020). Strategies for production, characterization and application of protein-based biostimulants in agriculture: A review. Chil. J. Agric. Res..

[25] Yakhin O.I., Lubyanov A.A., Yakhin I.A., Brown P.H. (2017). Biostimulants in plant science: A global perspective. Front. Plant Sci..

[26] Franzoni G., Bulgari R., Ferrante A. (2021). Maceration time affects the efficacy of borage extracts as potential biostimulant on rocket salad. Agronomy.

[27] Drobek M., Frąc M., Cybulska J. (2019). Plant biostimulants: Importance for improving the quality and yield of horticultural crops and enhancing plant tolerance to abiotic stress—A review. Agronomy.

[28] Xu L., Geelen D. (2018). Developing biostimulants from agro-food and industrial by-products. Front. Plant Sci..

[29] Sible C.N., Seebauer J.R., Below F.E. (2021). Plant biostimulants: A categorical review, their implications for row crop production, and relation to soil health indicators. Agronomy.

[30] Singh I., Anand K.V., Solomon S., Shukla S.K., Rai R., Zodape S.T., Ghosh A. (2018). Can we mitigate climate change using seaweed-based biostimulants? A case study with sugarcane cultivation in India. J. Clean. Prod..

[31] Lau S.E., Teo W.F.A., Teoh E.Y., Tan B.C. (2022). Microbiome engineering and plant biostimulants for sustainable crop improvement and mitigation of biotic and abiotic stresses. Discov. Food.

[32] Rouphael Y., Colla G. (2020). Editorial: Biostimulants in agriculture. Front. Plant Sci..

[33] Ali O., Ramsubhag A., Jayaraman J. (2021). Biostimulant properties of seaweed extracts in plants: Implications towards sustainable crop production. Plants.

[34] Brown P., Saa S. (2015). Biostimulants in agriculture. Front. Plant Sci..

[35] Franzoni G., Cocetta G., Prinsi B., Ferrante A., Espen L. (2022). Biostimulants on crops: Their impact under abiotic stress conditions. Horticulturae.

[36] Radzikowska-Kujawska D., John P., Piechota T., Nowicki M., Kowalczewski P.Ł. (2023). Response of winter wheat (*Triticum aestivum* L.) to selected biostimulants under drought conditions. Agriculture.

[37] Shaheen A., Yang Y., Shah G., Bhatti H.A., Li Z., Li H. (2026). From stress to success: Biostimulants and nanotechnology-driven strategies to enhance wheat resilience under abiotic stress. Plant Stress.

[38] Elbasyoni I.S. (2018). Performance and stability of commercial wheat cultivars under terminal heat stress. Agronomy.

[39] Cossani C.M., Reynolds M.P. (2012). Physiological traits for improving heat tolerance in wheat. Plant Physiol..

[40] Ul-Allah S., Azeem A., Sher A., Ijaz M., Sattar A., Saleem M.A., Bibi M., Abbas N., Hussain M. (2021). Assessment of genetic variability and direct–indirect contribution of post-anthesis traits to grain yield in bread wheat (*Triticum aestivum*) at different sowing dates. Int. J. Agric. Biol..

[41] Thomashow M.F. (1999). Plant cold acclimation: Freezing tolerance genes and regulatory mechanisms. Annu. Rev. Plant Physiol. Plant Mol. Biol..

[42] Vágújfalvi A., Galiba G., Cattivelli L., Dubcovsky J. (2003). The cold-regulated transcriptional activator Cbf3 is linked to the frost-tolerance locus Fr-A2 on wheat chromosome 5A. Mol. Genet. Genom..

[43] Zahra M., Rasul S., Saeed F., Rasool L., Manzoor H. (2022). Exogenous application of salicylic acid mitigates heat-induced oxidative stress in barley (*Hordeum vulgare* L.). Pak. J. Biochem. Biotechnol..

[44] Wahid A., Shabbir A. (2005). Induction of heat stress tolerance in barley seedlings by pre-sowing seed treatment with glycinebetaine. Plant Growth Regul..

[45] Mittler R., Finka A., Goloubinoff P. (2012). How do plants feel the heat?. Trends Biochem. Sci..

[46] Khan M.I.R., Asgher M., Khan N.A. (2013). Rising temperature in the changing environment: A serious threat to plants. Clim. Change Environ. Sustain..

[47] Shah F., Nie L., Cui K., Shah T., Wu W., Chen C. (2014). Rice grain yield and component responses to near 2 °C warming. Field Crops Res..

[48] Kousar R., Qureshi R., Din J.U., Munir M. (2018). Salicylic acid mediated heat stress tolerance in selected bread wheat genotypes of Pakistan. Pak. J. Bot..

[49] Fan Y., Lv Z., Li Y., Qin B., Song Q., Ma L., Wu Q., Zhang W., Ma S., Ma C. (2022). Salicylic acid reduces wheat yield loss caused by high temperature stress by enhancing photosynthetic performance of the flag leaves. Agronomy.

[50] Asthir B., Kaur S., Mann S.K. (2009). Effect of salicylic and abscisic acid administered through detached tillers on antioxidant system in developing wheat grains under heat stress. Acta Physiol. Plant..

[51] Wang X., Cai J., Jiang D., Liu F., Dai T., Cao W. (2011). Pre-anthesis high-temperature acclimation alleviates damage to the flag leaf caused by post-anthesis heat stress in wheat. J. Plant Physiol..

[52] Zheng J.J., Zhao H.J., Hu W.W., Zhao X.J., Zhao Y.D. (2013). Effect of heat and high irradiation stress on Deg1 protease and D1 protein in wheat chloroplasts and regulating role of salicylic acid. Acta Ecol. Sin..

[53] Rasheed F., Mir I.R., Sehar Z., Fatma M., Gautam H., Khan S., Anjum N.A., Masood A., Sofo A., Khan N.A. (2022). Nitric oxide and salicylic acid regulate glutathione and ethylene production to enhance heat stress acclimation in wheat involving sulfur assimilation. Plants.

[54] Buttar Z.A., Wu S.N., Arnao M.B., Wang C., Ullah I., Wang C. (2020). Melatonin suppresses heat stress-induced damage in wheat seedlings by modulating antioxidant machinery. Plants.

[55] Sehar Z., Fatma M., Khan S., Mir I.R., Abdi G., Khan N.A. (2023). Melatonin influences methyl jasmonate-induced protection of photosynthetic activity in wheat against heat stress by regulating ethylene-synthesis genes and antioxidant metabolism. Sci. Rep..

[56] Alzahrani Y., Kuşvuran A., Alharby H.F., Kuşvuran S., Rady M.M. (2018). The defensive role of silicon in wheat against stress conditions induced by drought, salinity or cadmium. Ecotoxicol. Environ. Saf..

[57] Mustafa T., Sattar A., Sher A., Ul-Allah S., Ijaz M., Irfan M., Cheema M. (2021). Exogenous application of silicon improves the performance of wheat under terminal heat stress by triggering physio-biochemical mechanisms. Sci. Rep..

[58] Younis A.A., Khattab H., Emam M.M. (2020). Impacts of silicon and silicon nanoparticles on leaf ultrastructure and TaPIP1 and TaNIP2 gene expression in heat-stressed wheat seedlings. Biol. Plant..

[59] Hussain M., Khan T.A., Yusuf M., Fariduddin Q. (2019). Silicon-mediated role of 24-epibrassinolide in wheat under high-temperature stress. Environ. Sci. Pollut. Res..

[60] Hassan N.S., Salah El Din T.A., Hendawey M.H., Borai I.H., Mahdi A.A. (2018). Magnetite and zinc oxide nanoparticles alleviated heat stress in wheat plants. Curr. Nanosci..

[61] Thakur B., Asthir B., Kaur G., Kalia A., Sharma A. (2022). Zinc oxide and titanium dioxide nanoparticles influence heat stress tolerance mediated by antioxidant defense system in wheat. Cereal Res. Commun..

[62] Iqbal M., Raja N.I., Mashwani Z.U.R., Wattoo F.H., Hussain M., Ejaz M. (2018). Assessment of AgNP exposure on physiological and biochemical changes and antioxidative defense system in wheat (*Triticum aestivum* L.) under heat stress. IET Nanobiotechnol..

[63] Burman U., Saini M., Kumar P. (2013). Effect of zinc oxide nanoparticles on growth and antioxidant system of chickpea seedlings. Toxicol. Environ. Chem..

[64] Afzal A., Akram M.W., Rehman H.U., Rashid S., Basra S.M.A. (2020). Moringa leaf and sorghum water extracts and salicylic acid alleviate heat stress in wheat. S. Afr. J. Bot..

[65] Foidl N., Makkar H.P.S., Becker K. The potential of *Moringa oleifera* for agricultural and industrial uses. Proceedings of the International Workshop “What Development Potential for Moringa Products?”.

[66] Ananieva E.A., Alexieva V.S., Popova L.P. (2002). Treatment with salicylic acid decreases the effects of paraquat on photosynthesis. J. Plant Physiol..

[67] Tabur S., Yilmaz-Ergün Ş.B., Özme S. (2024). Role of ten different exogenous plant growth promoters in regulating cytotoxic and genotoxic processes in barley exposed to high temperature stress. Caryologia.

[68] Tütünoğlu B., Aksoy Ö., Özbek R., Uçkan F. (2019). Effects of gibberellic acid on Allium cepa root tip meristematic cells. Biol. Plant..

[69] Mansour M.M., Kamel E.A.R. (2005). Interactive effect of heavy metals and gibberellic acid on mitotic activity and metabolic changes of *Vicia faba* L. plants. Cytologia.

[70] Kartal G., Temel A., Arican E., Gözükırmızı N. (2009). Effects of brassinosteroids on barley root growth, antioxidant system and cell division. Plant Growth Regul..

[71] Tabur S., Demir K. (2008). Effects of triacontanol pretreatment on mitotic index and chromosome abnormalities under salt stress. J. Biol. Sci. Res..

[72] Giri G.S., Schillinger W.F. (2003). Seed priming winter wheat for germination, emergence and yield. Crop Sci..

[73] Afzal I., Basra S.M.A., Farooq M., Nawaz A. (2006). Alleviation of salinity stress in spring wheat by hormonal priming with abscisic acid, salicylic acid and ascorbic acid. Int. J. Agric. Biol..

[74] Oukarroum A., El Madidi S., Strasser R.J. (2012). Exogenous glycine betaine and proline play a protective role in heat-stressed barley leaves (*Hordeum vulgare* L.): A chlorophyll a fluorescence study. Plant Biosyst..

[75] Zhou M.X., Zhang G., Li C. (2009). Barley production and consumption. Genetics and Improvement of Barley Malt Quality.

[76] Naz R., Gul F., Zahoor S., Nosheen A., Yasmin H., Keyani R., Shahid M., Hassan M.N., Siddiqui M.H., Batool S. (2022). Interactive effects of hydrogen sulfide and silicon enhance drought and heat tolerance by modulating hormones, antioxidant defense enzymes and redox status in barley (*Hordeum vulgare* L.). Plant Biol..

[77] Hussain I., Parveen A., Rasheed R., Ashraf M.A., Ibrahim M., Riaz S., Ahmad A., Iqbal M. (2019). Exogenous silicon modulates growth, physio-chemicals and antioxidants in barley (*Hordeum vulgare* L.) exposed to different temperature regimes. Silicon.

[78] Zhang Q., Zhang J.Z., Chow W.S., Sun L.L., Chen J.W., Chen Y.J., Peng C.L. (2011). Influence of low temperature on photosynthesis and antioxidant enzymes in sensitive banana and tolerant plantain (*Musa* sp.) cultivars. Photosynthetica.

[79] Frederiks T.M., Christopher J.T., Sutherland M.W., Borrell A.K. (2015). Post-head-emergence frost in wheat and barley: Defining the problem, assessing the damage and identifying resistance. J. Exp. Bot..

[80] Shah T., Latif S., Khan H., Munsif F., Nie L. (2019). Ascorbic acid priming enhances seed germination and seedling growth of winter wheat under low temperature due to late sowing in Pakistan. Agronomy.

[81] Bajwa V.S., Shukla M.R., Sherif S.M., Murch S.J., Saxena P.K. (2013). Role of melatonin in alleviating cold stress in Arabidopsis thaliana. J. Pineal Res..

[82] Mutlu S., Atici O., Nalbantoglu B. (2009). Effects of salicylic acid and salinity on apoplastic antioxidant enzymes in two wheat cultivars differing in salt tolerance. Biol. Plant..

[83] Rout N.P., Shaw B.P. (2001). Salt tolerance in aquatic macrophytes: Possible involvement of antioxidative enzymes. Plant Sci..

[84] Xu S., Li J.L., Zhang X.Q., Wei H., Cui L.J. (2006). Effects of heat acclimation pretreatment on membrane lipid peroxidation, antioxidant metabolites and chloroplast ultrastructure in two cool-season turfgrass species under heat stress. Environ. Exp. Bot..

[85] Mutlu S., Karadağoğlu Ö., Atici Ö., Nalbantoğlu B. (2013). Protective role of salicylic acid applied before cold stress on antioxidative system and protein patterns in barley apoplast. Biol. Plant..

[86] Mutlu S., Atıcı Ö., Nalbantoğlu B., Mete E. (2016). Exogenous salicylic acid alleviates cold damage by regulating the antioxidative system in two barley (*Hordeum vulgare* L.) cultivars. Front. Life Sci..

[87] Sun L., Li X., Wang Z., Sun Z., Zhu X., Liu S., Song F., Liu F., Wang Y. (2018). Cold priming induced tolerance to subsequent low temperature stress is enhanced by melatonin application during recovery in wheat. Molecules.

[88] Li X., Yan D., Jiang D., Liu F. (2016). Melatonin enhances cold tolerance in drought-primed wild-type and abscisic acid-deficient mutant barley. J. Pineal Res..

[89] Marta B., Szafrańska K., Posmyk M.M. (2016). Exogenous melatonin improves antioxidant defense in cucumber seeds (*Cucumis sativus* L.) germinated under chilling stress. Front. Plant Sci..

[90] Park S., Back K. (2012). Melatonin promotes seminal root elongation and root growth in transgenic rice after germination. J. Pineal Res..

[91] Murch S.J., Campbell S.S., Saxena P.K. (2001). Role of serotonin and melatonin in plant morphogenesis: Regulation of auxin-induced root organogenesis in in vitro cultured explants of *Hypericum perforatum* L.. Vitr. Cell. Dev. Biol. Plant.

[92] Fan J., Hu Z., Xie Y., Chan Z., Chen K., Amombo E., Chen L., Fu J. (2015). Alleviation of cold damage to photosystem II and metabolism by melatonin in bermudagrass. Front. Plant Sci..

[93] Turk H., Erdal S., Genisel M., Atici O., Demir Y., Yanmis D. (2014). The regulatory effect of melatonin on physiological, biochemical and molecular parameters in cold-stressed wheat seedlings. Plant Growth Regul..

[94] Zhang H., Liu L., Wang Z., Feng G., Gao Q., Li X. (2021). Induction of low temperature tolerance in wheat by pre-soaking and parental treatment with melatonin. Molecules.

[95] Cao Q., Li G., Cui Z., Yang F., Jiang X., Diallo L., Kong F. (2019). Seed priming with melatonin improves germination of waxy maize under chilling stress via antioxidant system and starch metabolism. Sci. Rep..

[96] Hernández I.G., Gomez F.J., Cerutti S., Arana M.V., Silva M.F. (2015). Melatonin in Arabidopsis thaliana acts as plant growth regulator at low concentrations and preserves seed viability at high concentrations. Plant Physiol. Biochem..

[97] Simlat M., Szewczyk A., Ptak A. (2020). Melatonin promotes seed germination under salinity and enhances biosynthesis of steviol glycosides in Stevia rebaudiana Bertoni leaves. PLoS ONE.

[98] Tasgin E., Atici O., Nalbantoglu B. (2003). Effects of salicylic acid and cold on freezing tolerance in winter wheat leaves. Plant Growth Regul..

[99] Horvath E., Szalai G., Janda T. (2007). Induction of abiotic stress tolerance by salicylic acid signaling. J. Plant Growth Regul..

[100] Wang W., Wang X., Zhang J., Huang M., Cai J., Zhou Q., Dai T., Jiang D. (2020). Salicylic acid and cold priming induce late-spring freezing tolerance by maintaining cellular redox homeostasis and protecting photosynthetic apparatus in wheat. Plant Growth Regul..

[101] Chen S., Zime L., Cui J., Jiang D., Xia X., Liu D., Yu J. (2011). Alleviation of chilling-induced oxidative damage by salicylic acid pretreatment and related gene expression in eggplant seedlings. Plant Growth Regul..

[102] Ding C.K., Wang C., Gross K.C., Smith D.L. (2002). Jasmonate and salicylate induce expression of pathogenesis-related protein genes and increase resistance to chilling injury in tomato fruit. Planta.

[103] Wang W., Wang X., Huang M., Cai J., Zhou Q., Dai T., Jiang D. (2020). Alleviation of field low-temperature stress in winter wheat by exogenous application of salicylic acid. J. Plant Growth Regul..

[104] Esim N., Atici Ö., Mutlu S. (2013). Effects of exogenous nitric oxide in wheat seedlings under chilling stress. Toxicol. Ind. Health.

[105] Siddiqui M.H., Al-Whaibi M.H., Basalah M.O. (2011). Role of nitric oxide in tolerance of plants to abiotic stress. Protoplasma.

[106] Sun X.-C., Hu C.-X., Tan Q.-L. (2006). Effects of molybdenum on antioxidative defense system and membrane lipid peroxidation in winter wheat under low-temperature stress. J. Plant Physiol. Mol. Biol..

[107] Al-Issawi M., Rihan H.Z., Al-Shmgani H., Fuller M.P. (2016). Molybdenum application enhances antioxidant enzyme activity and COR15a protein expression under cold stress in wheat. J. Plant Interact..

[108] Chu J., Yao X., Zhang Z. (2010). Responses of wheat seedlings to exogenous selenium supply under cold stress. Biol. Trace Elem. Res..

[109] Etesami H., Jeong B.R. (2018). Silicon (Si): Review and future prospects on the action mechanisms in alleviating biotic and abiotic stresses in plants. Ecotoxicol. Environ. Saf..

[110] Hajiboland R., Ahmad P., Prasad M.N.V. (2012). Effect of micronutrient deficiencies on plant stress responses. Abiotic Stress Responses in Plants.

[111] Liang Y., Zhu J., Li Z., Chu G., Ding Y., Zhang J., Sun W. (2008). Role of silicon in enhancing resistance to freezing stress in two contrasting winter wheat cultivars. Environ. Exp. Bot..

[112] Liu Y.Y., Li J.Z., Chen L., Cui D.Q. (2006). Effects of low temperature stress on peroxidation products of membrane lipids and activities of related enzymes in wheat seedling leaves. J. Triticeae Crops.

[113] Chang T., Zhao Y., He H., Xi Q., Fu J., Zhao Y. (2020). Exogenous melatonin improves growth in hulless barley seedlings under cold stress by influencing the expression rhythms of circadian clock genes. PeerJ.

[114] Chen Q., Qi W.-B., Reiter R.J., Wei W., Wang B.-M. (2009). Exogenously applied melatonin stimulates root growth and raises endogenous indoleacetic acid in roots of etiolated seedlings of Brassica juncea. J. Plant Physiol..

[115] Hayat S., Ahmad A. (2007). Salicylic Acid: A Plant Hormone.

[116] Tasgin E., Atici O., Nalbantoglu B., Popova L.P. (2006). Effects of salicylic acid and cold treatments on protein levels and on the activities of antioxidant enzymes in the apoplast of winter wheat leaves. Phytochemistry.

[117] Liang Y., Nikolic M., Bélanger R., Gong H., Song A. (2015). Silicon-mediated tolerance to drought and low-temperature stress. Silicon in Agriculture.

[118] Joudmand A., Hajiboland R. (2019). Silicon mitigates cold stress in barley plants via modifying the activity of apoplasmic enzymes and concentration of metabolites. Acta Physiol. Plant..

[119] Liang Y.C., Zhang W.H., Chen Q., Liu Y.L., Ding R.X. (2006). Effect of exogenous silicon (Si) on H+-ATPase activity, phospholipids and fluidity of plasma membrane in leaves of salt-stressed barley (*Hordeum vulgare* L.). Environ. Exp. Bot..

[120] Itoh S. (2002). Application of mechanistic model for phosphorus uptake by barley under low temperature conditions. Soil Sci. Plant Nutr..

[121] Wang Z., Li S., Jian S., Ye F., Wang T., Gong L., Li X. (2022). Low temperature tolerance is impaired by polystyrene nanoplastics accumulated in cells of barley (*Hordeum vulgare* L.) plants. J. Hazard. Mater..

[122] Lian J., Wu J., Xiong H., Zeb A., Yang T., Su X., Su L., Liu W. (2019). Impact of polystyrene nanoplastics (PSNPs) on seed germination and seedling growth of wheat (*Triticum aestivum* L.). J. Hazard. Mater..

[123] Borna Ali B., Sadeghzadeh N., Hajiboland R. (2023). The impact of algal extract as a biostimulant on cold stress tolerance in barley (*Hordeum vulgare* L.). J. Appl. Phycol..

[124] Parniske M. (2008). Arbuscular mycorrhiza: The mother of plant root endosymbiosis. Nat. Rev. Microbiol..

[125] Hajiboland R., Ahmad P., Azooz M.M., Prasad M.N.V. (2013). Role of arbuscular mycorrhiza in amelioration of salinity. Salt Stress in Plants.

[126] Latef A.A.A.H., Hashem A., Rasool S., Abd Allah E.F., Alqarawi A.A., Egamberdieva D., Jan S., Anjum N.A., Ahmad P. (2016). Arbuscular mycorrhizal symbiosis and abiotic stress in plants: A review. J. Plant Biol..

[127] Hajiboland R., Joudmand A., Aliasgharzad N., Tolrá R., Poschenrieder C. (2019). Arbuscular mycorrhizal fungi alleviate low-temperature stress and increase freezing resistance as a substitute for acclimation treatment in barley. Crop Pasture Sci..

[128] Chen S., Jin W., Liu A., Zhang S., Liu D., Wang F., Lin X., He C. (2013). Arbuscular mycorrhizal fungi (AMF) increase growth and secondary metabolism in cucumber subjected to low temperature stress. Sci. Hortic..

[129] Liu Z.L., Li Y.J., Hou H.Y., Zhu X.C., Rai V., He X.Y., Tian C.J. (2013). Differences in the arbuscular mycorrhizal fungi-improved rice resistance to low temperature at two N levels: Aspects of N and C metabolism on the plant side. Plant Physiol. Biochem..

[130] Khalvandi M., Siosemardeh A., Roohi E., Keramati S. (2021). Salicylic acid alleviated the effect of drought stress on photosynthetic characteristics and leaf protein pattern in winter wheat. Heliyon.

[131] Garg K.K., Anantha K.H., Jat M.L., Kumar S., Sawargaonkar G., Singh A., Gathala M.K. (2024). Managing Soil Drought.

[132] Abid M., Ali S., Qi L.K., Zahoor R., Tian Z., Jiang D., Snider J.L., Dai T. (2018). Physiological and biochemical changes during drought and recovery periods at tillering and jointing stages in wheat (*Triticum aestivum* L.). Sci. Rep..

[133] Nyaupane S., Poudel M.R., Panthi B., Dhakal A., Paudel H., Bhandari R. (2024). Drought stress effect, tolerance, and management in wheat—A review. Cogent Food Agric..

[134] Tiwari J.K., Munshi A.D., Kumar R., Pandey R.N., Arora A., Bhat J.S., Sureja A.K. (2010). Effect of salt stress on cucumber: Na^+^/K^+^ ratio, osmolyte concentration, phenols and chlorophyll content. Acta Physiol. Plant..

[135] Rahman A., Mostofa M.G., Nahar K., Hasanuzzaman M., Fujita M. (2016). Exogenous calcium alleviates cadmium-induced oxidative stress in rice (*Oryza sativa* L.) seedlings by regulating the antioxidant defense and glyoxalase systems. Braz. J. Bot..

[136] Kebede H., Liu X., Jin J., Xing F. (2020). Current status of major mycotoxins contamination in food and feed in Africa. Food Control.

[137] Samarah N.H., Alqudah A.M., Amayreh J.A., McAndrews G.M. (2009). The effect of late-terminal drought stress on yield components of four barley cultivars. J. Agron. Crop Sci..

[138] Sharma A., Zheng B. (2019). Melatonin mediated regulation of drought stress: Physiological and molecular aspects. Plants.

[139] Fatima S., Fatima M., Fatima T., Sarfraz A., Qureshi H., Anwar T., El-Beltagi H.S., Ismoilov I., Tukhtaboeva F., Rebouh N.Y. (2025). Enhancing drought tolerance in wheat using zinc and iron nanoparticles: Implications for sustainable industrial crop productivity. Ind. Crops Prod..

[140] El-Samad H.M.A., Shaddad M.A.K., Ragaey M.M. (2019). Drought strategy tolerance of four barley cultivars and combined effect with salicylic acid application. Am. J. Plant Sci..

[141] Islam S.M.N., Paul N., Rahman M.M., Haque M.A., Rohman M.M., Mostofa M.G. (2023). Salicylic acid application mitigates oxidative damage and improves the growth performance of barley under drought stress. Phyton.

[142] Shoaa M., Mohajeri F., Owji M.R., Bagheri A. (2024). Effects of salicylic acid on quantitative yield and some biochemical traits of three barley varieties under different irrigation regimes. J. Crop Sci. Res. Arid. Reg..

[143] Sorahinobar M., Yusefieh N., Rezayian M., Shahbazi S. (2025). Multifaceted role of *Trichoderma harzianum* isolates in mitigating drought stress and promoting adaptive responses in barley cultivars. Sci. Rep..

[144] Shaw K., Thorne S., Chapman C., Fleming A., Hartley S., Gray J. (2025). The beneficial impact of silicon on wheat drought resilience is dependent on cultivar and stress intensity. Front. Plant Sci..

[145] Ashfaq W., Kaleem M., Brodie G., Fuentes S., Pang A., Gupta D. (2025). Silicon alleviates drought stress by up-regulating physiological and biochemical responses in two contrasting bread wheat cultivars. Cereal Res. Commun..

[146] Cui G., Zhao X., Liu S., Sun F., Zhang C., Xi Y. (2017). Beneficial effects of melatonin in overcoming drought stress in wheat seedlings. Plant Physiol. Biochem..

[147] Li D., Batchelor W.D., Zhang D., Miao H., Li H., Song S., Li R. (2020). Analysis of melatonin regulation of germination and antioxidant metabolism in different wheat cultivars under polyethylene glycol stress. PLoS ONE.

[148] Overmyer K., Brosché M., Kangasjärvi J. (2003). Reactive oxygen species and hormonal control of cell death. Trends Plant Sci..

[149] Siddiqui M.H., Alamri S., Alsubaie Q.D., Ali H.M. (2020). Melatonin and gibberellic acid promote growth and chlorophyll biosynthesis by regulating antioxidant and methylglyoxal detoxification systems in tomato seedlings under salinity. J. Plant Growth Regul..

[150] Castro-Camba R., Sánchez C., Vidal N., Vielba J.M. (2022). Plant development and crop yield: The role of gibberellins. Plants.

[151] Portilho-Carneiro F.A., Padilha F.A., Gomes-Júnior C.C. (2014). ABA application to maize hybrids contrasting for drought tolerance: Changes in water parameters and antioxidant enzyme activity. Plant Growth Regul..

[152] Luo M., Wang D., Delaplace P., Pan Y., Zhou Y., Tang W., Chen K., Chen J., Xu Z., Ma Y. (2023). Melatonin enhances drought tolerance by affecting jasmonic acid and lignin biosynthesis in wheat (*Triticum aestivum* L.). Plant Physiol. Biochem..

[153] Liu J.L., Wang W.X., Wang L.Y., Sun Y. (2015). Exogenous melatonin improves seedling health index and drought tolerance in tomato. Plant Growth Regul..

[154] Wang P., Sun X., Chang C., Feng F., Liang D., Cheng L., Ma F. (2013). Delay in leaf senescence of Malus hupehensis by long-term melatonin application is associated with its regulation of metabolic status and protein degradation. J. Pineal Res..

[155] Li X., Liu J., Zhang C., Liu Z., Guo X., Li S., Li H., Liu K., Li K., Ding M. (2025). Melatonin promotes yield increase in wheat by regulating its antioxidant system and growth under drought stress. Biology.

[156] Zhang Z., Guo L., Sun H., Wu J., Liu L., Wang J., Wang B., Wang Q., Sun Z., Li D. (2023). Melatonin increases drought resistance through regulating the fine root and root hair morphology of wheat revealed with RhizoPot. Agronomy.

[157] Ilyas N., Gull R., Mazhar R., Saeed M., Kanwal S., Shabir S., Bibi F. (2017). Influence of salicylic acid and jasmonic acid on wheat under drought stress. Commun. Soil Sci. Plant Anal..

[158] Kang G., Li G., Xu W., Peng X., Han Q., Zhu Y., Guo T. (2012). Proteomics reveals the effects of salicylic acid on growth and tolerance to subsequent drought stress in wheat. J. Proteome Res..

[159] Akin S., Kaya C. (2024). Impact of salicylic acid and sodium hydrosulfide applied singly or in combination on drought tolerance and grain yield in wheat plants. Food Energy Secur..

[160] Zhang J., Corpas F.J., Li J., Xie Y. (2022). Hydrogen sulfide and reactive oxygen species in plant antioxidant defense and abiotic stress tolerance mechanisms. Int. J. Mol. Sci..

[161] Ali Q., Ahmad M., Kamran M., Ashraf S., Shabaan M., Babar B.H., Zulfiqar U., Haider F.U., Ali M.A., Elshikh M.S. (2023). Synergistic effects of rhizobacteria and salicylic acid on maize salt-stress tolerance. Plants.

[162] Lamlom S.F., Abdelghany A.M., Farouk A.S., Alwakel E.S., Makled K.M., Bukhari N.A., Hatamleh A.A., Ren H., El-Sorady G.A., Shehab A.A. (2025). Biochemical and yield response of spring wheat to drought stress through gibberellic and abscisic acids. BMC Plant Biol..

[163] Hedrich R., Shabala S. (2018). Stomata in a saline world. Curr. Opin. Plant Biol..

[164] Sedaghat M., Emam Y., Mokhtassi-Bidgoli A., Hazrati S., Lovisolo C., Visentin I., Cardinale F., Tahmasebi-Sarvestani Z. (2021). The potential of the synthetic strigolactone analogue GR24 for the maintenance of photosynthesis and yield in winter wheat under drought: Investigations on the mechanisms of action and delivery modes. Plants.

[165] Liu J., He H., Vitali M., Visentin I., Charnikhova T., Haider I., Schubert A., Ruyter-Spira C., Bouwmeester H.J., Lovisolo C. (2015). Osmotic stress represses strigolactone biosynthesis in *Lotus japonicus* roots: Exploring the interaction between strigolactones and ABA under abiotic stress. Planta.

[166] Abdelaal K.A.A., Attia K.A., Alamery S.F., El-Afry M.M., Ghazy A.I., Tantawy D.S., Al-Doss A.A., El-Shawy E.E., Abu-Elsaoud A.M., Hafez Y.M. (2020). Exogenous application of proline and salicylic acid can mitigate the injurious impacts of drought stress on barley plants associated with physiological and histological characters. Sustainability.

[167] Yadavi A., Aboueshaghi R.S., Dehnavi M.M., Balouchi H. (2014). Effect of micronutrient foliar application on grain qualitative characteristics and physiological traits of bean (*Phaseolus vulgaris* L.) under drought stress. Indian J. Fundam. Appl. Life Sci..

[168] Nusrat P., Ashraf M. (2010). Role of silicon in mitigating the adverse effects of salt stress on growth and photosynthetic attributes of two maize (*Zea mays* L.) cultivars grown hydroponically. Pak. J. Bot..

[169] Zhang C., Moutinho-Pereira J.M., Correia C., Coutinho J., Gonçalves A., Guedes A., Gomes-Laranjo J. (2013). Foliar application of Sili-K^®^ increases chestnut (*Castanea* spp.) growth and photosynthesis, simultaneously increasing susceptibility to water deficit. Plant Soil.

[170] Yavas I., Unay A. (2016). Effects of zinc and salicylic acid on wheat under drought stress. J. Anim. Plant Sci..

[171] Aldesuquy H.S., Abbas M.A., Abo-Hamed S.A., Elhakem A.H., Alsokari S.S. (2012). Glycine betaine and salicylic acid induced modification in productivity of two wheat cultivars grown under water stress. J. Stress Physiol. Biochem..

[172] Azimi M.S., Daneshian J., Sayfazadeh S., Zare S. (2013). Evaluation of amino acid and salicylic acid application on yield and growth of wheat under water deficit. Int. J. Agric. Crop Sci..

[173] Ding M., Xiao C., Li S., Liu J., Kong Y., Zhang C., Li H., Yang Y., Liu K., Rehman M. (2025). Effects of iron, zinc, and silicon nanoparticles on morphophysiological growth, yield, and quality of wheat (*Triticum aestivum* L.) under drought stress. J. Hazard. Mater. Adv..

[174] Wang X., Xu H., Wang P., Yang H. (2023). Nanoparticles in plants: Uptake, transport and physiological activity in leaf and root. Materials.

[175] Yadav A., Babu S., Krishnan P., Kaur B., Bana R.S., Chakraborty D., Kumar V., Joshi B., Lal S.K. (2024). Zinc oxide and ferric oxide nanoparticle combination increases plant growth, yield, and quality of soybean under semiarid regions. Chemosphere.

[176] Alhammad B.A., Abdel-Aziz H.M.M., Seleiman M.F., Tourky S.M.N. (2023). How can biological and chemical silver nanoparticles positively impact physio-chemical and chloroplast ultrastructural characteristics of Vicia faba seedlings?. Plants.

[177] Qureshi A.K., Farooq U., Shakeel Q., Ali S., Ashiq S., Shahzad S., Tariq M., Seleiman M.F., Jamal A., Saeed M.F. (2023). Green synthesis of silver nanoparticles from *Avena fatua* extract: Antifungal activity against *Fusarium oxysporum* f. sp. *lycopersici*. Pathogens.

[178] El-Saadony M.T., Saad A.M., Soliman S.M., Salem H.M., Desoky E.M., Babalghith A., El-Tahan A.M., Ibrahim O.M., Ebrahim A.A.M., Abd El-Mageed T.A. (2022). Role of nanoparticles in enhancing crop tolerance to abiotic stress: A comprehensive review. Front. Plant Sci..

[179] Raza M.A.S., Muhammad F., Farooq M., Aslam M.U., Akhter N., Toleikienė M., Binobead M.A., Ali M.A., Rizwan M., Iqbal R. (2025). ZnO nanoparticles and stage-based drought tolerance in wheat (*Triticum aestivum* L.): Effects on morphophysiology, nutrient uptake, grain yield, and quality. Sci. Rep..

[180] Srivastav A., Ganjewala D., Singhal R.K., Rajput V.D., Minkina T., Voloshina M., Srivastava S., Shrivastava M. (2021). Effect of ZnO nanoparticles on growth and biochemical responses of wheat and maize. Plants.

[181] Xu L., Islam F., Ali B., Pei Z., Li J., Ghani M.A., Zhou W. (2017). Silicon and water-deficit stress differentially modulate physiology and ultrastructure in wheat (*Triticum aestivum* L.). 3 Biotech.

[182] Raza M.A.S., Zulfiqar B., Iqbal R., Muzamil M.N., Aslam M.U., Muhammad F., Amin J., Aslam H.M.U., Ibrahim M.A., Uzair M. (2023). Morpho-physiological and biochemical response of wheat to various treatments of silicon nanoparticles under drought stress conditions. Sci. Rep..

[183] Ashfaq W., Fuentes S., Brodie G., Gupta D. (2022). The role of silicon in regulating physiological and biochemical mechanisms of contrasting bread wheat cultivars under terminal drought and heat stress environments. Front. Plant Sci..

[184] Talaat N.B. (2023). Drought stress alleviator melatonin reconfigures water-stressed barley (*Hordeum vulgare* L.) plants’ photosynthetic efficiency, antioxidant capacity and endogenous phytohormone profile. Int. J. Mol. Sci..

[185] Chen Z., Jia W., Li S., Xu J., Xu Z. (2021). Enhancement of Nicotiana tabacum resistance against dehydration-induced leaf senescence via metabolite/phytohormone-gene regulatory networks modulated by melatonin. Front. Plant Sci..

[186] Tan X.-L., Fan Z.-Q., Kuang J.-F., Lu W.-J., Reiter R.J., Lakshmanan P., Su X.-G., Zhou J., Chen J.-Y., Shan W. (2019). Melatonin delays leaf senescence of Chinese flowering cabbage by suppressing ABFs-mediated abscisic acid biosynthesis and chlorophyll degradation. J. Pineal Res..

[187] Altaf M.A., Shahid R., Ren M.-X., Altaf M.M., Khan L.U., Shahid S., Jahan M.S. (2021). Melatonin alleviates salt damage in tomato seedlings: Root architecture, photosynthetic capacity, ion homeostasis and antioxidant enzyme analysis. Sci. Hortic..

[188] Dai L., Li J., Harmens H., Zheng X., Zhang C. (2020). Melatonin enhances drought resistance by regulating leaf stomatal behaviour, root growth, and catalase activity in two contrasting rapeseed (*Brassica napus* L.) genotypes. Plant Physiol. Biochem..

[189] Ye F., Jiang M., Zhang P., Liu L., Liu S., Zhao C., Li X. (2022). Exogenous melatonin reprograms the rhizosphere microbial community to modulate the responses of barley to drought stress. Int. J. Mol. Sci..

[190] Habibi N. (2012). Exogenous salicylic acid alleviates oxidative damage of barley plants under drought stress. Acta Biol. Szeged..

[191] Rady M.M., Boriek S.H.K., El-Mageed T.A.A., El-Yazal M.A.S., Ali E.F., Hassan F.A.S., Abdelkhalik A. (2021). Exogenous gibberellic acid or dilute bee honey boosts drought stress tolerance in Vicia faba by rebalancing osmoprotectants, antioxidants, nutrients and phytohormones. Plants.

[192] Askarnejad M.R., Soleymani A., Javanmard H.R. (2021). Barley (*Hordeum vulgare* L.) physiology, including nutrient uptake, affected by plant growth regulators under field drought conditions. J. Plant Nutr..

[193] Collin A., Matkowski H., Sybilska E., Biantari A., Król O., Daszkowska-Golec A. (2025). ABA-induced alternative splicing drives transcriptomic reprogramming for drought tolerance in barley. BMC Plant Biol..

[194] Hasan M.M., Gong L., Nie Z.F., Li F.P., Ahammed G.J., Fang X.W. (2021). ABA-induced stomatal movements in vascular plants during dehydration and rehydration. Environ. Exp. Bot..

[195] Skowron E., Trojak M. (2021). Effect of exogenously applied abscisic acid, putrescine and hydrogen peroxide on drought tolerance of barley. Biologia.

[196] Vasanthi N., Saleena L.M., Raj S.A. (2014). Silicon in crop production and crop protection—A review. Agric. Rev..

[197] Bijanzadeh E., Naderi R., Barati V. (2018). Influence of silicon priming on seedling growth, root xylem anatomy and ion accumulation of barley (*Hordeum vulgare* L.) under drought stress. J. Plant Process Funct..

[198] Mahmoud A.W.M., Mottaleb S.A., Rowezak M.M., Salama A.M. (2021). The role of nano-silicon and other soil conditioners in improving physiology and yield of drought-stressed barley crop. Agriculture (Pol’nohospodárstvo).

[199] Mahmoud H., Youssif S. (2015). Response of garlic (*Allium sativum* L.) to natural fertilizers and ores under Ras Sudr conditions. Middle East J. Appl. Sci..

[200] Zafar F., Noreen Z., Shah A.A., Usman S. (2023). Co-application of humic acid, potassium dihydrogen phosphate and melatonin ameliorates the effects of drought stress in barley (*Hordeum vulgare* L.). J. Soil Sci. Plant Nutr..

[201] Abdelaal K., Hafez Y.M., El-Afry M.M., Tantawy D.S., Alshaal T. (2018). Effect of some osmoregulators on photosynthesis, lipid peroxidation, antioxidative capacity, and productivity of barley (*Hordeum vulgare* L.) under water deficit stress. Environ. Sci. Pollut. Res..

[202] Shen J., Guo M.J., Wang Y.G., Yuan X.Y., Wen Y.Y., Song X.E., Dong S.Q., Guo P.Y. (2020). Humic acid improves the physiological and photosynthetic characteristics of millet seedlings under drought stress. Plant Signal. Behav..

[203] Fallahi H.R., Ghorbany M., Aghhavani-Shajari M., Samadzadeh A., Asadian A.H. (2017). Qualitative response of roselle to planting methods, humic acid application, mycorrhizal inoculation and irrigation management. J. Crop Improv..

[204] Harman G.E., Doni F., Khadka R.B., Uphoff N. (2019). Endophytic strains of *Trichoderma* increase plants’ photosynthetic capability. J. Appl. Microbiol..

[205] Illescas M., Morán-Diez M.E., Martínez de Alba Á.E., Hermosa R., Monte E. (2022). Effect of *Trichoderma asperellum* on wheat plants’ biochemical and molecular responses and yield under different water stress conditions. Int. J. Mol. Sci..

[206] Zhang S., Xu B., Gan Y. (2019). Seed treatment with *Trichoderma longibrachiatum* T6 promotes wheat seedling growth under NaCl stress through activating enzymatic and nonenzymatic antioxidant defense systems. Int. J. Mol. Sci..

[207] Chakraborti S., Bera K., Sadhukhan S., Dutta P. (2022). Bio-priming of seeds: Plant stress management and its underlying cellular, biochemical and molecular mechanisms. Plant Stress.

[208] Tyśkiewicz R., Nowak A., Ozimek E., Jaroszuk-Ściseł J. (2022). *Trichoderma*: The current status of its application in agriculture for the biocontrol of fungal phytopathogens and stimulation of plant growth. Int. J. Mol. Sci..

[209] Shukla N., Awasthi R.P., Rawat L., Kumar J. (2012). Biochemical and physiological responses of rice (*Oryza sativa* L.) as influenced by Trichoderma harzianum under drought stress. Plant Physiol. Biochem..

[210] Afifi H.H., Hassan N.A., Abou-Hadid A.F., Madkor M.A. (2021). The ability of selected *Trichoderma* spp. to improve wheat (*Triticum aestivum* L.) tolerance under heat, salinity and drought stresses through seed treatments. Plant Cell Biotechnol. Mol. Biol..

[211] Gaikwad N., Verma S. (2024). Effect of *Trichoderma harzianum* on growth of corn under water stress condition. Int. J. Plant Soil Sci..

[212] Tiwari M., Devi B., Sinha S., Yadav N., Singh P. (2024). Intergenerational priming by Trichoderma alleviates drought stress in barley. Environ. Exp. Bot..

[213] Creus C.M., Pereyra M.A., Casanovas E.M., Sueldo R.J., Barassi C.A. (2010). Plant growth-promoting effects of rhizobacteria on abiotic stressed plants: Azospirillum–grasses model. Am. J. Plant Sci. Biotechnol..

[214] Pereyra M.A., Zalazar C.A., Barassi C.A. (2006). Root phospholipids in *Azospirillum* inoculated wheat seedlings exposed to water stress. Plant Physiol. Biochem..

[215] Bonab B.F., Roveshti A.A., Abbasi A. (2022). Changes in antioxidant enzymes of barley (*Hordeum vulgare* L.) using the endophytic fungus Piriformospora indica and the bacterium Azospirillum spp. under drought stress. Poljopr. Sumar..

[216] Amina A.A., Hanan H.L.L. (2011). Differential effects of paclobutrazol on water stress alleviation through electrolyte leakage, phytohormones, reduced glutathione and lipid peroxidation in some wheatgenotypes (*Triticum aestivum* L.) grown in vitro. Rom. Biotechnol. Lett..

[217] Vurukonda S.S.K.P., Vardharajula S., Shrivastava M., SkZ A. (2016). Enhancement of drought stress tolerance in crops by plant growth-promoting rhizobacteria. Microbiol. Res..

[218] El Sabagh A., Islam M.S., Skalicky M., Ali Raza M., Singh K., Anwar Hossain M., Hossain A., Mahboob W., Iqbal M.A., Ratnasekera D. (2021). Salinity stress in wheat (*Triticum aestivum* L.) in the changing climate: Adaptation and management strategies. Front. Agron..

[219] FAO (2024). FAO Launches First Major Global Assessment of Salt-Affected Soils in 50 Years.

[220] Thorne S.J., Hartley S.E., Maathuis F.J.M. (2020). Is silicon a panacea for alleviating drought and salt stress in crops?. Front. Plant Sci..

[221] Farooq M.A., Saqib Z.A., Akhtar J., Bakhat H.F., Pasala R.K., Dietz K.J. (2019). Protective role of silicon against combined stress of salinity and boron toxicity by improving antioxidant enzyme activity in rice. Silicon.

[222] Shah Z.H., Rehman H.M., Akhtar T., Daur I., Nawaz M.A., Ahmad M.Q., Rana I.A., Atif R.M., Yang S.H., Chung G. (2017). Redox and ionic homeostasis regulations against oxidative, salinity and drought stress in wheat: A systems biology approach. Front. Genet..

[223] Naveed M., Sajid H., Mustafa A., Niamat B., Ahmad Z., Yaseen M., Kamran M., Rafique M., Ahmar S., Chen J.T. (2020). Alleviation of salinity-induced oxidative stress, improvement in growth, physiology, and mineral nutrition of canola (*Brassica napus* L.) through calcium-fortified composted animal manure. Sustainability.

[224] Qureshi A. (2016). Perspectives for bio-management of salt-affected and waterlogged soils in Pakistan. Agroforestry for the Management of Waterlogged Saline Soils and Poor-Quality Waters.

[225] Torabian S., Zahedi M., Khoshgoftar A.H. (2016). Effects of foliar spray of two kinds of zinc oxide on the growth and ion concentration of sunflower cultivars under salt stress. J. Plant Nutr..

[226] Abdelrady W.A., Ma Z., Elshawy E.E., Wang L., Askri S.M.H., Ibrahim Z., Denni E., Kanwal F., Zeng F., Shamsi I.H. (2024). Physiological and biochemical mechanisms of salt tolerance in barley under salinity stress. Plant Stress.

[227] Shamloo-Dashtpagerdi R., Lindlöf A., Niazi A., Pirasteh-Anosheh H. (2019). LOS2 gene plays a potential role in barley (*Hordeum vulgare* L.) salinity tolerance as a hub gene. Mol. Breed..

[228] El-Tayeb M.A. (2005). Response of barley grains to the interactive effect of salinity and salicylic acid. Plant Growth Regul..

[229] Seckin B., Türkan I., Sekmen A.H., Ozfidan C. (2010). The role of antioxidant defense system in differential salt tolerance of *Hordeum marinum* Huds. and *Hordeum vulgare* L.. Environ. Exp. Bot..

[230] Ghafoor M.F., Ali Q., Malik A. (2020). Effects of salicylic acid priming for salt stress tolerance in wheat. Biol. Clin. Sci. Res. J..

[231] Shah S.M.O., Jamal Y., Haq I.U. (2024). Effect of humic acid on wheat (*Triticum aestivum* L.) under saline conditions. Pure Appl. Biol..

[232] Alghabari F., Shah Z.H. (2025). Deciphering salt tolerance mechanisms in synthetic hexaploid and bread wheat under humic acid application: Physiological and genetic perspectives. Front. Plant Sci..

[233] Torun H., Novák O., Mikulík J., Pěnčík A., Strnad M., Ayaz F.A. (2020). Timing-dependent effects of salicylic acid treatment on phytohormonal changes, ROS regulation, and antioxidant defense in salinized barley (*Hordeum vulgare* L.). Sci. Rep..

[234] Munns R., Tester M. (2008). Mechanisms of salinity tolerance. Annu. Rev. Plant Biol..

[235] Pirasteh-Anosheh H., Ranjbar G., Hasanuzzaman M., Khanna K., Bhardwaj R., Ahmad P. (2022). Salicylic acid-mediated regulation of morpho-physiological and yield attributes of wheat and barley plants in deferring salinity stress. J. Plant Growth Regul..

[236] Farzana S., Rasel M., Tahjib Ul Arif M., Hossain M.A., Azam M.G., Al Galib M.A., Mahamud A.S.U., Hossain M.A. (2021). Salicylic acid and thiourea ameliorate the negative impact of salt stress in wheat seedlings by up-regulating photosynthetic pigments, leaf water status, and antioxidant defense system. J. Phytol..

[237] Al-Rawi O.H., Abdukafoor A.H., Yousif S.I., Al-Shaheen M. (2018). Effect of spraying with different levels of salicylic and humic acid on some growth characteristics and yield of wheat (*Triticum aestivum* L.). Eurasia Proc. Sci. Technol. Eng. Math..

[238] Matysiak K., Siatkowski I., Kierzek R., Kowalska J., Krawczyk R. (2020). Effect of foliar-applied acetylsalicylic acid on wheat (*Triticum aestivum* L.) under field conditions. Agronomy.

[239] Dolatabadian A., Modarres Sanavy S.A.M., Sharifi M. (2009). Effect of salicylic acid and salt on wheat seed germination. Acta Agric. Scand. Sect. B Soil Plant Sci..

[240] Kaydan D., Yağmur M., Okut N. (2007). Effects of salicylic acid on the growth and some physiological characters in salt stressed wheat (*Triticum aestivum* L.). Tarim Bilim. Derg..

[241] Erdal S., Aydın M., Genisel M., Taşpınar M.S., Dumlupinar R., Kaya O., Gorcek Z. (2011). Effects of salicylic acid on wheat salt sensitivity. Afr. J. Biotechnol..

[242] Fairoj S.A., Islam M.M., Islam M.A., Zaman E., Momtaz M.B., Hossain M.S., Jahan N.A., Shams S., Urmi T.A., Rasel M.A. (2023). Salicylic acid improves agro-morphology, yield and ion accumulation of two wheat (*Triticum aestivum* L.) genotypes by ameliorating the impact of salt stress. Agronomy.

[243] Morad M., Sara S., Mohammad D., Ramin J.M., Majid R. (2013). Effect of salicylic acid on alleviation of salt stress on growth and some physiological traits of wheat. Int. J. Biosci..

[244] Alsahli A., Mohamed A.K., Alaraidh I., Al-Ghamdi A., Al-Watban A., El-Zaidy M., Alzahrani S.M. (2019). Salicylic acid alleviates salinity stress through the modulation of biochemical attributes and some key antioxidants in wheat seedlings. Pak. J. Bot..

[245] Wang J., Lv P., Yan D., Zhang Z., Xu X., Wang T., Wang Y., Peng Z., Yu C., Gao Y. (2022). Exogenous melatonin improves seed germination of wheat (*Triticum aestivum* L.) under salt stress. Int. J. Mol. Sci..

[246] Yan D., Wang J., Lu Z., Liu R., Hong Y., Su B., Wang Y., Peng Z., Yu C., Gao Y. (2023). Melatonin-mediated enhancement of photosynthetic capacity and photoprotection improves salt tolerance in wheat. Plants.

[247] Chen L., Lu B., Liu L., Duan W., Jiang D., Li J., Zhang K., Sun H., Zhang Y., Li C. (2021). Melatonin promotes seed germination under salt stress by regulating ABA and GA3 in cotton (*Gossypium hirsutum* L.). Plant Physiol. Biochem..

[248] Ahmad I., Munsif F., Mihoub A., Jamal A., Saeed M.F., Babar S., Fawad M., Zia A. (2022). Beneficial effect of melatonin on growth and chlorophyll content in wheat (*Triticum aestivum* L.) grown under salt stress conditions. Gesunde Pflanz..

[249] Talaat N.B., Todorova D. (2022). Antioxidant machinery and glyoxalase system regulation confers salt stress tolerance to wheat (*Triticum aestivum* L.) plants treated with melatonin and salicylic acid. J. Soil Sci. Plant Nutr..

[250] Talaat N.B., Shawky B.T. (2022). Synergistic effects of salicylic acid and melatonin on modulating ion homeostasis in salt-stressed wheat (*Triticum aestivum* L.) plants by enhancing root H^+^-pump activity. Plants.

[251] Bukhat S., Manzoor H., Athar H.U.R., Zafar Z.U., Azeem F., Rasul S. (2020). Salicylic acid-induced photosynthetic adaptability of Raphanus sativus to salt stress is associated with antioxidant capacity. J. Plant Growth Regul..

[252] Hernández-Ruiz J., Arnao M.B. (2018). Relationship of melatonin and salicylic acid in biotic and abiotic plant stress responses. Agronomy.

[253] Park H.S., Kazerooni E.A., Kang S.M., Al-Sadi A.M., Lee I.J. (2021). Melatonin enhances tolerance and recovery mechanisms in Brassica juncea under saline conditions. Front. Plant Sci..

[254] El-Hawary M.M., Hashem O.S.M., Hasanuzzaman M. (2023). Seed priming and foliar application with ascorbic acid and salicylic acid mitigate salt stress in wheat. Agronomy.

[255] Rehman H.U., Alharby H.F., Bamagoos A.A., Abdelhamid M.T., Rady M.M. (2021). Sequenced application of glutathione as an antioxidant with an organic biostimulant improves physiological and metabolic adaptation to salinity in wheat. Plant Physiol. Biochem..

[256] Hu M., Shi Z., Zhang Z., Zhang Y., Li H. (2012). Effects of exogenous glucose on seed germination and antioxidant capacity in wheat seedlings under salt stress. Plant Growth Regul..

[257] Ditta A. (2012). How helpful is nanotechnology in agriculture?. Adv. Nat. Sci. Nanosci. Nanotechnol..

[258] Satti S.H., Raja N.I., Javed B., Akram A., Mashwani Z.U.R., Ahmad M.S., Ikram M. (2021). Titanium dioxide nanoparticles elicited agro-morphological and physicochemical modifications in wheat plants to control Bipolaris sorokiniana. PLoS ONE.

[259] Ahmad P., Sarwat M., Bhat N.A., Wani M.R., Kazi A.G., Tran L.S. (2015). Alleviation of cadmium toxicity in *Brassica juncea* L. (Czern. & Coss.) by calcium application involves various physiological and biochemical strategies. PLoS ONE.

[260] Jiang C., Zu C., Lu D., Zheng Q., Shen J., Wang H., Li D. (2017). Effect of exogenous selenium supply on photosynthesis, Na^+^ accumulation and antioxidative capacity of maize (*Zea mays* L.) under salinity stress. Sci. Rep..

[261] Terry N., Zayed A.M., De Souza M.P., Tarun A.A.S. (2000). Selenium in higher plants. Annu. Rev. Plant Physiol. Plant Mol. Biol..

[262] Hermosillo-Cereceres M.A., Sanchez-Chavez E., Guevara-Aguilar A., Munoz-Marquez E. (2013). Biofortification and distribution patterns of selenium in bean: Response to selenate and selenite. J. Food Agric. Environ..

[263] Elkelish A., Soliman M.H., Alhaithloul H.A., El-Esawi M.A. (2019). Selenium protects wheat seedlings against salt stress-mediated oxidative damage by up-regulating antioxidants and osmolyte metabolism. Plant Physiol. Biochem..

[264] Mustafa N., Raja N.I., Ilyas N., Ikram M., Mashwani Z.U.R., Ehsan M. (2021). Foliar applications of plant-based titanium dioxide nanoparticles to improve agronomic and physiological attributes of wheat (*Triticum aestivum* L.) plants under salinity stress. Green Process. Synth..

[265] Mustafa N., Raja N.I., Ilyas N., Abasi F., Ahmad M.S., Ehsan M., Mehak A., Badshah I., Proćków J. (2022). Exogenous application of green titanium dioxide nanoparticles (TiO_2_ NPs) to improve the germination, physiochemical, and yield parameters of wheat plants under salinity stress. Molecules.

[266] Amjad S.F., Mansoora N., Din I.U., Iqbal R., Khalid J., Jatoi G.H., Murtaza G., Yassen S., Naz M., Danish S. (2021). Application of zinc fertilizer and mycorrhizal inoculation on physio-biochemical parameters of wheat grown under water-stressed environment. Sustainability.

[267] Cakmak I., Kutman U.A. (2018). Agronomic biofortification of cereals with zinc: A review. Eur. J. Soil Sci..

[268] Eker S., Heybet E.H., Barut H., Erdem H. (2013). Effects of zinc on growth and sodium, potassium and calcium concentrations of shoot in bread wheat under salt stress. Fresenius Environ. Bull..

[269] Lalarukh I., Zahra N., Al Huqail A.A., Amjad S.F., Al-Dhumri S.A., Ghoneim A.M., Alshahri A.H., Almutari M.M., Alhusayni F.S., Al-Shammari W.B. (2022). Exogenously applied ZnO nanoparticles induced salt tolerance in potentially high yielding modern wheat (*Triticum aestivum* L.) cultivars. Environ. Technol. Innov..

[270] Zafar S., Hasnain Z., Danish S., Battaglia M.L., Fahad S., Ansari M.J., Alharbi S.A. (2024). Modulations of wheat growth by selenium nanoparticles under salinity stress. BMC Plant Biol..

[271] Ghazi A.A., El-Nahrawy S., El-Ramady H., Ling W. (2022). Biosynthesis of nano-selenium and its impact on germination of wheat under salt stress for sustainable production. Sustainability.

[272] Guo G., Liu X., Sun F., Cao J., Huo N., Wuda B., Xin M., Hu Z., Du J., Xia R. (2018). Wheat miR9678 affects seed germination by generating phased siRNAs and modulating abscisic acid/gibberellin signaling. Plant Cell.

[273] Du W., Yang Y., Peng Q., Liang X., Mao H. (2019). Comparison study of zinc nanoparticles and zinc sulphate on wheat growth: From toxicity to zinc biofortification. Chemosphere.

[274] Sundaria N., Singh M., Upreti P., Chauhan R.P., Jaiswal J.P., Kumar A. (2019). Seed priming with iron oxide nanoparticles triggers iron acquisition and biofortification in wheat (*Triticum aestivum* L.) grains. J. Plant Growth Regul..

[275] Mohamed A.K.S.H., Qayyum M.F., Abdel-Hadi A.M., Rehman R.A., Ali S., Rizwan M. (2017). Interactive effect of salinity and silver nanoparticles on photosynthetic and biochemical parameters of wheat. Arch. Agron. Soil Sci..

[276] Ibrahim M.E.H., Zhu X., Zhou G., Ali A.Y.A., Ahmad I., Elsiddig A.M.I., Zhu G., Nimir N.E.A. (2019). Promoting salt tolerance in wheat seedlings by application of nitrogen fertilizer. Pak. J. Bot..

[277] Murtaza G., Azooz M., Murtaza B., Usman Y., Saqib M., Ahmad P. (2013). Nitrogen-use efficiency (NUE) in plants under NaCl stress. Salt Stress in Plants.

[278] Rady M.M., Desoky E.-S.M., Elrys A.S., Boghdady M.S. (2019). Can licorice root extract be used as an effective natural biostimulant for salt-stressed common bean plants?. S. Afr. J. Bot..

[279] Rehman H., Basra S.M.A., Rady M.M., Ghoneim A.M., Wang Q. (2017). Moringa leaf extract improves wheat growth and productivity by delaying senescence and improving source–sink relationships. Int. J. Agric. Biol..

[280] Bajwa A.A., Farooq M., Nawaz A. (2018). Seed priming with sorghum extracts and benzyl aminopurine improves the tolerance against salt stress in wheat (*Triticum aestivum* L.). Physiol. Mol. Biol. Plants.

[281] Bajwa A.A., Farooq M. (2017). Seed priming with sorghum water extract and benzyl aminopurine along with surfactant improves germination metabolism and early seedling growth of wheat. Arch. Agron. Soil Sci..

[282] Latique S., Ben Mrid R., Kabach I., Kchikich A., Sammama H., Yasri A., Nhiri M., El Kaoua M., Douira A., Selmaoui K. (2021). Foliar application of Ulva rigida water extracts improves salinity tolerance in wheat (*Triticum durum* L.). Agronomy.

[283] Latique S., Mohamed Aymen E., Halima C., Chérif H., El Kaoua M. (2017). Alleviation of salt stress in durum wheat (*Triticum durum* L.) seedlings through the application of liquid seaweed extracts of Fucus spiralis. Commun. Soil Sci. Plant Anal..

[284] Ahmad A., Blasco B., Marto V. (2022). Combating salinity through natural plant extract-based biostimulants: A review. Front. Plant Sci..

[285] Meng Q., Yan M., Zhang J., Zhang Q., Zhang X., Yang Z., Luo Y., Wu W. (2023). Humic acids enhance salt stress tolerance associated with pyrroline-5-carboxylate synthetase gene expression and hormonal alteration in perennial ryegrass (*Lolium perenne* L.). Front. Plant Sci..

[286] Ali A.Y.A., Ibrahim M.E.H., Zhou G., Nimir N.E.A., Jiao X., Zhu G., Elsiddig A.M.I., Zhi W., Chen X., Lu H. (2019). Ameliorative effects of jasmonic acid and humic acid on antioxidant enzymes and salt tolerance of forage sorghum under salinity conditions. Agron. J..

[287] Hatami E., Shokouhian A.A., Ghanbari A.R., Naseri L.A. (2018). Alleviating salt stress in almond rootstocks using humic acid. Sci. Hortic..

[288] Shukry W.M., Abu-Ria M.E., Abo-Hamed S.A., Anis G.B., Ibraheem F. (2023). The efficiency of humic acid for improving salinity tolerance in salt-sensitive rice (*Oryza sativa*): Growth responses and physiological mechanisms. Gesunde Pflanz..

[289] Asik B.B., Turan M.A., Çelik H., Katkat A.V. (2009). Effects of humic substances on plant growth and mineral nutrient uptake of wheat (*Triticum durum* cv. Salihli) under conditions of salinity. Asian J. Crop Sci..

[290] Abbas G., Rehman S., Siddiqui M.H., Ali H.M., Farooq M.A., Chen Y. (2022). Potassium and humic acid synergistically increase salt tolerance and nutrient uptake in contrasting wheat genotypes through ionic homeostasis and activation of antioxidant enzymes. Plants.

[291] Smith S.E., Read D.J. (2010). Mycorrhizal Symbiosis.

[292] Giambalvo D., Amato G., Borgia D., Ingraffia R., Librici C., Lo Porto A., Puccio G., Ruisi P., Frenda A.S. (2022). Nitrogen availability drives mycorrhizal effects on wheat growth, nitrogen uptake and recovery under salt stress. Agronomy.

[293] Pakar N., Pirasteh-Anosheh H., Emam Y., Pessarakli M. (2016). Barley growth, yield, antioxidant enzymes, and ion accumulation affected by plant growth regulators under salinity stress. J. Plant Nutr..

[294] Jelali N., Ben Youssef R., Boukari N., Zorrig W., Dhifi W., Abdelly C. (2021). Salicylic acid and H_2_O_2_ seed priming alleviates Fe deficiency through the modulation of growth, root acidification capacity, and photosynthetic performance in Sulla carnosa. Plant Physiol. Biochem..

[295] Rady M.M., Mohamed G.F. (2015). Modulation of salt stress effects on the growth, physio-chemical attributes, and yields of *Phaseolus vulgaris* L. plants by the combined application of salicylic acid and *Moringa oleifera* leaf extract. Sci. Hortic..

[296] Pirasteh-Anosheh H., Emam Y. (2012). Manipulation of morpho-physiological traits in bread and durum wheat by using plant growth regulators under different water regimes. J. Crop Prod. Process..

[297] Ben Youssef R., Jelali N., Acosta Motos J.R., Abdelly C., Albacete A. (2025). Salicylic acid seed priming: A key frontier in conferring salt stress tolerance in barley seed germination and seedling growth. Agronomy.

[298] Torun H., Novák O., Mikulík J., Strnad M., Ayaz F.A. (2022). The effects of exogenous salicylic acid on endogenous phytohormone status in *Hordeum vulgare* L. under salt stress. Plants.

[299] Hanif S., Mahmood A., Javed T., Bibi S., Zia M.A., Asghar S., Naeem Z., Ercisli S., Rahimi M., Ali B. (2024). Exogenous application of salicylic acid ameliorates salinity stress in barley (*Hordeum vulgare* L.). BMC Plant Biol..

[300] Shamloo-Dashtpagerdi R., Pirasteh-Anosheh H., Tahmaseb S. (2025). Evidence for a JA-responsive SNAC1–ASMT1 regulatory module contributing to melatonin-mediated salinity stress tolerance in barley. J. Plant Physiol..

[301] Yin Y., Xu J., He X., Yang Z., Fang W., Ta J. (2022). Role of exogenous melatonin involved in phenolic acid metabolism of germinated hulless barley under NaCl stress. Plant Physiol. Biochem..

[302] Moori S., Eisvand H.R. (2017). Plant growth regulators and ascorbic acid effects on physiological quality of wheat seedlings obtained from deteriorated seeds. Pak. J. Bot..

[303] Agami R.A. (2014). Applications of ascorbic acid or proline increase resistance to salt stress in barley seedlings. Biol. Plant..

[304] Hassan A., Amjad S.F., Saleem M.H., Yasmin H., Imran M., Riaz M., Ali Q., Joyia F.A., Mobeen M., Ahmed S. (2021). Foliar application of ascorbic acid enhances salinity stress tolerance in barley (*Hordeum vulgare* L.) through modulation of morpho-physio-biochemical attributes, ion uptake, osmoprotectants, and stress response gene expression. Saudi J. Biol. Sci..

[305] Noreen S., Sultan M., Akhter M.S., Shah K.H., Ummara U., Manzoor H., Ulfat M., Alyemeni M.N., Ahmad P. (2021). Foliar fertigation of ascorbic acid and zinc improves growth, antioxidant enzyme activity and harvest index in barley (*Hordeum vulgare* L.) grown under salt stress. Plant Physiol. Biochem..

[306] Billah M., Rohman M., Hossain N., Uddin M.S. (2017). Exogenous ascorbic acid improves salinity tolerance in maize (*Zea mays* L.) by increasing antioxidant activity. Afr. J. Agric. Res..

[307] Mohsin A., Ahmad A., Farooq M., Ullah S. (2014). Influence of zinc application through seed treatment and foliar spray on growth, productivity and grain quality of hybrid maize. J. Anim. Plant Sci..

[308] Seleem E.A., Ibrahim H.M.S., Taha Z.K. (2021). Exogenous application of ascorbic acid and putrescine: A natural eco-friendly potential for alleviating NaCl stress in barley (*Hordeum vulgare*). Emir. J. Food Agric..

[309] Gerami M., Mohammadian A., Akbarpour V. (2019). The effect of putrescine and salicylic acid on physiological characteristics and antioxidant activity in *Stevia rebaudiana* under salinity stress. J. Crop Breed..

[310] Ghalati R.E., Shamili M., Homaei A. (2020). Effect of putrescine on biochemical and physiological characteristics of guava (*Psidium guajava* L.) seedlings under salt stress. Sci. Hortic..

[311] Liang Y., Chen Q., Liu Q., Zhang W., Ding R. (2003). Exogenous silicon increases antioxidant enzyme activity and reduces lipid peroxidation in roots of salt-stressed barley (*Hordeum vulgare* L.). J. Plant Physiol..

[312] Akhter M.S., Noreen S., Ummara U., Aqeel M., Saleem N., Ahmed M.M., Mahmood S., Athar H.U.R., Alyemeni M.N., Kaushik P. (2022). Silicon-induced mitigation of NaCl stress in barley (*Hordeum vulgare* L.) associated with enhanced enzymatic and non-enzymatic antioxidant activities. Plants.

[313] Laifa I., Hajji M., Farhat N., Elkhouni A., Smaoui A., M’nif A., Hamzaoui A.H., Savouré A., Abdelly C., Zorrig W. (2020). Beneficial effects of silicon on sea barley (*Hordeum marinum* Huds.) under salt stress. Plant Cell Tissue Organ Cult..

[314] Stadnik B., Tobiasz-Salach R., Mazurek M. (2022). Physiological and epigenetic reaction of barley (*Hordeum vulgare* L.) to the foliar application of silicon under soil salinity conditions. Int. J. Mol. Sci..

[315] Ben Youssef R., Jelali N., Boukari N., Albacete A., Martinez C., Perez Alfocea F., Abdelly C. (2021). The efficiency of different priming agents for improving germination and early seedling growth of local Tunisian barley under salinity stress. Plants.

[316] Al-Busaidi A., Yamamoto T., Inoue M., Eneji A.E., Mori Y., Irshad M. (2008). Effects of zeolite on soil nutrients and growth of barley following irrigation with saline water. J. Plant Nutr..

[317] El-Sharkawy M.S., El-Beshsbeshy T.R., Hassan S.M., Mahmoud E.K., Abdelkader N.I., Al-Shal R.M., Missaoui A.M. (2017). Alleviating salt stress in barley by use of plant growth stimulants and potassium sulfate. J. Agric. Sci..

[318] Khan W., Rayirath U.P., Subramanian S., Jithesh M.N., Rayorath P., Hodges D.M., Critchley A.T., Craigie J.S., Norrie J., Prithiviraj B. (2009). Seaweed extracts as biostimulants of plant growth and development. J. Plant Growth Regul..

[319] Bensidhoum L., Nabti E.H. (2021). Role of Cystoseira mediterranea extracts in the alleviation of salt stress adverse effects and enhancement of some Hordeum vulgare L. growth parameters. SN Appl. Sci..

[320] Mezghani S., Bourguiba I., Hfaiedh I., Amri M. (2013). Antioxidant potential of Ulva rigida extracts: Protection of HeLa cells against H_2_O_2_ cytotoxicity. Biol. Bull..

[321] Cavuşoğlu K., Ergin H.G. (2015). Effects of humic acid pretreatment on some physiological and anatomical parameters of barley (*Hordeum vulgare* L.) exposed to salt stress. Bangladesh J. Bot..

[322] Jarošová M., Klejdus B., Kovačik J., Babula P., Hedbavny J. (2016). Humic acid protects barley against salinity. Acta Physiol. Plant..

[323] Qu Y., Bao G., Pan X., Guo J., Xiang T., Fan X., Zhang X., Yang Y., Yan B., Zhao H. (2022). Resistance of highland barley seedlings to alkaline salt and freeze–thaw stress with the addition of potassium fulvic acid. Plant Soil Environ..

[324] Alsudays I.M., Alshammary F.H., Alabdallah N.M., Alatawi A., Alotaibi M.M., Alwutayd K.M., Alharbi M.M., Alghanem S.M.S., Alzuaibr F.M., Gharib H.S. (2024). Applications of humic and fulvic acid under saline soil conditions to improve growth and yield in barley. BMC Plant Biol..

[325] Manik S.M.N., Pengilley G., Dean G., Field B., Shabala S., Zhou M. (2019). Soil and crop management practices to minimize the impact of waterlogging on crop productivity. Front. Plant Sci..

[326] Solaiman Z., Colmer T.D., Loss S.P., Thomson B.D., Siddique K.H.M. (2007). Growth responses of cool-season grain legumes to transient waterlogging. Aust. J. Agric. Res..

[327] Tian L.X., Zhang Y.C., Chen P.L., Zhang F.F., Li J., Yan F., Dong Y., Feng B.L. (2021). How does the waterlogging regime affect crop yield? A global meta-analysis. Front. Plant Sci..

[328] Ahmed F., Rafii M.Y., Ismail M.R., Juraimi A.S., Rahim H.A., Asfaliza R., Latif M.A. (2013). Waterlogging tolerance of crops: Breeding, mechanism of tolerance, molecular approaches and future prospects. BioMed Res. Int..

[329] Setter T.L., Waters I. (2003). Review of prospects for germplasm improvement for waterlogging tolerance in wheat, barley and oats. Plant Soil.

[330] Nguyen T.N., Tuan P.A., Mukherjee S., Son S., Ayele B.T. (2018). Hormonal regulation in adventitious roots and during their emergence under waterlogged conditions in wheat. J. Exp. Bot..

[331] Ploschuk R.A., Miralles D.J., Colmer T.D., Ploschuk E.L., Striker G.G. (2018). Waterlogging of winter crops at early and late stages: Impacts on leaf physiology, growth and yield. Front. Plant Sci..

[332] Kamran M., Parveen A., Ahmar S., Malik Z., Hussain S., Chattha M.S., Saleem M.H., Adil M., Heidari P., Chen J.T. (2020). An overview of hazardous impacts of soil salinity in crops, tolerance mechanisms, and amelioration through selenium supplementation. Int. J. Mol. Sci..

[333] Mohamed I.A.A., Shalby N., El-Badri M.A.A., Saleem M.H., Khan M.N., Nawaz M.A., Qin M., Agami R.A., Kuai J., Wang B.O. (2020). Stomata and xylem vessel traits improved by melatonin application contribute to enhancing salt tolerance and fatty acid composition of *Brassica napus* L.. Agronomy.

[334] Hayashi T., Yoshida T., Fujii K., Mitsuya S., Tsuji T., Okada Y., Hayashi E., Yamauchi A. (2013). Maintained root length density contributes to the waterlogging tolerance in common wheat (*Triticum aestivum* L.). Field Crops Res..

[335] Araki H., Hamada A., Hossain M.A., Takahashi T. (2012). Waterlogging at jointing and/or after anthesis in wheat induces early leaf senescence and impairs grain filling. Field Crops Res..

[336] Arduini I., Orlandi C., Pampana S., Masoni A. (2016). Waterlogging at tillering affects spike and spikelet formation in wheat. Crop Pasture Sci..

[337] Ding J.F., Liang P., Wu P., Zhu M., Li C.Y., Zhu X.K., Gao D.R., Chen Y.L., Guo W.S. (2020). Effects of waterlogging on grain yield and associated traits of historic wheat cultivars in the middle and lower reaches of the Yangtze River, China. Field Crops Res..

[338] Takahashi H., Yamauchi T., Rajhi I., Nishizawa N.K., Nakazono M. (2015). Transcript profiles in cortical cells of maize primary roots during ethylene-induced lysigenous aerenchyma formation under aerobic conditions. Ann. Bot..

[339] Voesenek L., Sasidharan R. (2013). Ethylene- and oxygen signaling drive plant survival during flooding. Plant Biol..

[340] Zeng F., Shabala L., Zhou M., Zhang G., Shabala S. (2013). Barley responses to combined waterlogging and salinity stress: Separating effects of oxygen deprivation and elemental toxicity. Front. Plant Sci..

[341] Halliwell B., Gutteridge J.M.C. (2015). Free Radicals in Biology and Medicine.

[342] Shabala S., Shabala L., Barcelo J., Poschenrieder C. (2014). Membrane transporters mediating root signaling and adaptive responses to oxygen deprivation and soil flooding. Plant Cell Environ..

[343] Foyer C.H., Noctor G. (2009). Redox regulation in photosynthetic organisms: Signaling, acclimation, and practical implications. Antioxid. Redox Signal..

[344] Rhoads D.M., Umbach A.L., Subbaiah C.C., Siedow J.N. (2006). Mitochondrial reactive oxygen species: Contribution to oxidative stress and interorganellar signaling. Plant Physiol..

[345] Khan M.I.R., Fatma M., Per T.S., Anjum N.A., Khan N.A. (2015). Salicylic acid-induced abiotic stress tolerance and underlying mechanisms in plants. Front. Plant Sci..

[346] Zhao P., Lu G.-H., Yang Y.-H., Pandey G.K. (2017). Salicylic acid signaling and its role in responses to stresses in plants. Mechanism of Plant Hormone Signaling under Stress II.

[347] Li Z.-G., Gong M., Xie H., Yang L., Li J. (2012). Hydrogen sulfide donor sodium hydrosulfide-induced heat tolerance in tobacco (*Nicotiana tabacum* L.) suspension cultured cells and involvement of Ca^2+^ and calmodulin. Plant Sci..

[348] Zhang H., Hu L.Y., Li P., Hu K.D., Jiang C.X., Luo J.P. (2010). Hydrogen sulfide alleviated chromium toxicity in wheat. Biol. Plant..

[349] Koramutla M.K., Tuan P.A., Ayele B.T. (2022). Salicylic acid enhances adventitious root and aerenchyma formation in wheat under waterlogged conditions. Int. J. Mol. Sci..

[350] Yamauchi T., Watanabe K., Fukazawa A., Mori H., Abe F., Kawaguchi K., Oyanagi A., Nakazono M. (2014). Ethylene and reactive oxygen species are involved in root aerenchyma formation and adaptation of wheat seedlings to oxygen-deficient conditions. J. Exp. Bot..

[351] Nawaz M.A., Huang Y., Bie Z., Ahmed W., Reiter R.J., Niu M., Hameed S. (2016). Melatonin: Current status and future perspectives in plant science. Front. Plant Sci..

[352] Ma S., Gai P., Geng B., Wang Y., Ullah N., Zhang W., Zhang H., Fan Y., Huang Z. (2022). Exogenous melatonin improves waterlogging tolerance in wheat through promoting antioxidant enzymatic activity and carbon assimilation. Agronomy.

[353] Moustafa-Farag M., Mahmoud A., Arnao M.B., Sheteiwy M.S., Dafea M., Soltan M., Elkelish A., Hasanuzzaman M., Ai S. (2020). Melatonin-induced water stress tolerance in plants: Recent advances. Antioxidants.

[354] Quaratiello G., Risoli S., Antichi D., Pellegrini E., Nali C., Lorenzini G., Pampana S., Pisuttu C., Tonelli M., Cotrozzi L. (2024). Exogenous melatonin application helps late-sown durum wheat to cope with waterlogging under Mediterranean environmental conditions. Physiol. Plant..

[355] Teng Z., Zheng W., Jiang S., Hong S.-B., Zhu Z., Zang Y. (2022). Role of melatonin in promoting plant growth by regulating carbon assimilation and ATP accumulation. Plant Sci..

[356] Talaat N.B. (2021). Polyamine and nitrogen metabolism regulation by melatonin and salicylic acid combined treatment as a repressor for salt toxicity in wheat (*Triticum aestivum* L.) plants. Plant Growth Regul..

[357] Ahmad I., Zhu G., Zhou G., Liu J., Younas M.U., Zhu Y. (2023). Melatonin role in plant growth and physiology under abiotic stress. Int. J. Mol. Sci..

[358] Mfarrej M.F.B., Wang X., Saleem M.H., Hussain I., Rasheed R., Ashraf M.A., Iqbal M., Chattha M.S., Alyemeni M.N. (2022). Hydrogen sulphide and nitric oxide mitigate the negative impacts of waterlogging stress on wheat (*Triticum aestivum* L.). Plant Biol..

[359] Ahmad P., Ahanger M.A., Alyemeni M.N., Wijaya L., Alam P. (2018). Exogenous application of nitric oxide modulates osmolyte metabolism, antioxidants, enzymes of ascorbate–glutathione cycle and promotes growth under cadmium stress in tomato. Protoplasma.

[360] Habib N., Ashraf M., Shahbaz M. (2013). Effect of exogenously applied nitric oxide on some key physiological attributes of rice (*Oryza sativa* L.) plants under salt stress. Pak. J. Bot..

[361] Procházková D., Haisel D., Wilhelmová N., Pavlíková D., Száková J. (2013). Effects of exogenous nitric oxide on photosynthesis. Photosynthetica.

[362] Kisaakye E., Botwright Acuña T., Johnson P., Shabala S. Improving wheat growth and nitrogen-use efficiency under waterlogged conditions. Proceedings of the 18th Australian Society of Agronomy Conference.

[363] Robertson D., Zhang H., Palta J.A., Colmer T.D., Turner N.C. (2009). Waterlogging affects the growth, development of tillers, and yield of wheat through a severe, but transient nitrogen deficiency. Crop Pasture Sci..

[364] Mathers N.J., Nash D.M., Gangaiya P. (2007). Nitrogen and phosphorus exports from high rainfall zone cropping in Australia: Issues and opportunities for research. J. Environ. Qual..

[365] Rasaei A., Ghobadi M.E., Jalali-Honarmand S., Ghobadi M., Saeidi M. (2012). Impacts of waterlogging on shoot apex development and recovery effects of nitrogen on grain yield of wheat. Eur. J. Exp. Biol..

[366] Wu J.-D., Li J.-C., Wei F.-Z., Wang C.-Y., Zhang Y., Sun G. (2014). Effects of nitrogen spraying on the post-anthesis stage of winter wheat under waterlogging stress. Acta Physiol. Plant..

[367] Chhabra V., Sreethu S., Kaur G., Singh A., Kaur M., Siddiqui M.H., Gupta R.K. (2025). Response of wheat crop to waterlogged conditions under different land configurations and nutrient management. Sci. Rep..

[368] Ali Q., Perveen R., El-Esawi M.A., Ali S., Hussain S.M., Amber M., Iqbal N., Rizwan M., Alyemeni M.N., El-Serehy H.A. (2020). Low doses of Cuscuta reflexa extract act as natural biostimulants to improve the germination vigor, growth and grain yield of wheat grown under water stress: Photosynthetic pigments, antioxidative defense mechanisms and nutrient acquisition. Biomolecules.

[369] Noman A., Ali Q., Naseem J., Javed M.T., Kanwal H., Islam W., Aqeel W., Khalid N., Zafar S., Tayyeb M. (2018). Sugar beet extract acts as a natural biostimulant for physio-biochemical attributes in water-stressed wheat (*Triticum aestivum* L.). Acta Physiol. Plant..

[370] Pang J., Ross J., Zhou M., Mendham N., Shabala S. (2007). Amelioration of detrimental effects of waterlogging by foliar nutrient sprays in barley. Funct. Plant Biol..

[371] Shiono K., Ejiri M., Shimizu K., Yamada S. (2019). Improved waterlogging tolerance of barley (*Hordeum vulgare*) by pretreatment with ethephon. Plant Prod. Sci..

[372] Yamauchi T., Noshita K., Tsutsumi N. (2021). Climate-smart crops: Key root anatomical traits that confer flooding tolerance. Breed. Sci..

[373] Zhang W., Wen C.-K. (2010). Preparation of ethylene gas and comparison of ethylene responses induced by ethylene, ACC, and ethephon. Plant Physiol. Biochem..

[374] Chen J., Zhao C., Harrison M.T., Zhou M. (2024). Nitrogen fertilization alleviates barley (*Hordeum vulgare* L.) waterlogging. Agronomy.

[375] Kaur G., Nelson K.A., Motavalli P.P. (2018). Early-season soil waterlogging and nitrogen fertilizer source impacts on corn nitrogen uptake and apparent nitrogen recovery efficiency. Agronomy.

[376] Savci S. (2012). An agricultural pollutant: Chemical fertilizer. Int. J. Environ. Sci. Dev..

[377] Peuke A.D., Jeschke W.D., Hartung W. (1998). Foliar application of nitrate or ammonium as sole nitrogen supply in *Ricinus communis*. II. The flows of cations, chloride and abscisic acid. New Phytol..

[378] Xue G.-P., Sadat S., Drenth J., McIntyre C.L. (2014). The heat shock factor family from *Triticum aestivum* in response to heat and other major abiotic stresses and their role in regulation of heat shock protein genes. J. Exp. Bot..

[379] Byrt C.S., Platten J.D., Spielmeyer W., James R.A., Lagudah E.S., Dennis E.S., Tester M., Munns R. (2007). HKT1;5-like cation transporters linked to Na^+^ exclusion loci in wheat, Nax2 and Kna1. Plant Physiol..

